# Supplementary material for: Reduction of NAD and NMN on mineral surfaces with H2 reveals a functional role for the AMP moiety in a prebiotic context
Source: Commun Chem. 2025 Oct 30;8:318. doi: 10.1038/s42004-025-01752-3 (PMC12575802; doi:10.1038/s42004-025-01752-3)
Supplement: Supplementary file 2 — Supplementary Information [file 42004_2025_1752_MOESM2_ESM.pdf]

Supplementary Information for:

Reduction of NAD and NMN on mineral surfaces with H_2 reveals a functional role for the AMP moiety in prebiotic evolution

Delfina P. Henriques Pereira*[a,b], Xiulan Xie[c], Sarah V. Stewart[d,e], Zainab Subrati[a,b], Tuğçe Beyazay[f], Nicole Paczia[g], Jürgen Belz[h], Kerstin Volz[h], Valentina Erastova[d,e], Harun Tüysüz[f,i], Martina Preiner*[a,b]

[a] Microcosm Earth Center, Max-Planck-Institute for Terrestrial Microbiology and Philipps-University Marburg, Marburg, German.

[b] Geochemical Protoenzymes Research Group, Max-Planck-Institute for Terrestrial Microbiology, Marburg, Germany.

[c] Department of Chemistry, Philipps University Marburg, Marburg, Germany.

[d] School of Chemistry, University of Edinburgh, Edinburgh, United Kingdom.

[e] UK Centre for Astrobiology, School of Physics and Astronomy, University of Edinburgh, Edinburgh, United Kingdom.

[f] Heterogeneous Catalysis, Max-Planck-Institut für Kohlenforschung, Mülheim an der Ruhr, Germany.

[g] Metabolomics and small molecule mass spectrometry, Max-Planck-Institute for Terrestrial Microbiology, Marburg, Germany.

[h] Department of Physics, Philipps University Marburg, Marburg, Germany.

[i] IMDEA Materials Institute, Madrid, Spain.

*Corresponding authors: Martina Preiner, Delfina P. Henriques Pereira
Email: martina.preiner@mpi-marburg.mpg.de; delfina.pereira@mpi-marburg.mpg.de

Keywords: Adenosine-derived cofactors; hydrogen; emergence of life; mineral catalysis; protometabolism

This PDF file includes:

Supplementary Methods
Equations S1–3
Figures S1–74
Tables S1–27
Scheme S1–18
References

Supplementary Methods

Liquid Chromatography Mass Spectroscopy (LC-MS). Samples were prepared from the supernantant of a reaction with 36 µmol NMN, 36 µmol Fe^0 (nanopowder) in 3 mL of 0.133 M PBS (pH 8.5). The reaction ran for 4 h at 40 °C in a high pressure reactor with 5 bar of H_2. Afterwards the supernatant was diluted 1:200 with HPLC grade H_2O. 2 replicas were measured and compared to the supernatant of the control (without metal powder) and a standard with equivalent amounts of 1,4-NMN to NMN in the samples. The chromatographic separation was performed on a Thermo Scientific Vanquish high performance liquid chromatography (HPLC) System using a SeQuant ZIC-pHILIC column (150 × 2.1 mm, 5 µm particle size, peek coated, Merck) connected to a guard column of similar specificity (20 × 2.1 mm, 5 µm particle size, Phenomenex) a constant flow rate of 0.1 mL/min with mobile phase A with mobile phase comprised of 10 mM ammonium acetate in water, pH 9, supplemented with medronic acid to a final concentration of 5 µM (A) and 10 mM ammonium acetate in 90:10 acetonitrile to water, pH 9, supplemented with medronic acid to a final concentration of 5 µM (B) at 40 °C. The injection volume was, dependent on the expected analyte concentration, between 1 µl and 5 µl. The mobile phase profile consisted of the following steps and linear gradients: 0 – 1 min constant at 75% B; 1 – 6 min from 75 to 40% B; 6 to 9 min constant at 40% B; 9 – 9.1 min from 40 to 75% B; 9.1 to 20 min constant at 75% B. A Thermo Scientific ID-X Orbitrap mass spectrometer was used in negative ionization mode with an electrospray ionization source and the following conditions: H^-ESI spray voltage at 5500 V, sheath gas at 25 arbitrary units, auxiliary gas at 5 arbitrary units, no sweep gas, ion transfer tube temperature at 275 °C, and Vaporizer temperature at 75 °C.detection was performed in full scan mode using the orbitrap mass analyzer at a mass resolution of 120 000 in the mass range 330–360 (m/z). The extracted ion chromatograms of the $[M-H]^-$ were generated using Freestyle software (Thermo Scientific) applying a mass accuracy of 5 ppm. Comparative predicted natural isotope distribution was calculated using ChemCalcz. Peak spectra were extracted from the apex of the peak. A Background subtraction was performed from an injection of water using the spectrum extracted at the same retention time.

Model Construction The nickel spherical particle was built using CHARMM-GUI Nanomaterial Modeler[1]. The sphere is 2.8 nm in diameter comprising from 1289 Ni atoms and is assigned INTERFACE force field parameters(2).[2] The organic molecules, NAD^+, NMN, NADH and NMNH, were set up using Marvin Sketch 23.17 by ChemAxon (http://www.chemaxon.com). The protonation states were assigned for solution of pH 8.5, in line with experiment, where all organic molecules are deprotonated at both phosphate -OH groups resulting in two negative sites. Additionally, NAD^+ and NMN are positive at the N atom on the nicotinamide ring and NADH and NMNH are reduced at the C-4 position on the nicotinamide ring (resulting in a neutral N), as seen in **Fig. S30**. The molecules were subsequently assigned CHARMM36 force fields using the CHARMM-GUI Ligand Reader and Modeler[3,4]. TIP3P water was used in all simulations and the ions were described using the Pengfei Li parameters[5]. Each of the systems contained Ni sphere, 10 organic molecules (either NAD^+, NMN, NADH and NMNH), 5 buffer HPO_4^{2-} molecules and water. Number of water molecules added was sufficient to fill in a 6.9 × 6.9 × 6.9 nm^3 simulation box at a standard density of 1000 g l^{-1}. Na^+ ions were added to charge-balance the system. To ensure sufficient statistics were obtained and that the simulations were not biased by the starting configurations, all systems were run in triplicates with new positions of organics, buffer and ions in the box. Furthermore, since NAD^+ is known to exist in both folded and unfolded configurations, we

ran a simulation where all NAD^+ molecules inserted into a box were folded. We also performed a simulation of NAD^+ in water without Ni surface to study the folding dynamics. Summary of the systems are given in the **Table S14**.

Molecular Dynamics Simulations All simulations were carried out using GROMACS 2024.3(6). Every system after set up has undergone energy minimization using steepest descent algorithm and ensuring that maximum force on any one atom is less than 500 kJ mol^{-1} nm^{-1}. This was followed by a molecular dynamics simulation of 50 ns with 1 fs timestep in isothermal-isobaric ensemble. The temperature was set to 313 K using velocity-rescale thermostat with time coupling of 1 ps, the pressure was set to 1 bar controlled with C-rescale barostat and a coupling time of 10 ps. These settings were chosen to ensure closest match to the experiment. In all simulations neighbor searching was performed every 10 steps, electrostatic and van der Waals interactions were computed using Particle-Mesh-Ewald algorithm with geometric combination rules, Verlet cutoff-scheme and 1.4 nm cutoff distances. The LINCS algorithm was used for H-bond constraints.

Analysis and Visualization Analyses were performed using GROMACS 2024.3 tools[6], with data postprocessed and plotted with in-house tools using the matplotlib Python package[7]. Simulation renderings were produced with VMD 1.94[8] with the colours as follows, unless stated otherwise: Ni=silver surface, C=cyan, H=white, N=blue, O=red, P=gold. Ring structures are shaded in for visualisation. Bulk water molecules are not shown for clarity.The convergence of the simulated systems was determined through root mean square deviation (RMSD) analysis, using *gmx rmsd* tool. The system assumed converged when RMSD for each individual component has reached a plateau and remained in plateau for the remaining of simulation. Therefore, the last 20 ns from a total of 50 ns simulation were used for further analysis. Data is presented as average of all repeats.The radial distribution function (RDF) analysis was performed using *gmx rdf* tool. Specifically, we present distances from Ni surface atoms (selected using *-surf* option) to the C-4 of the nicotinamide ring. Coordination numbers as a function of distance were also calculated. RDF informs us on the local surface structuring of the organic molecules, where the height of the peak is related to increase ordering at the given distance. The folding dynamics of NAD^+ and NADH were analyzed through calculation of molecules' radius of gyration, using *gmx gyrate* tool, which allows monitoring of changes in the molecules' configuration between folded and expanded throughout the simulation. The data from the last 20 ns was accumulated into histograms, which are presented. Furthermore, we calculated the distances between nicotinamide and adenosine rings, using *gmx mindist* tool, and the data from the last 20 ns was accumulated into histograms, which are also presented. The data informs us on the distances for the open and closed configuration. From here, using the distances less than 0.6 nm, we calculated the number of contacts between rings. The data informs us on the number and evolution of contacts between rings, with 9 contacts being the maximum number, when two rings are in parallel with each other.

Cyclic Voltammetry. The reduction potential of NAD and NMN were compared through cyclic voltammetry performed at 1 mM of each compound in H_2O at 25ºC using as electrolyte disodium hydrogen phosphate and potassium phosphate (0.133 M PBS), at a scan rate of 50 mV/s. A three-electrode electrochemical cell has been used: glassy carbon as the working electrode, platinum wire as an auxiliary electrode, and $Ag/AgNO_3$ (0.01 M) as a reference electrode.

Scanning Transmission Electron Microscope (STEM). In preparation for the STEM measurement, 1 mg of Ni-Fe-particles where dispersed in 400 µL methanol (HPLC grade; Fisher Scientific) using an ultrasonic bath (Bandelin) for 5 s. Subsequently, 20 µL the dispersion where diluted with additional 90 µL of methanol, dispersed in the ultrasonic bath again. A drop of the dispersion was placed on the Cu 300 Mesh lacey carbon grid (EMS) and left to dry. In the case of particles recovered after a reaction, the particles were washed with water (HPLC grade, Fisher Scientific), dried and handled accordingly to pristine particles above. Measurements of drop-cast NiFe particles were carried out using an aberration-corrected JEOL JEM-2200FS STEM equipped with a Bruker XFlash 5060 Energy Dispersive X-ray Spectrometer (EDX) operated in analytical mode with a spatial resolution of approximately 1-2 Ångstroms.

Henry's Law

Equation S1 H_2 concentration

$$H^{cp} = \frac{c_a}{p}$$

H^{cp}:Henry's constant

c_a: concentration

p: pressure

Equation S2 Henry's constant for H_2:

$$H^{cp}(H_2, 298\,K) = 7.8 \times 10^{-4} \frac{mol}{L \times atm}$$

Equation S3 Temperature dependency of Henry's constant:

$$H^{cp}(H_2, T_2) = H^{cp}(H_2, 298\,K) \times e^{(500\left(\frac{1}{T_2} - \frac{1}{298\,K}\right))}$$

Heterogeneous catalysis of NAD^+ reduction with H_2 and Ni/Fe alloys

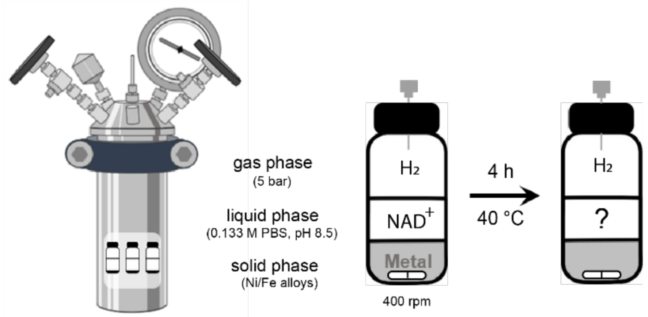

Scheme S1 The reduction of NAD^+ with Ni/Fe alloys was tested with the protocol described in detail in Methods, and according to the scheme above. The amount of metal and NAD^+ was 36 μmol, which reacted together for 4 h, at 40 °C, under alkaline conditions and 5 bars of H_2. The same reaction was made under Ar as a control.

Table S1 For quantification of each compound, a peak was selected in their spectra. The values listed a bellow indicate the ppm value where the peak can be found at approximately pH 8.5 (0.133M PBS).

Molecule	NAD^+	Nam	1,6-NADH	1,4-NADH
δ (ppm) for quantification	9.12	8.93	7.11	6.94

Table S2 After 4 h under 5 bar of H_2, samples with different nanoparticular Ni/Fe alloys yielded different amounts of 1,4-NADH, 1,6-NADH and nicotinamide (Nam), from the starting material NAD^+, as listed below. The starting metal and cofactor were 36 μmol mixed in 3 mL of 0.133 M PBS (pH 8.5), as shown in **Scheme S1**. The yields were calculated relative to the metal-free sample (100% NAD^+). Reactions with nNi, nNi_3Fe, and nNiFe were performed with six replicas; Fe^0 and w/o metal were done with five replicas; $nNiFe_3$ amounted to four. To determine the turn over frequency (TOF) of each reaction, 1,4-NADH and 1,6-NADH were considered as products and the total amount of metal atoms were considered as the amount of catalyst, instead of the number of molecules.

	H_2	NAD^+	SD	1,4-NADH	SD	1,6-NADH	SD	Nam	SD	TOF [s^{-1}]
4h	nNi^0	72.63%	3.5%	3.68%	0.5%	1.19%	0.2%	6.37%	0.3%	3.57E-06
	nNi_3Fe	61.20%	2.7%	14.09%	1.8%	6.84%	1.1%	7.47%	0.2%	1.54E-05
	nNiFe	31.10%	2.1%	19.66%	2.4%	7.90%	1.2%	7.88%	0.7%	2.02E-05
	$nNiFe_3$	25.59%	4.4%	40.60%	1.8%	16.70%	0.6%	4.48%	0.1%	3.02E-05
	nFe^0	52.23%	7.6%	6.01%	1.1%	0.00%	0.0%	7.04%	0.6%	4.41E-06

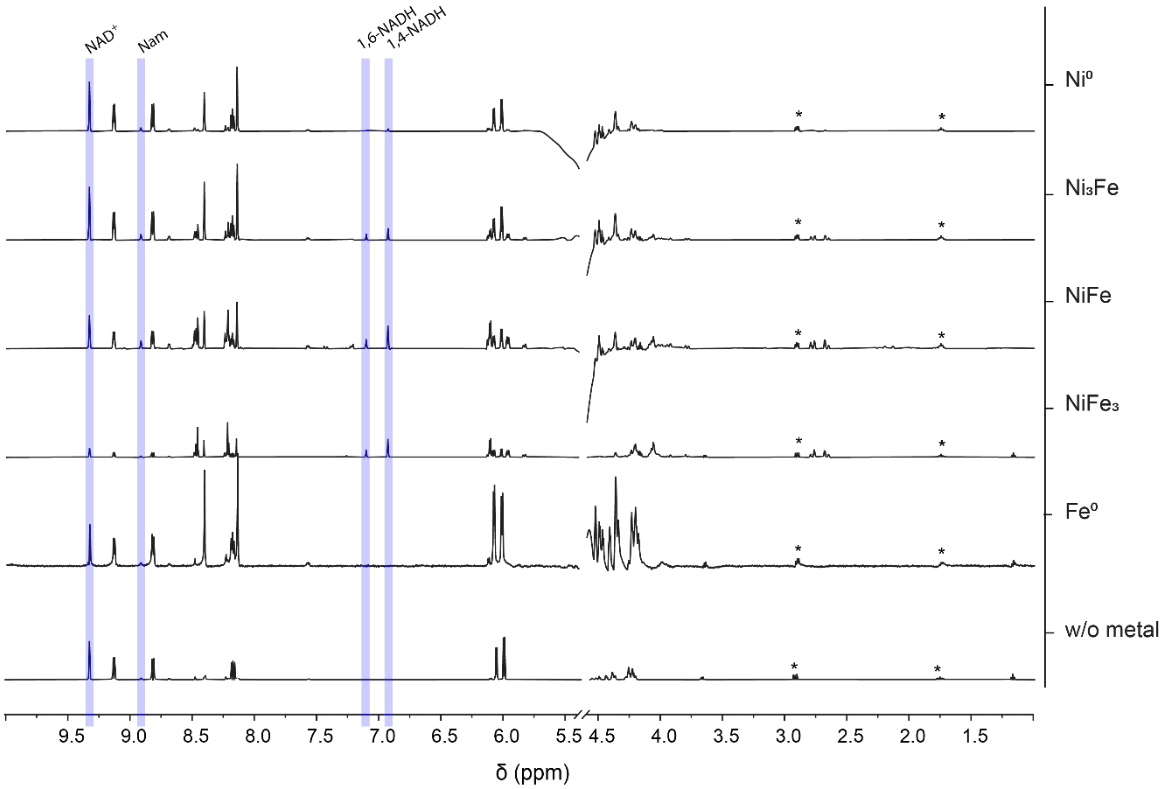

Fig. S1 The NMR spectra of replica samples of NAD^+ in PBS (0.133 M, pH 8.5) after 4 h with 5 bar of H_2 and a metal 1:1 cofactor ratio, as shown in **Scheme S1**, are stacked together in this figure. The metal used in each sample is indicated on the right side of each spectra. After 4h reacting, the supernatant was collected and DSS added as an internal standard. The spectra were edited to only include relevant peaks, having been removed a DSS peak at 0 ppm and water peak at 4.8 ppm. No other peaks were found in the areas removed. Some DSS peaks are still visible (*). The peaks used for qualitative analysis and subsequent qNMR are highlighted in blue, according to **Table S1**.

Table S3 After 4 h under 5 bar of Ar, samples with different nanoparticular Ni/Fe alloys yielded different amounts of 1,4-NADH, 1,6-NADH and Nam, from the starting material NAD^+, as listed below. The starting metal and cofactor were 36 µmol mixed in 3 mL of 0.133 M PBS (pH 8.5), as shown in **Scheme S1** with Ar. The yields were calculated relative to the metal-free sample (100% NAD^+). Reactions with nNi, nNi_3Fe, and nNiFe were performed with six replicas; nFe^0 and w/o metal were done with five replicas; $nNiFe_3$ amounted to four. To determine the TOF of each reaction, 1,4-NADH and 1,6-NADH were considered as products and the total amount of metal atoms were considered as the amount of catalyst, instead of the number of molecules.

	Ar	NAD^+	SD	1,4-NADH	SD	1,6-NADH	SD	Nam	SD	TOF [s^{-1}]
4h	nNi^0	80.94%	1.7%	0.00%	0.0%	0.00%	0.0%	7.48%	0.2%	0.00E+00
	nNi_3Fe	62.68%	8.0%	0.00%	0.0%	0.00%	0.0%	6.61%	1.5%	0.00E+00
	nNiFe	85.24%	2.5%	0.00%	0.0%	0.00%	0.0%	8.48%	0.3%	0.00E+00
	$nNiFe_3$	88.84%	1.2%	4.43%	0.9%	1.64%	0.3%	4.55%	0.0%	3.20E-06
	nFe^0	77.00%	6.0%	5.44%	0.9%	0.00%	0.0%	20.33%	2.3%	3.99E-06

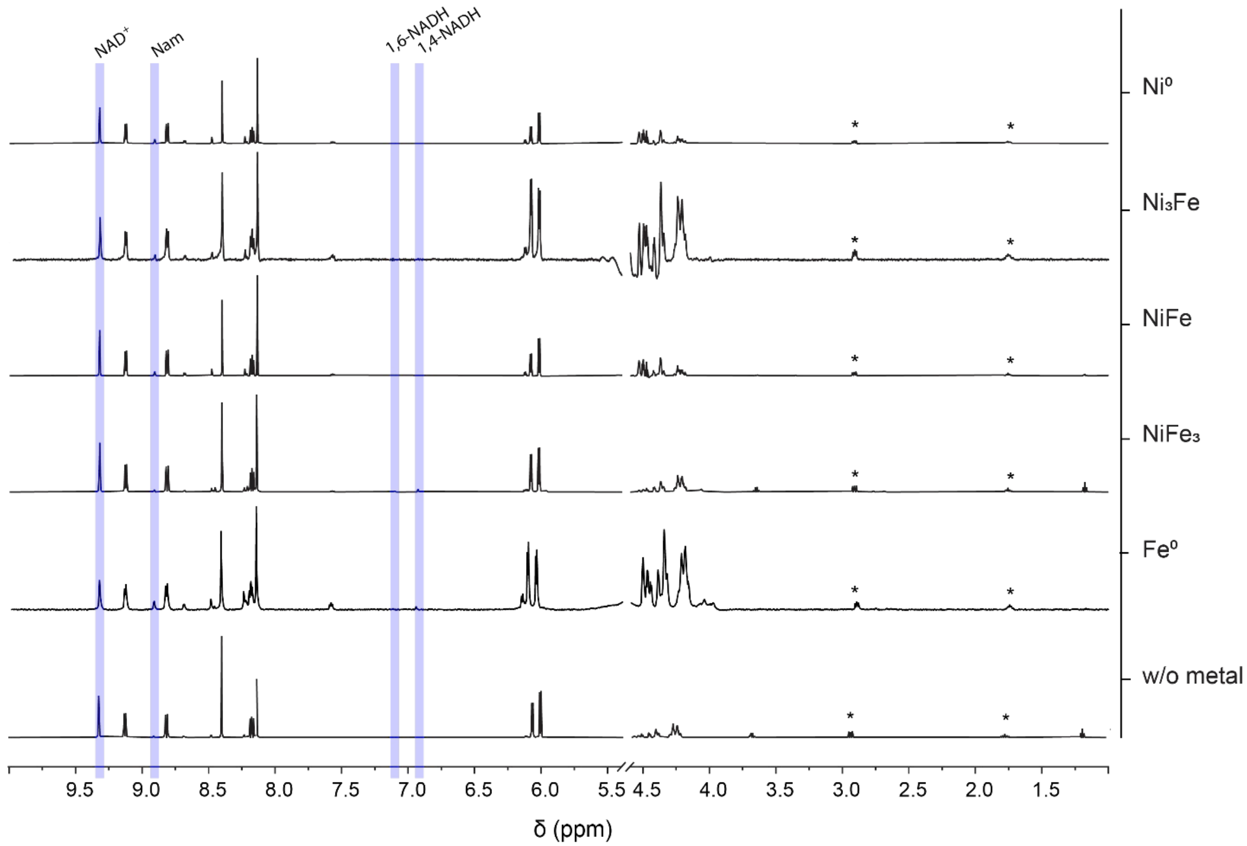

Fig. S2 The NMR spectra of replica samples of NAD^+ in PBS (0.133 M, pH 8.5) after 4 h with 5 bar of Ar and a metal 1:1 cofactor ratio, as shown in **Scheme S1** with Ar, are stacked together in this figure. The nanoparticular metalpowder used in each sample is indicated on the right. After 4h reacting, the supernatant was collected and DSS added as an internal standard. The spectra were edited to only include relevant peaks, having been removed a DSS peak at 0 ppm and water peak at 4.8 ppm. No other peaks were found in the areas removed. Some DSS peaks are still visible (*). The peaks used for qualitative analysis and subsequent qNMR are highlighted in blue, according to **Table S1**.

Specificity of each metal to 1,4-NADH production

To further understand the accumulation of 1,6-NADH, the reaction was reproduced under an inert N_2 atmosphere, and starting with a solution of 1,4-NADH instead of NAD^+ and with duplicates.

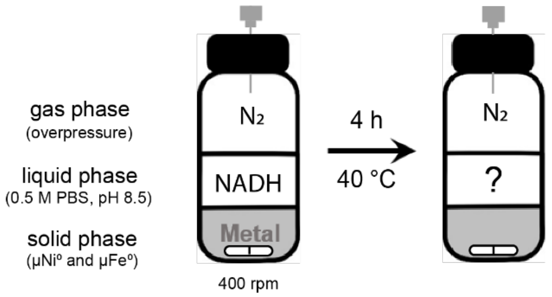

Scheme S2 The conversion of 1,4-NADH to 1,6-NADH was tested according to the scheme above. The amount of 1,4-NADH was 36 μmol, and 50 times more metal was added to each vial (μFe^0 and μNi^0) which reacted together for 4 h, at 40 °C, under alkaline conditions and a N_2 overpressure.

Table S4 After 4 h under an overpressure of N_2, as shown in **Scheme S2**, samples with μNi and μFe yielded different amounts of 1,4-NADH and 1,6-NADH, from the starting material 1,4-NADH. The starting cofactor was 36 μmol mixed in 3 mL of 0.5 M PBS (pH 8.5) and with 1.8 mmol of μFe or μNi. To determine the specificity of each reaction to reduce NAD in the 4th of 6th carbon of the nicotinamide moiety, the amount of 1,6-NADH was normalized to the total amount of NADH in the sample (1,4-NADH + 1,6-NADH). All conditions had duplicates.

N_2	1,6-NADH/NADH	SD
control	15.3%	0,00%
μFe^0	10,11%	0,78%
μNi^0	12,91%	0,33%

Starting from a solution of only 1,4-NADH, all samples yielded more 1,6-NADH than when starting from NAD^+ (**Table S4**). Additionally, the metal-containing experiments did not accumulate more 1,6-NADH than the control.

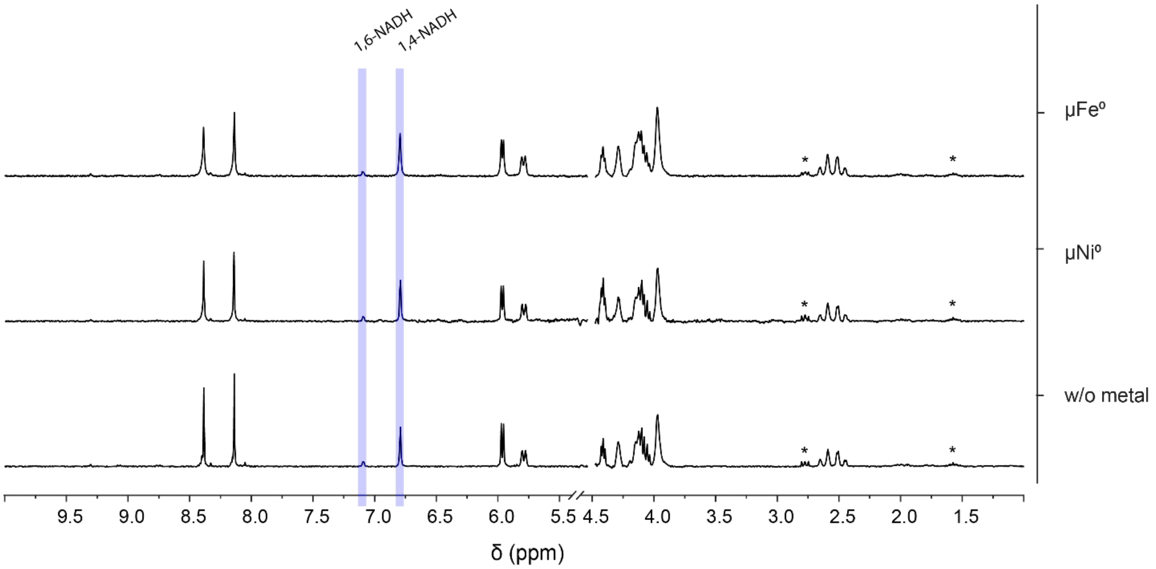

Fig. S3 The NMR spectra of replica samples of 1,4-NADH in PBS (0.5 M, pH 8.5), as shown in **Scheme S2**, are stacked together in this figure. The metal used in each sample is the micropowder indicated on the right. After 4h reacting, the supernatant was collected and DSS added as an internal standard. The spectra were edited to only include relevant peaks, having been removed a DSS peak at 0 ppm and water peak at 4.8 ppm. No other peaks were found in the areas removed. Some DSS peaks are still visible (*). The peaks used for qualitative analysis and subsequent qNMR are highlighted in blue, according to **Table S1.**

Looking again at the experiments described with NAD, the only other reduction product made other than 1,4-NADH, in significant amounts and identified through 2D-NMR, was 1,6-NADH. The amount of 1,6-NADH relative to the total amount of NADH detected in each sample was calculated and presented in the table below.

Table S5 After 4 h under 5 bar of H_2, as shown in **Scheme S1**, samples with different Ni/Fe alloys yielded different amounts of 1,4-NADH and 1,6-NADH, from the starting material NAD^+. The starting amount of cofactor was 36 µmol mixed in 3 mL of PBS (pH 8.5), with equimolar amounts of metal nanopowder (0.133 M PBS buffer) or 50 times more if using micropowder (0.5 M PBS buffer). The 1,6-NADH and 1,4-NADH yields were collected from **Table S2** and **Table S21**. To determine the specificity of each reaction to reduce NAD in the 4th or 6th carbon of the nicotinamide moiety, the amount of 1,6-NADH was normalized to the total amount of 1,x-NADH in the sample (1,4-NADH + 1,6-NADH). Calculating mere surface area differences between nNi and µNi powder results in a ratio of roughly 50:1, for nFe and µFe the ratio is 1:1400.

H_2	1,6-NADH/NADH	SD
µNi^0	20,91%	0,34%
nNi^0	24,34%	1,36%
nNi_3Fe	32,63%	0,82%
nNiFe	28,58%	1,19%
n$NiFe_3$	29,15%	0,20%
nFe^0	0,00%	0,00%
µFe^0	16,50%	0,80%

According to **Table S5**, micropowder metals tend to lead to the accumulation of less 1,6-NADH compared to nanopowders. 1,6-NADH was not detected in samples with nFe^0, most likely due to the low NADH yield and high amounts of dissolved paramagnetic metal ions leading to line broadening. Contrastingly, n$NiFe_3$ is the only metal to produce 1,6-NADH under Ar, due to its comparably high TOF (s. **Table S3**). The ratio of 1,6-NADH to NADH seems to be maintained independently of the gas phase and yield between 20 and 30%.

Given the differences observed between metals and control, it is clear that metals can influence the production of 1,6-NADH, but it also seems that in the case of micropowders, it mostly results from an equilibrium reaction of NADH in solution.

Scanning Transmission Electron Microscope of Ni-Fe-nanopowders

A

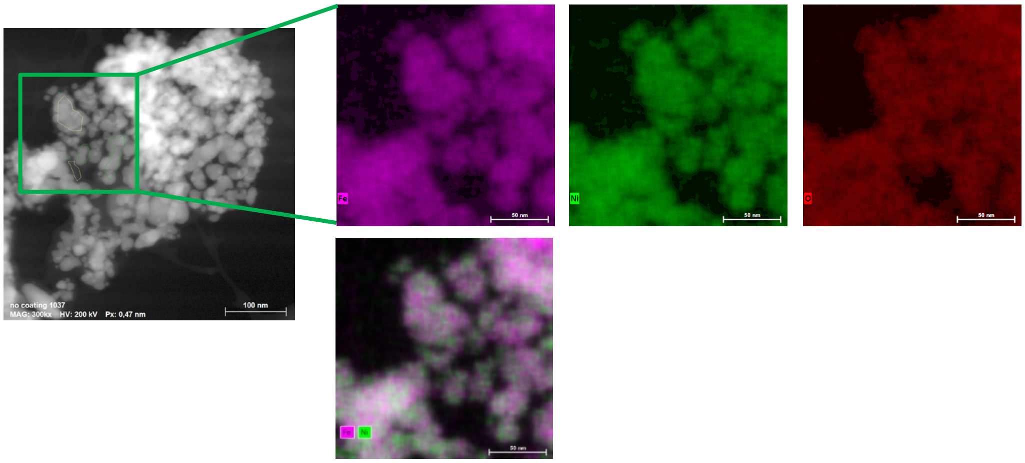

B

EDS	1		0,10	C	O 22,0	Na	Mg 0,7	P 3,1	S 2,1	Cl 0,7	Ca 3,3	Fe 36,1	Ni 32,0
EDS	2		0,00	C	O 50,7	Na 2,4	Mg 0,9	P 6,9	S	Cl 0,5	Ca 11,3	Fe 17,6	Ni 9,7
EDS	3		0,00	C	O 13,2	Na 0,5	Mg 0,7	P 1,5	S 0,8	Cl 0,0	Ca 2,6	Fe 46,3	Ni 34,3
EDS	4		0,02	C	O 14,0	Na 1,0	Mg 1,0	P 1,2	S 0,7	Cl	Ca 3,6	Fe 45,7	Ni 32,8

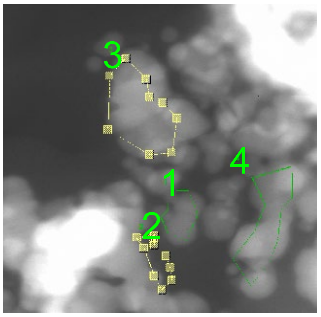

Fig. S4 NiFe (1:1) nanopowder pre-reaction STEM observation. (A) STEM-EDS analyses show that NiFe nanopowder shows a mostly even distribution of nickel an iron throughout the nanoparticles. (B) On average, there is slightly more Fe than Ni. A thin oxide layer covers the particles, probably shielding the particles from further oxidation. In comparison with $NiFe_3$ particles (**Fig. S5**). NiFe shows less Fe-rich regions.

A

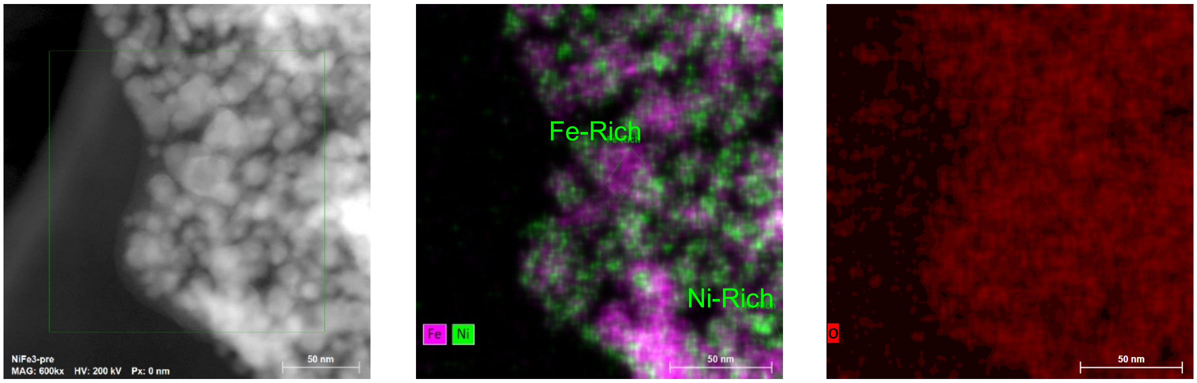

EDS	In Total	0,01	100,0	C	O 6,6	Na	Mg	Al	Si	P	S	Cl	Ca	Fe 64,6	Ni 28,8	Cu
EDS	Ni-Rich	0,00	100,0	C	O 6,8	Na	Mg	Al	Si	P	S	Cl	Ca	Fe 44,9	Ni 48,3	Cu
EDS	Fe-Rich	0,00	100,0	C	O 5,1	Na	Mg	Al	Si	P	S	Cl	Ca	Fe 83,8	Ni 11,0	Cu

B

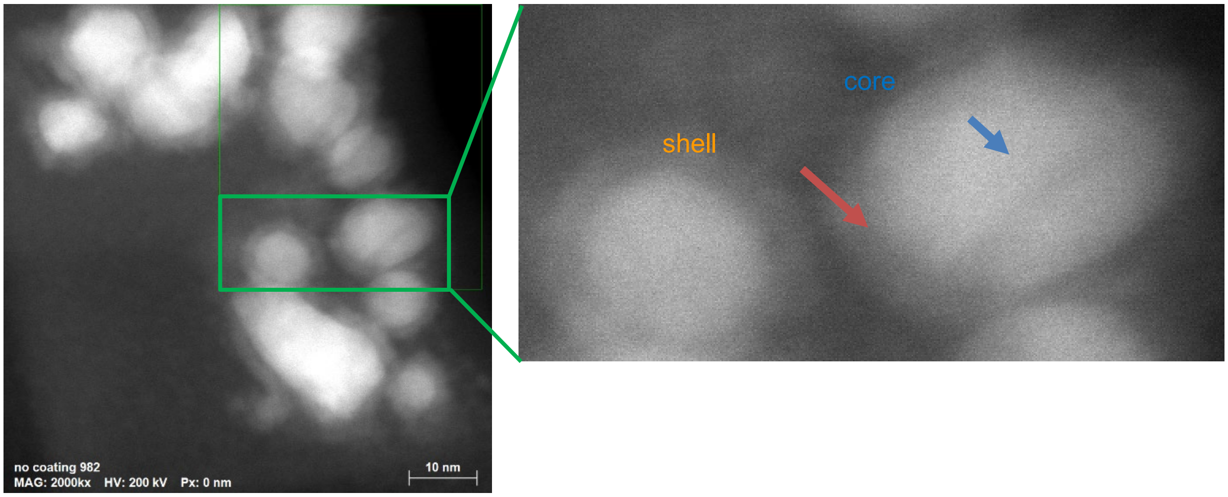

EDS	shell	0,12	100,0	C	O 37,5	Na 14,6	Mg 4,8	P 0,5	S 1,7	Cl 4,2	Ca 8,8	Fe 16,8	Ni 11,1
EDS	core	0,08	100,0	C	O 13,3	Na 3,4	Mg	P 1,7	S 1,7	Cl 1,5	Ca 5,2	Fe 35,3	Ni 37,8

Fig. S5 $NiFe_3$ (1:3) nanopowder pre-reaction STEM observation. (A) STEM-EDS analyses show that $NiFe_3$ nanopowder shows the overall distribution of Ni and Fe is 1:3, but we find both metals are not distributed completely evenly, probably leading to the well hydrogenation yields in the reactions. There are iron rich areas that can predominantly be oxidized, delivering nascent H_2 to the NiFe regions of the same powder. (B) A thin oxide layer covers the particles, probably shielding them from further oxidation before the reaction.

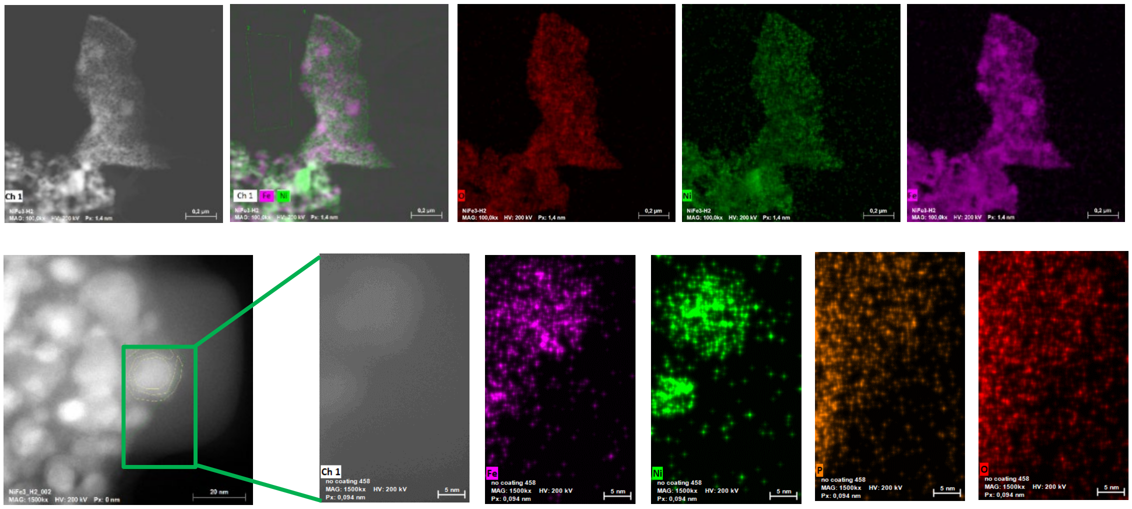

EDS	ROI	0,01	C	O 42,9	Na 1,4	Mg 1,7	Al 0,5	Si 0,6	P 9,4	S 0,3	Cl 1,0	Ca 4,9	Fe 24,8	Ni 12,5
EDS	shell	0,01	C	O 52,9	Na 2,1	Mg 2,0	Al 1,6	Si 0,3	P 10,8	S 0,0	Cl 1,0	Ca 5,8	Fe 20,5	Ni 3,1
EDS	core	0,01	C	O 29,1	Na 0,3	Mg 0,4	Al	Si 0,3	P 6,7	S 0,6	Cl 0,7	Ca 3,3	Fe 34,8	Ni 23,8

Fig. S6 $NiFe_3$ (1:3) nanopowder after reaction in H_2 atmosphere – STEM observation. STEM-EDS analyses show that the Fe of $NiFe_3$ nanopowder gets associated with the phosphate used in the buffer. This is being determined via the ratios between Fe, P and O (1.5:1:4) in the elemental analysis of the shells forming around the nanoparticles. From previous work[9] we know, that Fe is capable of producing nascent hydrogen while being oxidized, these findings are a more direct proof of this happening. The growing phosphate layer could also ultimately lead to the Fe(0) containing minerals to decrease their reaction-promoting ability – we posit that the H_2 in the atmosphere can re-cycle the oxidized Fe back to Fe^0 and thus keep the reaction going for a longer period of time.

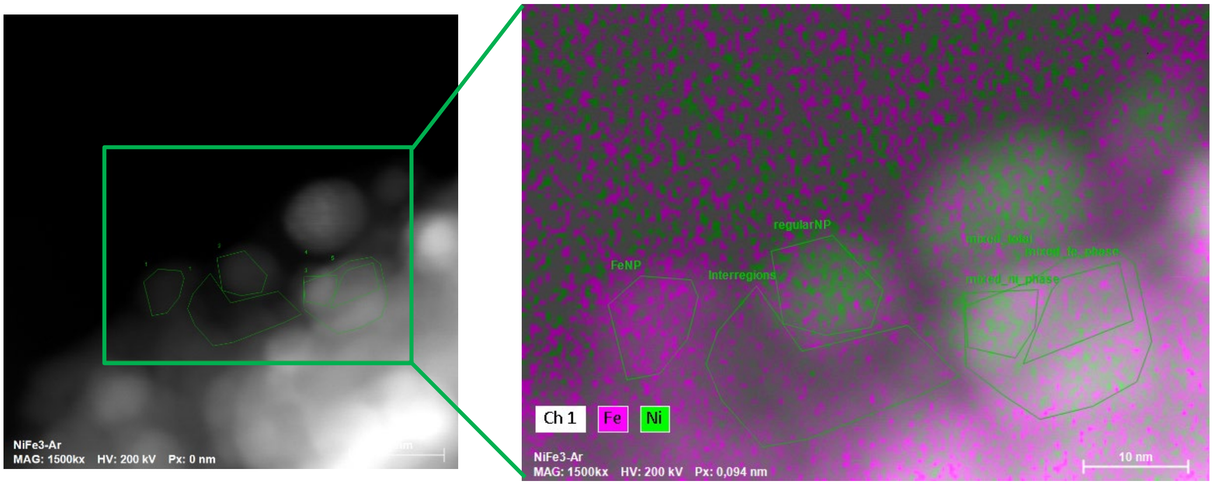

✓	EDS	Interregions	0,04	C	O 49,3	Na 2,8	Mg 2,3	Al 0,9	Si 0,1	P 12,8	S 0,1	Cl 0,1	Ca 9,1	Fe 22,0	Ni 0,5
✓	EDS	FeNP	0,04	C	O 48,6	Na 1,2	Mg 1,4	Al 0,5	Si 0,7	P 2,0	S	Cl 0,1	Ca 1,1	Fe 44,4	Ni 0,0
✓	EDS	regularNP	0,06	C	O 16,2	Na 1,1	Mg 1,1	Al 0,4	Si 0,2	P 4,8	S 0,7	Cl	Ca 3,3	Fe 42,4	Ni 29,9
✓	EDS	mixed_ni_phase	0,18	C	O 29,4	Na 2,2	Mg 1,8	Al 0,4	Si 0,5	P 7,6	S 0,2	Cl	Ca 5,4	Fe 32,3	Ni 20,1
✓	EDS	mixed_total	0,16	C	O 34,0	Na 1,9	Mg 1,4	Al 1,1	Si 0,3	P 7,2	S 0,4	Cl	Ca 5,2	Fe 33,6	Ni 14,8
✓	EDS	mixed_fe_phase	0,18	C	O 37,8	Na 2,0	Mg 1,4	Al 0,7	Si	P 8,8	S 0,3	Cl	Ca 6,6	Fe 30,6	Ni 11,9

Fig. S7 $NiFe_3$ (1:3) nanopowder after reaction in Ar atmosphere – STEM observation. STEM-EDS analyses indicate that the Fe of $NiFe_3$ nanopowder likely gets oxidized, forming $Fe_3(PO_4)_2$ with the phosphate buffer. The cleaning process of the nanoparticles after the reaction (s. Methods) strengthens the assumption that Fe and phosphate are more than just loosely associated. This is being determined via the ratios between Fe. P and O. This mapping shows that the regions between the nanoparticles almost consist exclusively of iron-phosphates precipitating from the reactions.

Heterogeneous catalysis of NMN reduction with H_2 and Ni/Fe alloys

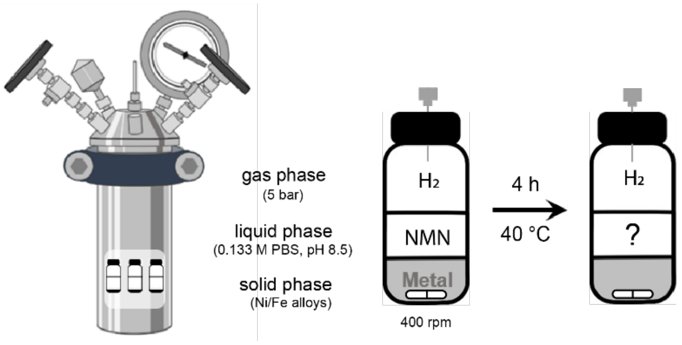

Scheme S3 The reduction of NMN with nanoparticular Ni/Fe alloys was tested with the protocol described in detail in Methods, and according to the scheme above. The amount of metal and NMN was 36 or 18 µmol, which reacted together for 4 h, at 40 °C, under alkaline conditions and 5 bars of H_2. The same reaction was made under Ar as a control.

Table S6 After 4 h under 5 bar of H_2, as shown in **Scheme S3**, samples with different nanoparticular Ni/Fe alloys yielded different amounts of 1,4-NMNH, 1,4,6-products, $NMNH_2OH$, 1,2,4,6-product and nicotinamide (Nam), from the starting material NMN, as listed below. The starting metal and cofactor were 36 or 18 µmol mixed in 3 mL of 0.133 M PBS (pH 8.5). The yields were calculated relative to the metal-free sample (100% NMN). To determine the TOF of each reaction, the sum of 1,4-NMNH, 1,4,6-products and 1,2,4,6-product was considered as the amount of product, and the total amount of metal atoms were considered as the mount of catalyst, instead of the number of molecules. Each condition was tested in duplicates and a metal-free control.

	H_2	NMN	SD	1,4-NMNH	SD	1,4,6-products	SD	$NMNH_2OH$	SD	1,2,4,6-product	SD	Nam	SD	TOF [s^{-1}]
4h	nNiFe	9.61%	6.4%	7.74%	0.5%	21.63%	3.2%	25.39%	0.6%	3.24%	0.6%	1.93%	0.3%	2.12E-05
	$nNiFe_3$	2.01%	0.1%	9.46%	0.4%	25.22%	0.7%	29.49%	0.0%	2.73%	0.4%	1.75%	0.1%	1.12E-05
	nFe^0	40.51%	5.1%	13.23%	1.0%	2.29%	0.3%	19.47%	2.1%	0.00%	0.0%	4.63%	0.2%	2.63E-05

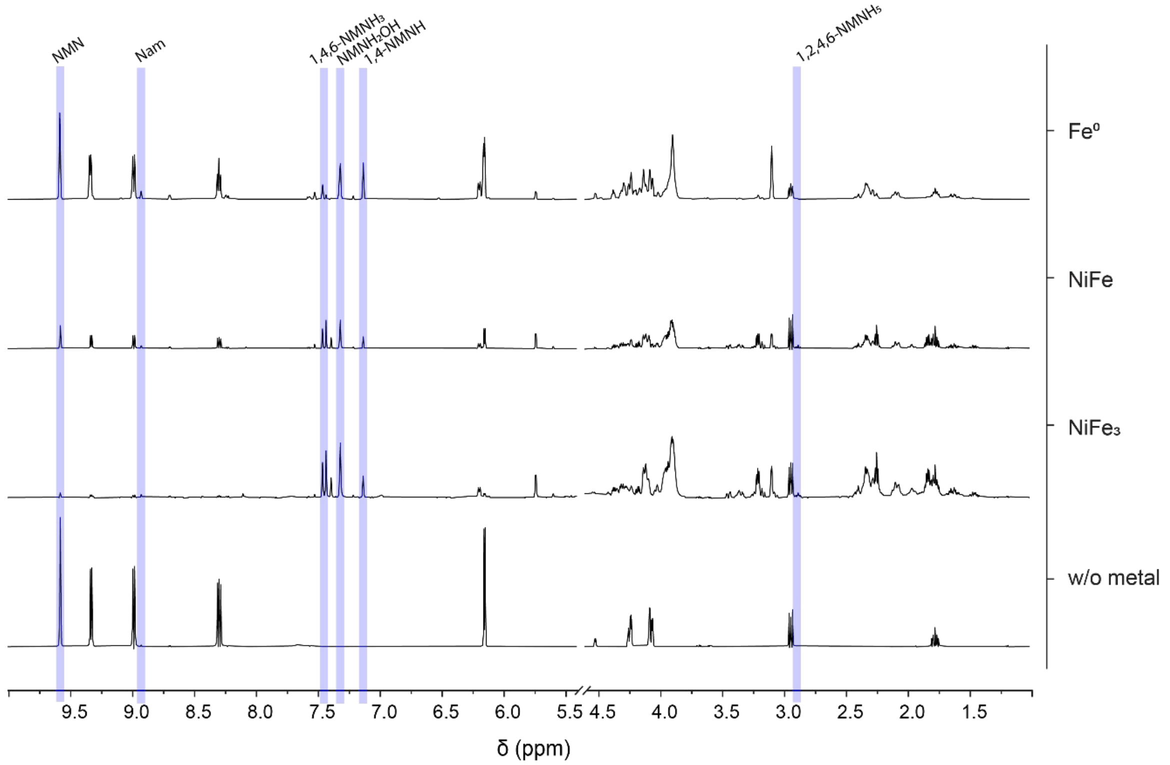

Fig. S8 The NMR spectra of replica samples of NMN in PBS (0.133 M, pH 8.5) reacted for 4h with 5 bar of H_2 and a metal 1:1 cofactor ratio, as shown in **Scheme S3**, are stacked together in this figure. The metal used in each reaction is specified on the right of each spectra. After the 4h reaction, the supernatant was collected and DSS added as an internal standard. The spectra were edited to only include relevant peaks, having been removed a DSS peak at 0 ppm and water peak at 4.8 ppm. No other peaks were found in the areas removed. Some DSS peaks are still visible (*). The peaks used for qualitative analysis and subsequent qNMR are highlighted in blue, according to **Table S8**.

Table S7 After 4 h under 5 bar of Ar, as shown in **Scheme S3** with Ar, samples with different nanoparticular Ni/Fe alloys yielded different amounts of 1,4-NMNH, 1,4,6-products, $NMNH_2OH$, 1,2,4,6-product and nicotinamide (Nam), from the starting material NMN, as listed below. The starting metal and cofactor were 36 or 18 µmol mixed in 3 mL of 0.133 M PBS (pH 8.5). The amount of metal atoms was the same as the cofactor. The yields were calculated relative to the metal-free sample (100% NMN). To determine the TOF of each reaction, the sum of 1,4-NMNH, 1,4,6-products and 1,2,4,6-product was considered as the amount of product, and the total amount of metal atoms were considered as the mount of catalyst, instead of the number of molecules.

	Ar	**NMN**	**SD**	**1,4-NMNH**	**SD**	**1,4,6-products**	**SD**	**$NMNH_2OH$**	**SD**	**1,2,4,6-product**	**SD**	**Nam**	**SD**	**TOF [s^{-1}]**
4h	nNiFe	65.07%	1.0%	4.40%	0.4%	2.92%	0.0%	7.52%	0.3%	0.00%	0.0%	1.90%	0.1%	4.75E-06
	$nNiFe_3$	54.40%	0.2%	6.72%	0.4%	3.39%	0.4%	13.31%	0.4%	0.00%	0.0%	2.38%	0.2%	6.56E-06
	nFe^0	87.37%	1.2%	0.97%	0.0%	0.00%	0.0%	0.80%	0.2%	0.00%	0.0%	5.36%	0.2%	1.33E-06

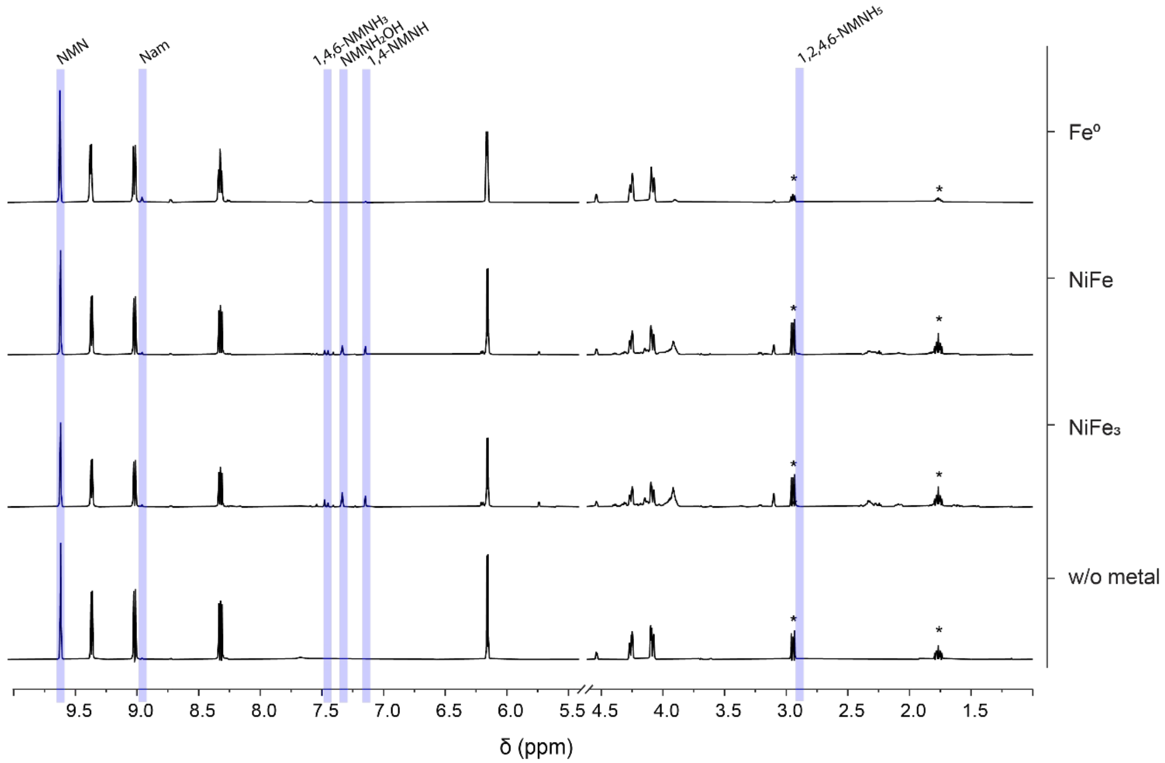

Fig. S9 The NMR spectra of replica samples of NMN in PBS (0.133 M. pH 8.5) reacted for 4h with 5 bar of Ar and a metal 1:1 cofactor ratio, as shown in **Scheme S3** with Ar, as indicated on the left side, are stacked together in this figure. The metal used in each reaction is specified on the right of each spectra. After the 4h reaction, the supernatant was collected and DSS added as an internal standard. The spectra were edited to only include relevant peaks, having been removed a DSS peak at 0 ppm and water peak at 4.8 ppm. No other peaks were found in the areas removed. Some DSS peaks are still visible (*). The peaks used for qualitative analysis and subsequent qNMR are highlighted in blue, according to **Table S8**.

Table S8 For quantification of each compound, a peak was selected in their spectra. The values listed a bellow indicate the ppm value where the peak can be found at approximately pH 8.5 (0.133M PBS).

Molecule	NMN	Nam	1,4,6-NMN	$NMNH_2OH$	1,4-NMNH	1,2,4,6-NMN
δ (ppm) for quantification	9.58	8.93	7.41/7.45	7.34	7.15	2.85

Reduction products of NMN and NAD characterized through NMR spectroscopy

Fig. S10 shows the ^{1}H spectrum of substrate **2** (numbering in order with the maintext) in 0.133 M PBS at 298 K. Characteristic signals at 9.581, 9.337, 8.990, 8.308, 6.187, 4.472 ppm were observed for H-2, H-6, H-4, H-5, H-1'and H-4' respectively. Furthermore, two double doublets at 4.197 and 4.025 ppm were detected for the diastereotopic methylene CH_2-5'.

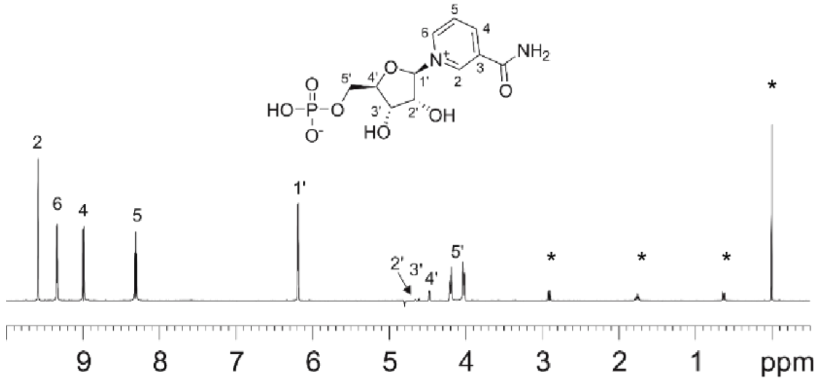

Fig. S10 ^{1}H spectrum of substrate NMN in 0.133 M PBS with the internal reference DSS (*) at 298 K(**2**).

Identification of products of substrate 2

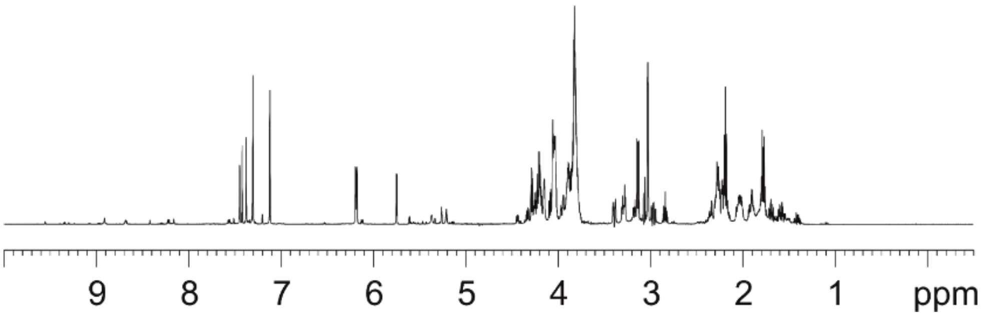

Fig. S11 ^{1}H spectrum of the products NMNH in 0.133 M PBS at 298 K.

The figure above presents ^{1}H spectrum of products solution of substrate **2** after 4 h of incubation. This spectrum shows a strong reduction in the intensity of signals from 8.3 to 9.6 ppm. which means a large degradation of the substrate. A large number of signals from 1.3 to 7.5 ppm were observed, which reveals the formation of a group of more than three different species of products. As shown in **Scheme S4**, three single reduction products, the 1,2-NMNH. 1,4-NMNH and 1,6-NMNH may form. Further reduction of the nicotineamide may follow, thus 1,2,4-, 1,2,5-, 1,2,6-, 1,4,6-, and 1,2,4,6-$NMNH_x$ may form, too.

Due to the large number of species in the reaction solution, efforts to separate the products through LC-MS turned out to be in vain. The different NMN reduced species share the same retention time with the implemented protocol. Nevertheless, with enough amount of substance in hands we

managed to use NMR spectroscopy to characterize the obtained products. It is well-known that multidimensional NMR spectroscopy plays an important role in the structure determination in synthetic chemistry, molecular biology and biochemistry and catalysis. By using two-dimensional NMR spectra, not only the funtional groups but also the connectivity among them within a molecule can be characterised. Thus, the structure of peptides and biomacromolecules can be determined[10–12].

The great challenge we were facing was to deal with a huge signal overlap caused by the mixture of various species. Fortunately, by using TOCSY signals belong to the same molecule (within one spin network) can be identified. The edited ^{1}H – ^{13}C HSQC spectra extend the scope of information, where characteric ^{13}C chemical schifts can be made used for the structure identification. Furthermore, the sign of crosspeaks in the edited HSQC spectra provides unambiguous identification of methylene and methine groups. By careful analysis of the TOCSY and edited HSQC spectra we wre able to determine the main species of those reduction products.

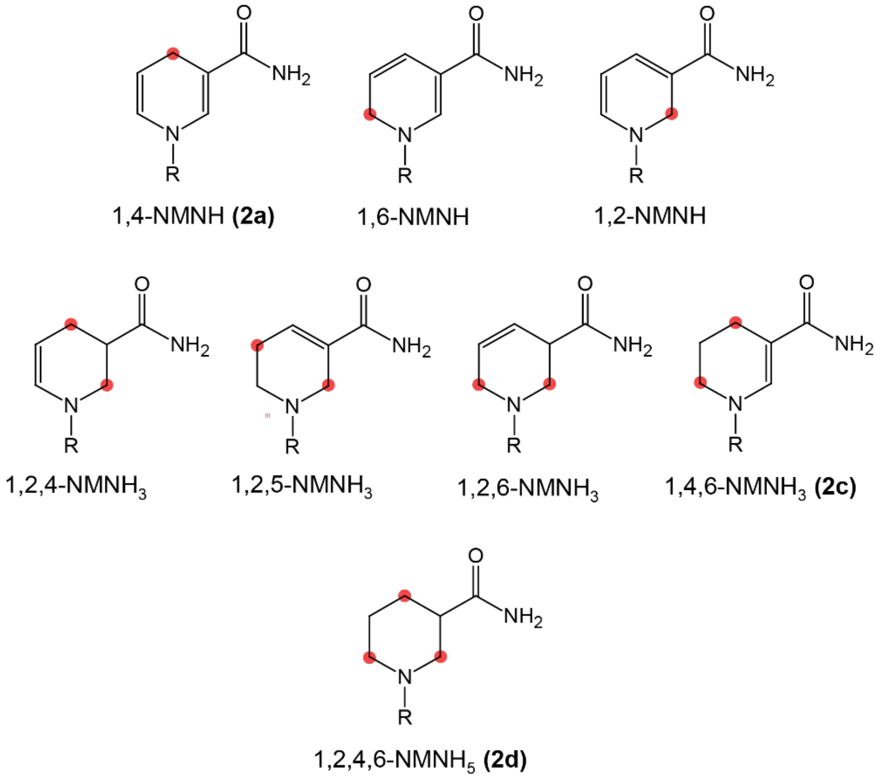

Scheme S4 Tentative reduction products of NMN reduction. Red marks the reduced carbon.

The single reduction product 2a

Starting from the well-resolved signal at 3.057 ppm, TOCSY crosspeaks (**Fig. S12**) show connectivities with signals at 7.147, 6.214, 5.774, and 5.022 ppm. The edited HSQC spectrum (**Fig. S13**) shows crosspeaks between 3.057 – 22.0 ppm for a CH_2 group, 7.147 – 138.2 ppm, 6.214 – 124.8 ppm and 5.022 – 105.3 ppm for three aromatic CH groups, and 5.774 – 91.7 ppm for a CH group at the anomeric position of a sugar. Those defined fragments correspond well to the three species of single reduction as shown in **Scheme S4**. Upon a close inspection into the ^{13}C chemical shift at 22.0 ppm, we concluded product **2a** to be 1,4-NMNH. The ^{13}C signal of the CH_2 group in both 1,2- and 1,6-NMNH (at the α-position of a tertiary amine) would appear at a much lower field (in the range 45 – 50 ppm)[13]. Since no such crosspeaks were detected, we excluded the formation of 1,2- and 1,6-NMNH from our products. Two well resolved major peaks at 7.15 ppm (d, 1.5 Hz) and 6.24 ppm (ddt, 8.1, 1.9, 1.7 Hz) were observed. In addition, a pair of very close doublets were observed at 3.056 ppm (1.6 Hz) and 3.062 ppm (1.7 Hz). Those peaks correspond well to ^1H signals at positions 2, 6 and 4 in the **Table S27**, respectively.

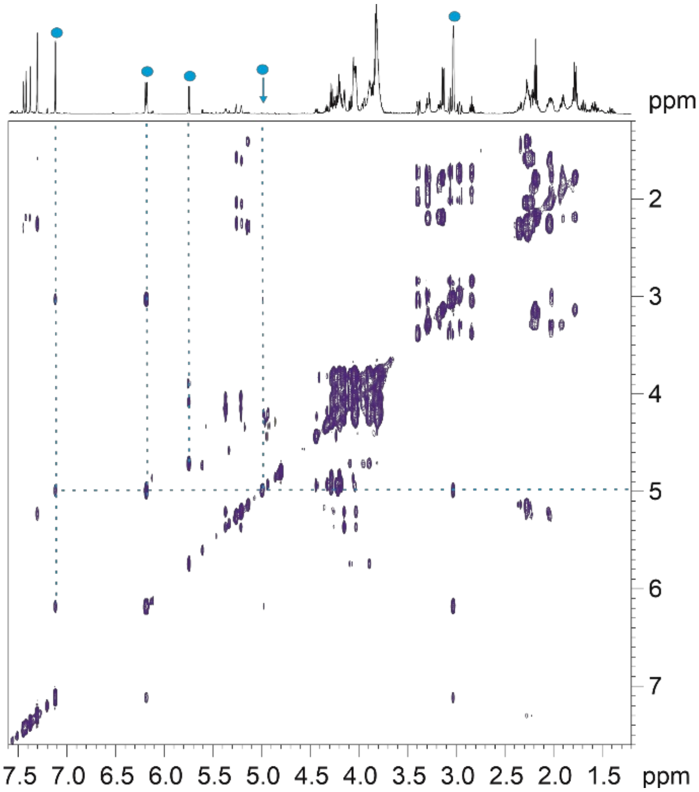

Fig. S12 ^1H-^1H TOCSY spectrum of NMN products in 0.133 M PBS at 298 K.

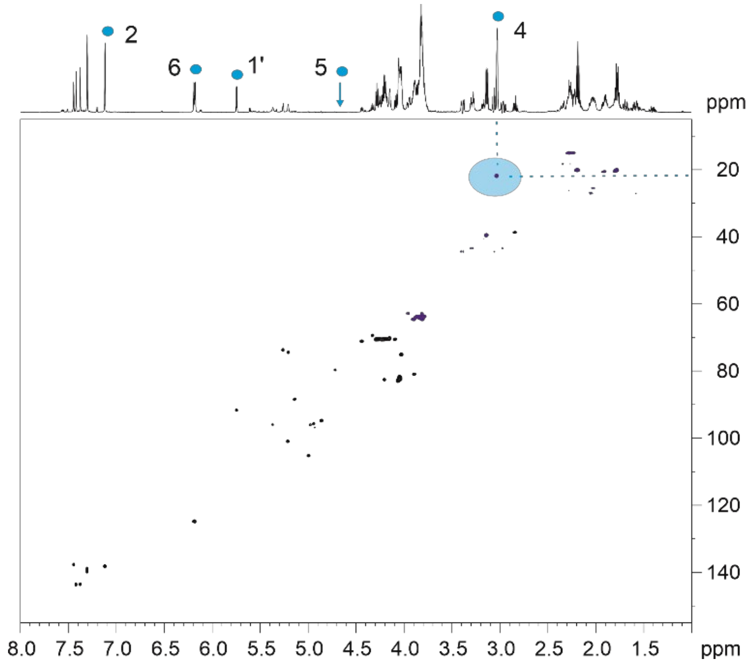

Fig. S13 The edited ^{1}H-^{13}C HSQC spectrum of products of **2** at 298 K.

Products of follow up reactions

As shown in **Scheme S4**, two of the tetrahydro-products, 1,2,4- and 1,2,6-$NMNH_3$ contain a CH group within the hetero cyclic ring. This CH group should show a characteristic crosspeak in the edited HSQC spectrum of ^{13}C chemical shift in the region 40 – 50 ppm (38), with a sign opposite to a CH_2 group. A close inspection of the spectrum shows none of such signals detected. We thus excluded these two species from our products.

Nevertheless, one crosspeak at 2.868 – 38.8 ppm was detected. Close inspection of the crosspeaks in the corresponding region of the TOCSY spectrum (**Fig. S15**) revealed a connectivity pattern for a meta-substituted piperidine. Therefore, a hexahydro product **2d** was identified to have the same ring structure as 1,2,4,6-$NMNH_5$. The highlighted peak is for the methine CH and the ^1H signal at 2.87 ppm stands for proton at carbon 3 (**Fig. S14**). Given that the remaining structure is unclear, **2d** will be referenced as 1,2,4,6-product.

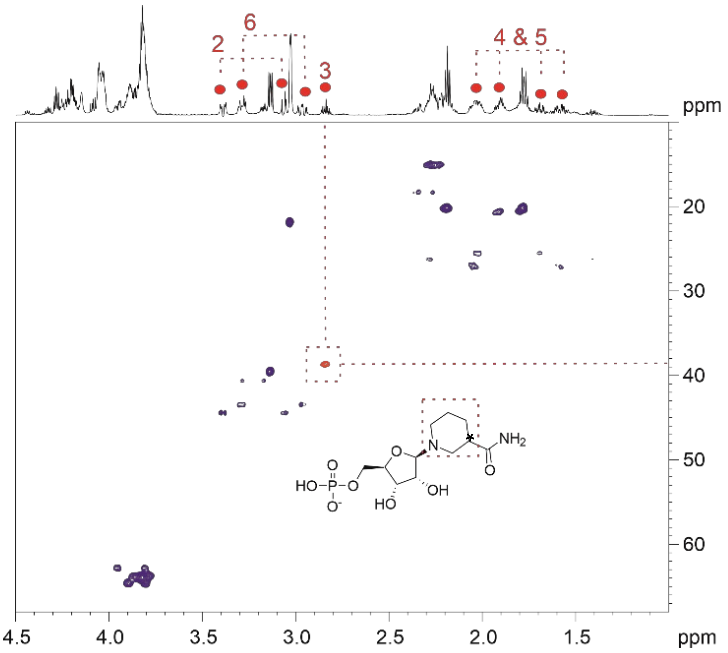

Fig. S14. Section of edited ^1H-^{13}C HSQC spectrum of products of **2** at 298 K, methine peak of carbon 3 shown via asterisk.

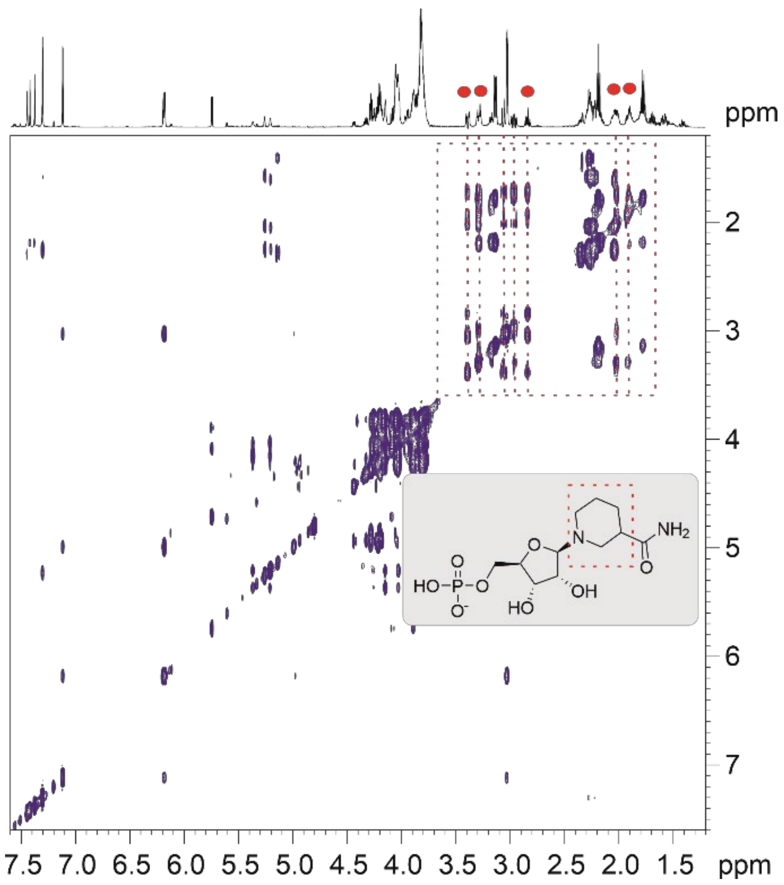

Fig. S15 Section of ^{1}H-^{1}H TOCSY spectrum of products of **2** at 298 K.

Similarly, crosspeaks at 7.448/7.405 – 143.7 (not shown **Fig. S16**), 3.165 – 39.7, 2.215 – 20.3, and 1.808 – 20.3 ppm were detected in the edited HSQC spectrum (**Fig. S16**). Relatively resolved ^{1}H signals at 3.165 ppm (dd, 6.5, 5.0 Hz), 2.215 ppm (t, 6.7 Hz) and 1.808 ppm (m) were observed and could be assigned to three methylene groups, respectively. Connectivities among peaks at 7.448 , 7.405 ppm with peaks at 3.165, 2.215 and 1.808 ppm were observed in TOCSY spectrum (**Fig. S16**). The tetrahydro-product **2c** was identified to have the same ring structure as 1,4,6-NMNH3. The observation of two peaks at 7.448 ppm (s) and 7.405 ppm (s) for H-2 can be attributed to different isomers of 1,4,6-NMNH3, or degradation products of 1,4,6-NMNH3, so they will be generally referenced as 1,4,6-products[14,15].

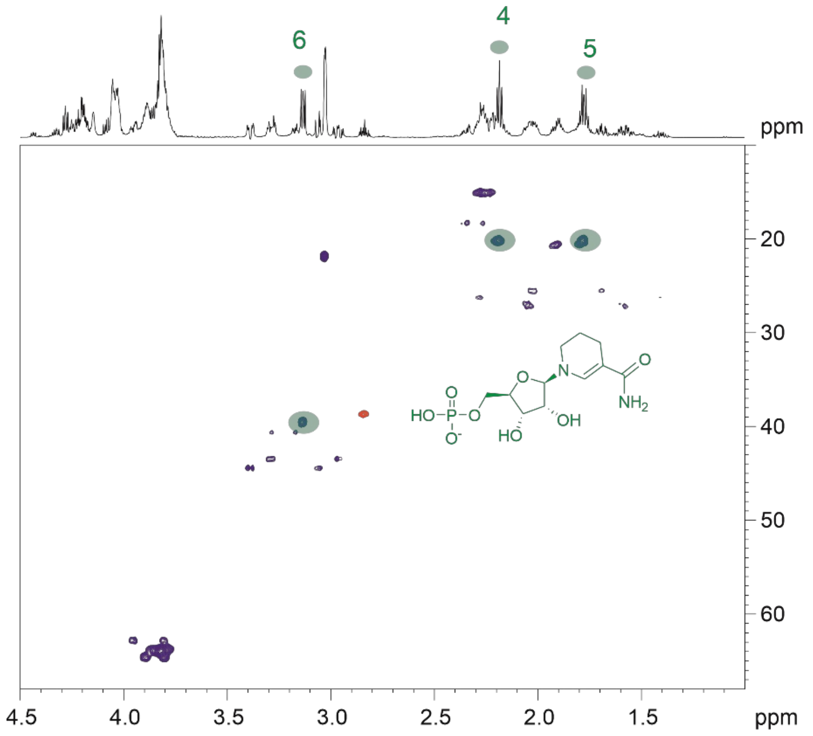

Fig. S16 Section of edited ^{1}H-^{13}C HSQC spectrum of products of **2** at 298 K.

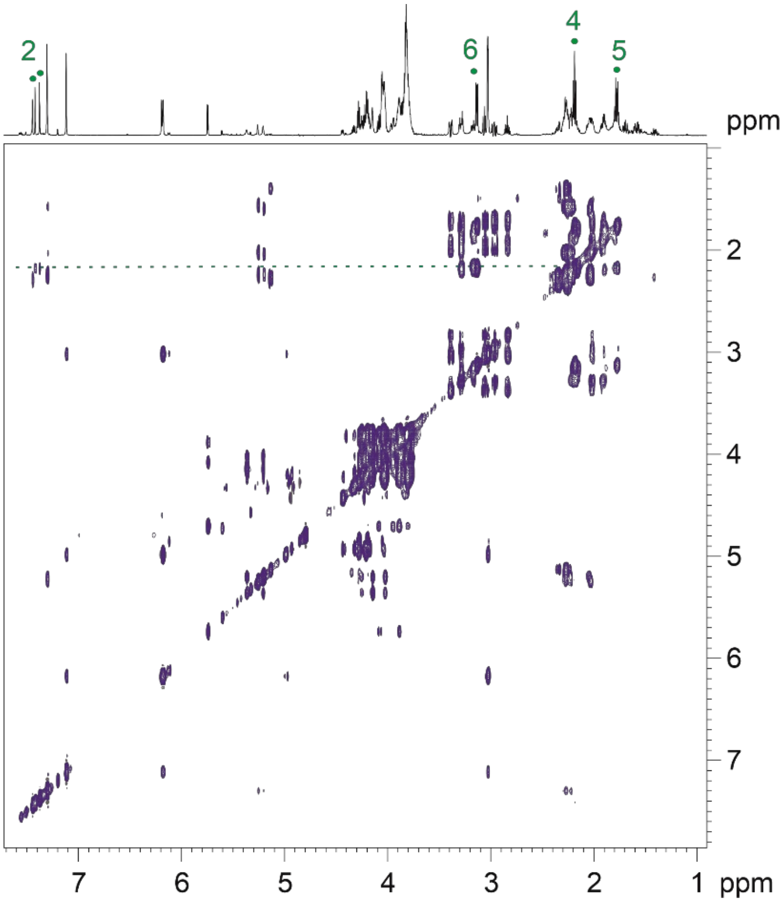

Fig. S17 Section of ^{1}H-^{1}H TOCSY spectrum of products of **2** at 298 K.

In summary, in the reaction mixture of substrate **2.** we were able to identify product **2a** (1,4-NMNH), product **2d** (1,2,4,6-product), and product **2c** (1,4,6-products).

Identification of hydration product $NMNH_2OH$

In order to identify the peak at 7.35 ppm in the ^1H spectrum we put further efforts. Via LC-MS a hydration product with molecular formula $C_{11}H_{18}N_2O_9P^-$ and 353.07323 m/z (mass accuracy of 5 ppm) was revealed (**Fig. S32**). Following the first reduction step, hydration upon 1,4-NMNH took place and products 1,4-$NMNH_2OH$ formed. Thus, crosspeaks at 7.35 – 138.9. 7.35 – 139.9. 5.29 – 73.9, 5.24 – 74,6, 2.25/2.31 – 15.3 and 2.07/1.06 – 27.1 ppm were detected in the edited HSQC spectrum (**Fig. S19**). Together with connectivity in TOCSY spectrum (**Fig. S18**) hydration products as revealed by LC-MS were verified.

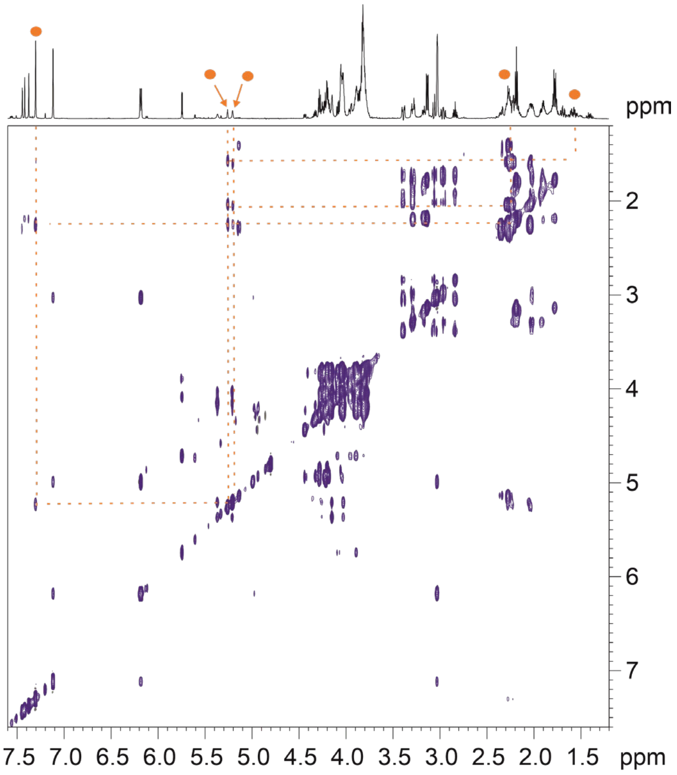

Fig. S18 Section of ^1H-^1H TOCSY spectrum of products of 1 at 298 K, with highlight for the hydration products.

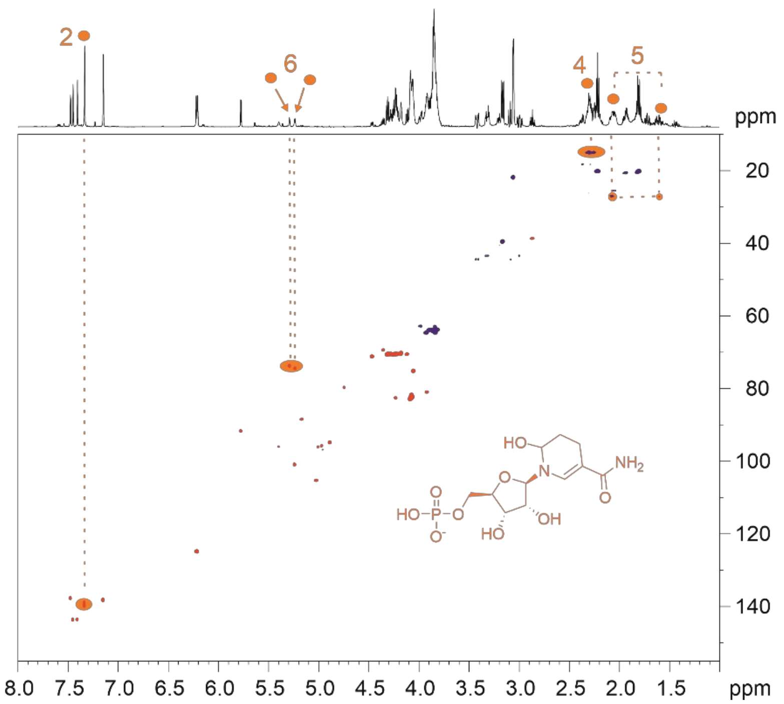

Fig. S19 Section of edited ^{1}H-^{13}C HSQC spectrum of products of 1 at 298 K. with highlight for the hydration products.

In summary, in the reaction mixture of substrate **2** we were able to identify product **2a** (1,4-NMNH), product **2d** (1,2,4,6-product), products **2c** (1,4,6-products) and verify the hydration product 1,4 ($NMNH_2OH$; **2b**).

Overtime reduction of NAD/NMN with H_2 and $NiFe_3$

For quantification of each product, the ppm values of **Table S1**, and **Table S8** should be considered.

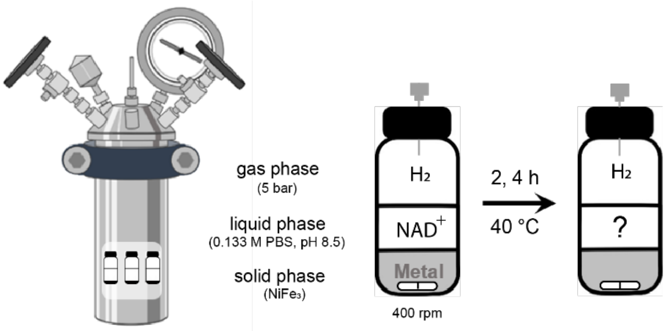

Scheme S5 The reduction of NAD^+ with $nNiFe_3$ alloys was tested with the protocol described in detail in Methods, and according to the scheme above. The amount of metal and NAD^+ was 36 µmol, which reacted together for 2 and 4 h, at 40 °C, under alkaline conditions and 5 bars of H_2. The same reaction was made under Ar as a control.

Table S9 After 2 and 4 h under 5 bar of H_2, as shown in **Scheme S5**, samples with $NiFe_3$ yielded different amounts of 1,4-NADH, 1,6-NADH, and Nam, from the starting material NAD^+. The starting metal and cofactor were 36 µmol mixed in 3 mL of 0.133 M PBS (pH 8.5). The amount of metal atoms was the same as the cofactor. The yields were calculated relative to the metal-free sample (100% NAD^+). To determine the TOF of each reaction, the sum of 1,4-NADH, and 1,6-NADH was considered as the amount of product, and the total amount of metal atoms was considered as the mount of catalyst, instead of the number of molecules. Experiments of 2h had duplicates while 4h long experiments had four replicas.

	H_2	**NAD^+**	**SD**	**1,4-NADH**	**SD**	**1,6-NADH**	**SD**	**Nam**	**SD**	**TOF [s^{-1}]**
4h	$nNiFe_3$	25.59%	4.4%	40.60%	1.8%	16.70%	0.6%	4.48%	0.1%	3.02E-05
2h		68.38%	2.5%	15.05%	1.3%	6.61%	0.6%	7.41%	0.4%	3.18E-05

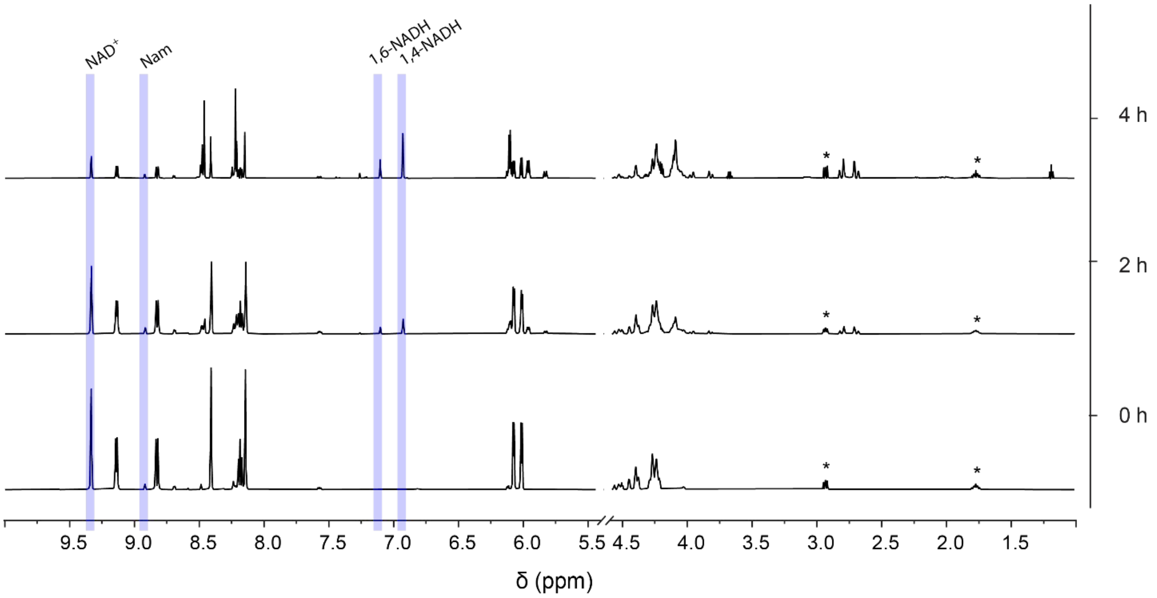

Fig. S20 The NMR spectra of replica samples of 36 µmol NAD^+ in PBS (0.133 M, pH 8.5) with 5 bar of H_2 and 36 µmol $NiFe_3$ (1:1 cofactor ratio), as shown in **Scheme S5**, are stacked together in this figure. After 0, 2 and 4 h, the supernatant was collected and DSS added as an internal standard. The spectra were edited to only include relevant peaks, having been removed a DSS peak at 0 ppm and water peak at 4.8 ppm. No other peaks were found in the areas removed. Some DSS peaks are still visible (*). The peaks used for qualitative analysis and subsequent qNMR are highlighted in blue, according to **Table S1**.

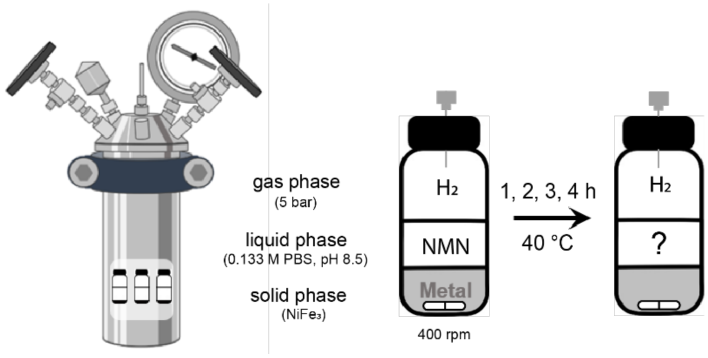

Scheme S6 The reduction of NMN with $NiFe_3$ was tested with the protocol described in detail in **Methods**, and according to the scheme above. The amount of metal and NMN was 36 or 18 µmol, which reacted together for 1, 2, 3, and 4 h, at 40 °C, under alkaline conditions and 5 bars of H_2. The same reaction was made under Ar as a control.

The reactions that ran for 1, 2 and 3 h had a smaller, overlapping peak at 7.35 ppm, adjacent to $NMNH_2OH$'s peak. This could not be assigned beyond a doubt, so the peak's area was subtracted by using a deconvolution tool (Mestrenova v.15.0.1). The contribution to $NMNH_2OH$'s peak area reaches 1% at the 3h measurement. In all 4h experiments in this paper we could not observe this additional peak and did thus not account for its presence.

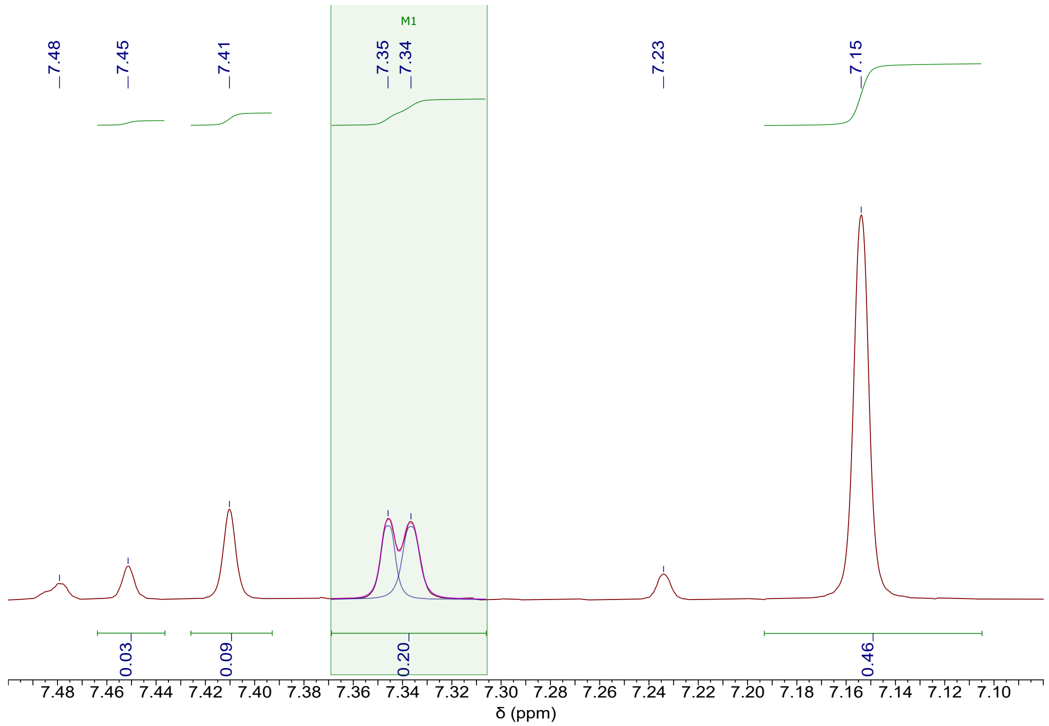

Fig. S21 Deconvolution of overlapping peaks at 7.35 and 7.34 ppm from an NMN sample that reacted for 1h with $NiFe_3$ as described in **Scheme S6**. The deconvolution was done using the line fitting tool of MestReNova (v.15.0.1) and applied according to **Table S10**.

Table S10 After 1 h under 5 bar of Ar. as shown in **Scheme S6**. Samples with $NiFe_3$ showed two overlapping peaks at 7.35 (L) and 7.34 (R) ppm, which required deconvolution. The starting metal and cofactor were 36 µmol mixed in 3 mL of 0.133 M PBS (pH 8.5). The amount of metal atoms was the same as the cofactor. The concentration had been previously calculated with the total area of the peaks. To correct that, the ratio of $NMNH_2OH$ was calculated (R area / Total area) and then multiplied to the previous value, resulting in the "Final" amount of this compound in each sample.

	Previous (mM)	L peak area	R peak area	Ratio	Final (mM)
Sample 1	1.813	3831991.71	4269846.71	0.527021953	0.955
Sample 2	1.954	3758847.17	4516297.98	0.545766618	1.066
Sample 3	1.163	2870004.23	2050432.66	0.416717602	0.485

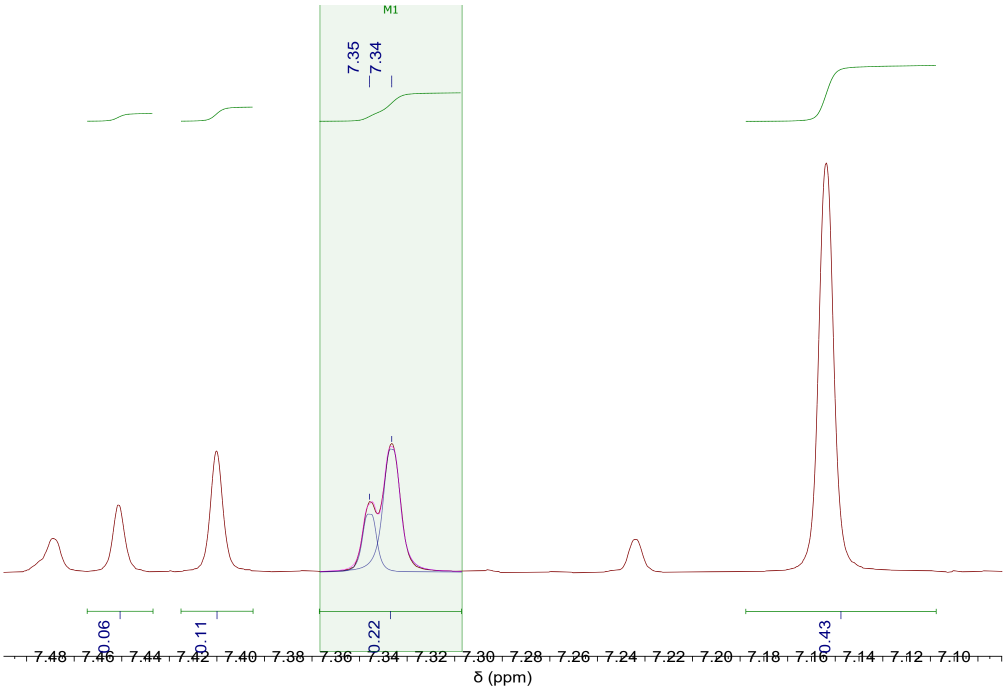

Fig. S22 Deconvolution of overlapping peaks at 7.35 and 7.34 ppm from an NMN sample that reacted for 2h with $NiFe_3$ as described in **Scheme S6**. The deconvolution was done using the line fitting tool of MestReNova (v.15.0.1) and applied according to **Table S11**.

Table S11 After 2 h under 5 bar of Ar. as shown in **Scheme S6**. Samples with $NiFe_3$ showed two overlapping peaks at 7.35 (L) and 7.34 (R) ppm, which required deconvolution. The starting metal and cofactor were 36 μmol mixed in 3 mL of 0.133 M PBS (pH 8.5). The amount of metal atoms was the same as the cofactor. The concentration had been previously calculated with the total area of the peaks. To correct that, the ratio of $NMNH_2OH$ was calculated (R area / Total area) and then multiplied to the previous value, resulting in the "Final" amount of this compound in each sample.

	Previous (mM)	L peak area	R peak area	Ratio	Final (mM)
Sample 1	2.195	2498514.91	6901725.62	0.734207343	1.612
Sample 2	2.124	1868116.34	7163206.11	0.793151407	1.684

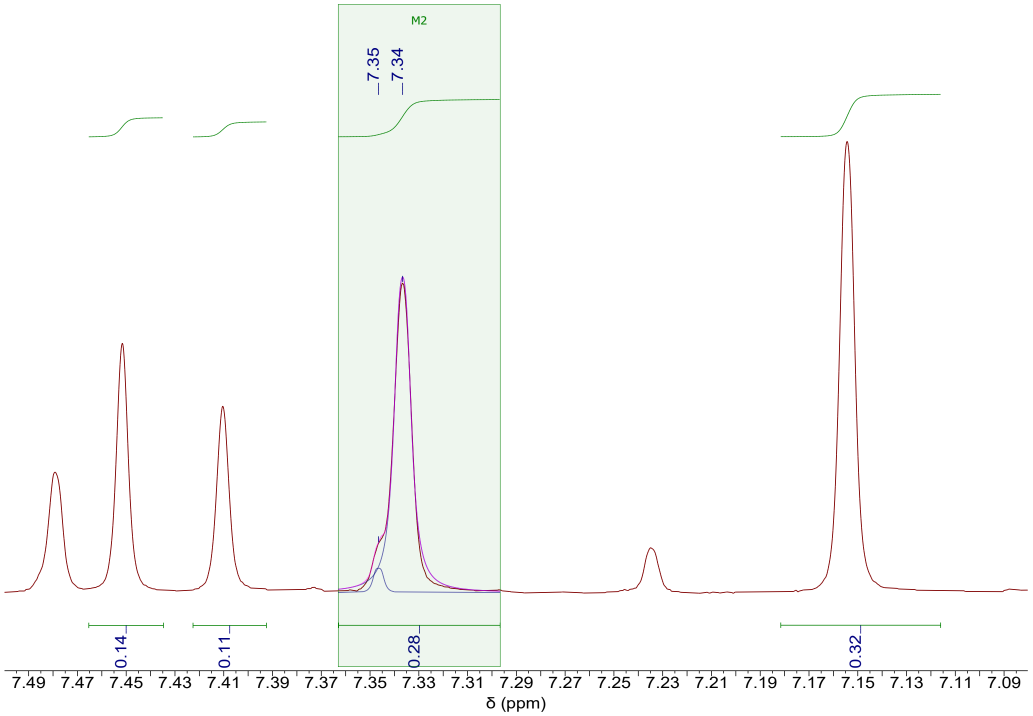

Fig. S23 Deconvolution of overlapping peaks at 7.35 and 7.34 ppm from an NMN sample that reacted for 3h with $NiFe_3$ as described in **Scheme S6**. The deconvolution was done using the line fitting tool of MestReNova (v.15.0.1) and applied according to **Table S12**.

Table S12 After 3 h under 5 bar of Ar. as shown in **Scheme S6**. Samples with $NiFe_3$ showed two overlapping peaks at 7.35 (L) and 7.34 (R) ppm, which required deconvolution. The starting metal and cofactor were 36 μmol mixed in 3 mL of 0.133 M PBS (pH 8.5). The amount of metal atoms was the same as the cofactor. The concentration had been previously calculated with the total area of the peaks. To correct that, the ratio of $NMNH_2OH$ was calculated (R area / Total area) and then multiplied to the previous value, resulting in the "Final" amount of this compound in each sample.

	Previous (mM)	L peak area	R peak area	Ratio	Final (mM)
Sample 1	2.958	1206602.89	12249322.75	0.910329254	2.693
Sample 2	2.517	0	10142853.63	1	2.517
Sample 3	2.825	509801.41	12292243.48	0.960178127	2.712

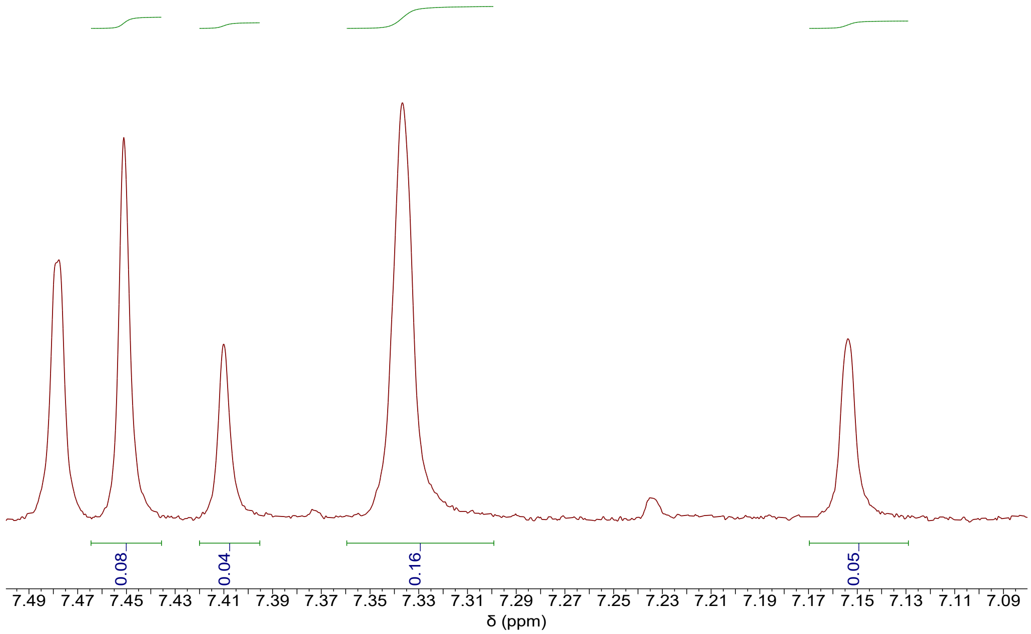

Fig. S24 ^{1}H-NMR spectra of a samples from the starting material NMN, that reacted for 4h with $NiFe_3$ as described in **Scheme S6**. Samples like this, did not appear to have any overlapping peaks with the $NMNH_2OH$ peak at 7.34 ppm, and thus deconvolution was not needed.

Overtime reduction of NAD/NMN with H_2 and $NiFe_3$

Table S13 After 1, 2, 3, and 4 h under 5 bar of H_2, as shown in **Scheme S6**, samples with $NiFe_3$ yielded different amounts of 1,4-NMNH, $NMNH_2OH$, 1,4,6-products, 1,2,4,6-product, and nicotinamide (Nam), from the starting material NMN. The starting metal and cofactor were 36 µmol mixed in 3 mL of 0.133 M PBS (pH 8.5). The amount of metal atoms was the same as the cofactor. The yields were calculated relative to the metal-free sample (100% NMN). To determine the TOF of each reaction, the sum of 1,4-NMNH, 1,4,6-products, 1,2,4,6-product was considered as the amount of product, and the total amount of metal atoms was considered as the mount of catalyst, instead of the number of molecules. 2h and 4h were tested with duplicates while 1h and 3h with triplicates.

	H_2	NMN	SD	1,4-NMNH	SD	1,4,6-products	SD	$NMNH_2OH$	SD	1,2,4,6-product	SD	Nam	SD	TOF [s^{-1}]
4h	$nNiFe_3$	2.01%	0.1%	9.46%	0.4%	25.22%	0.7%	29.49%	0.0%	2.73%	0.4%	1.75%	0.1%	1.12E-05
3h		1.21%	0.6%	24.74%	0.0%	19.72%	2.6%	21.32%	1.4%	2.82%	0.6%	2.05%	0.2%	7.10E-05
2h		9.38%	3.5%	34.03%	0.7%	14.48%	1.4%	16.64%	0.3%	1.78%	0.4%	2.54%	0.2%	7.55E-05
1h		30.80%	16.7%	29.84%	7.1%	7.40%	2.7%	12.67%	2.7%	0.73%	0.5%	2.87%	0.3%	1.14E-04

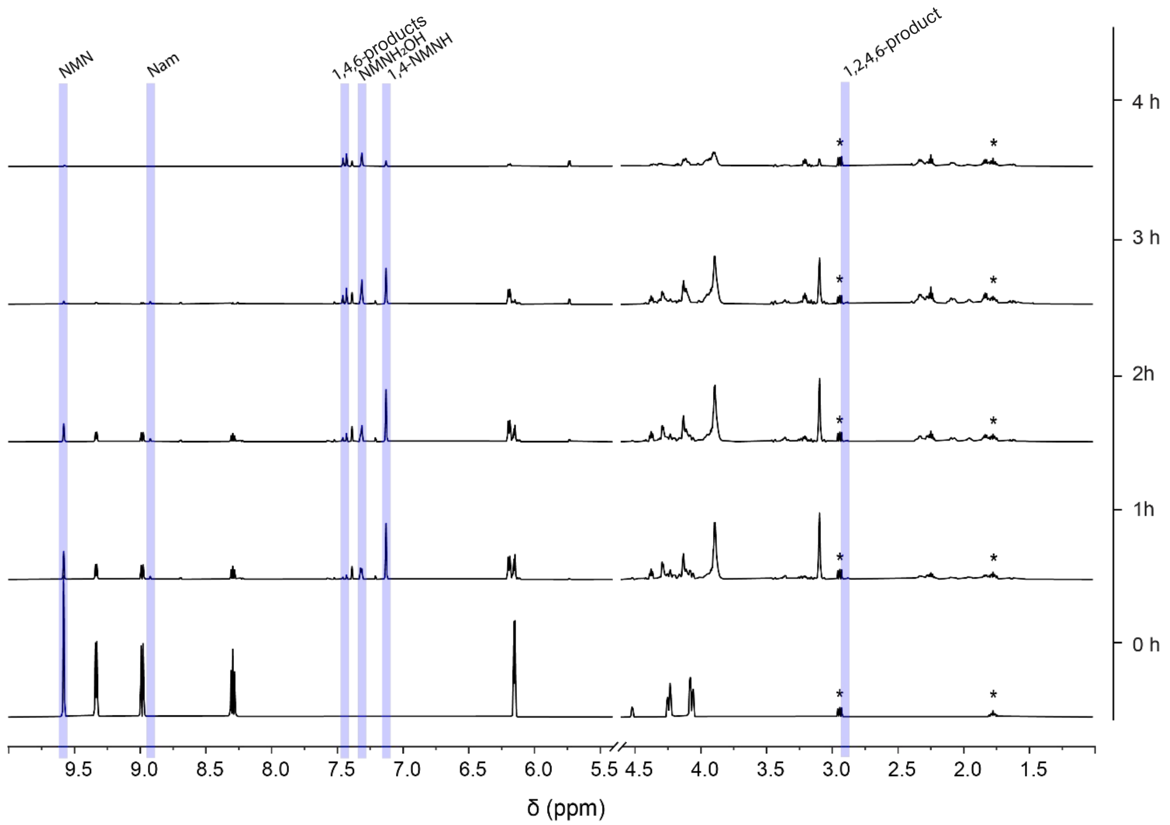

Fig. S25 The NMR spectra of replica samples of 36 or 18 μmol NMN in PBS (0.133 M, pH 8.5) with 5 bar of H_2 and equimolar amounts of $nNiFe_3$ (1:1 ratio), as shown in **Scheme S6**, are stacked together in this figure. After 0, 1, 2, 3 and 4 h, the supernatant was collected and DSS added as an internal standard. The spectra were edited to only include relevant peaks, having been removed a DSS peak at 0 ppm and water peak at 4.8 ppm. No other peaks were found in the areas removed. Some DSS peaks are still visible (*). The peaks used for qualitative analysis and subsequent qNMR are highlighted in blue, according to **Table S8**.

Identification of products of substrate 1

Fig. S26 shows the ^{1}H spectrum of substrate **1** in 0.133 M PBS at 298 K. Characteristic signals at 9.339, 9.156, 8.825, 8.198 ppm were observed for H-2. H-6. H-4 and H-5 of the nicotinamide unit, signals at 8.379 and 8.019 ppm were detected for H-8 and H-2 of the adenine unit, while two signals at 6.097 and 5.993 ppm for H-1'of nicotinamide and adenine. Respectively, negligible impurities may exist.

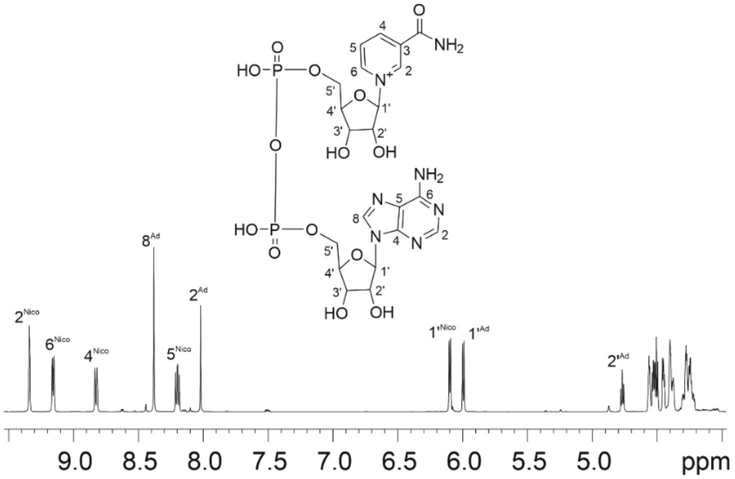

Fig. S26 ^{1}H spectrum of substrate NAD^+ in 0.133 M PBS at 298 K(**2**).

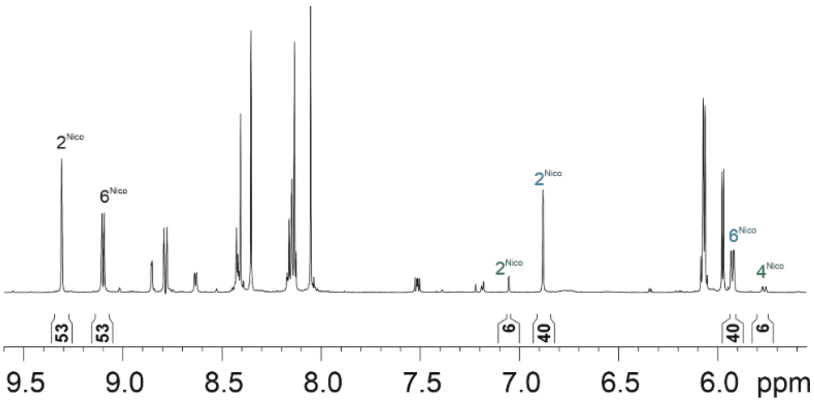

Fig. S27 ^{1}H spectrum in the region 5.6 – 9.6 ppm of products of substrate **1** at 298 K. The labels show signal assignment (cyan for the major and green for the minor product) and the integral values show the conversion rates.

The ^{1}H spectrum in the region 5.6 – 9.6 ppm of the reaction solution of substrate **1** after 4 hours of incubation in 0.133 M PBS is shown in **Fig. S27**. Different to the situation of substrate **2,** remaining of the substrate **1** was observed. One major and one minor product could be detected. Signal integral revealed a conversion rate of 40% and 6% for the major and the minor product. respectively. Further products of a scale comparable to impurities may form too.

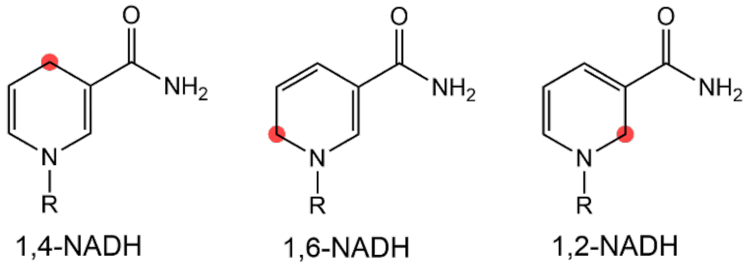

Scheme S7 Tentative reduction products of substrate **1**.

Scheme S7 shows the possible products of a single reduction of substrate **1**. Section of the TOCSY spectrum is shown in **Fig. S28**. To start, the double doublet characteristic of a methylene at 2.647 and 2.616 ppm. crosspeaks with signals at 6.903, 5.946, and 4.725 ppm were detected. As shown in **Fig. S28**, these signals correspond to the main product. In the edited HSQC spectrum (**Fig. S29**) crosspeaks 2.647 – 21.8 and 2.616 – 21.8 ppm were observed. This ^{13}C chemical shift corresponds to the C_{N-4} (22.3 ppm) of 1,4-NADH[9]. Similarly, a minor double doublet at 3.913 and 3.776 ppm was observed, whose crosspeaks with signals at 7.075. 5.785 and 5.000 ppm were detected. These signals were assigned to the second product. A close inspection of the edited HSQC spectrum revealed crosspeaks at 3.913 – 41.6 and 3.776 – 41.6 ppm. This ^{13}C chemical shift corresponds to the C_{N-6} (42.1 ppm) published for 1,6-NADH[16]. We thus concluded 1,4-NADH and 1,6-NADH to be major and minor products of substrate **1**.

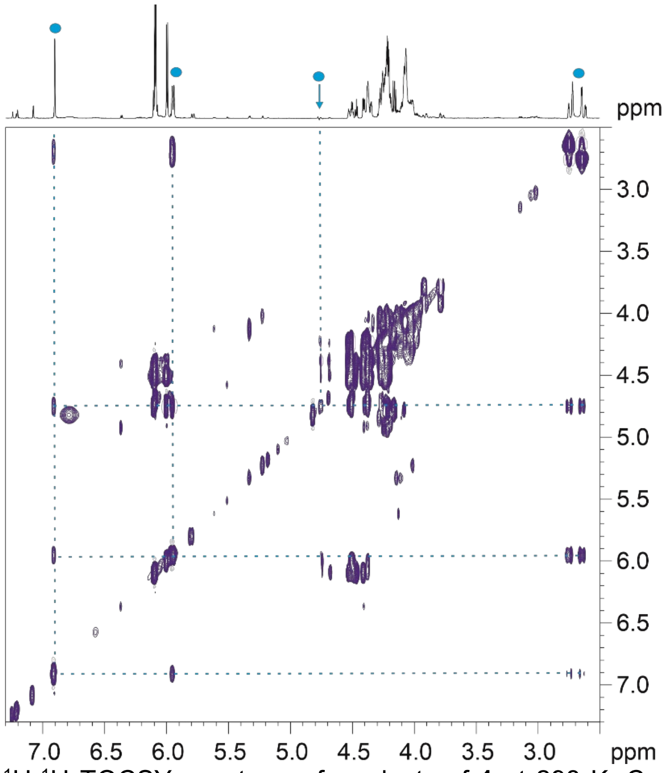

Fig. S28 Section of ^{1}H-^{1}H TOCSY spectrum of products of **1** at 298 K. Connectivity among the signals of the major product is highlighted.

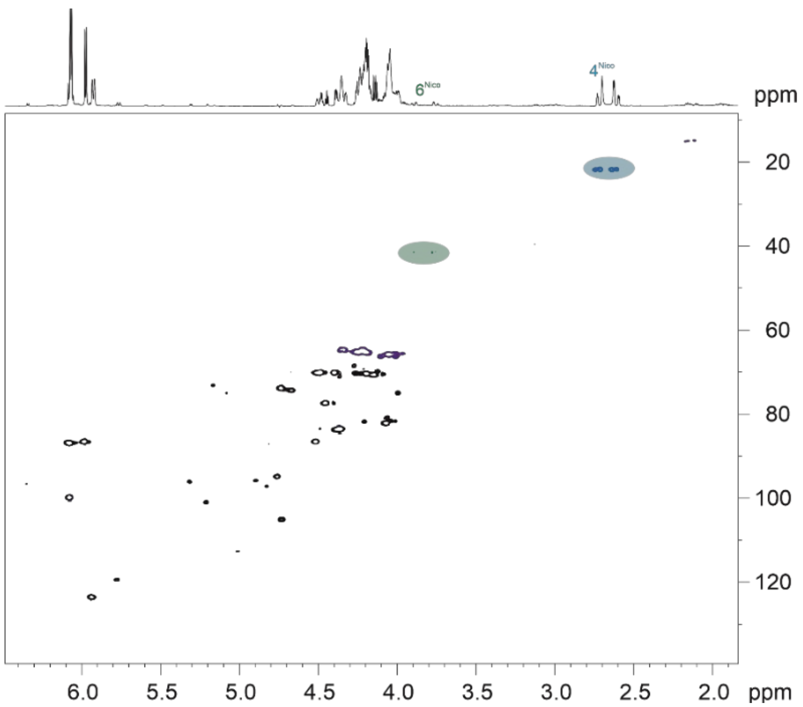

Fig. S29 Section of edited ^{1}H-^{13}C HSQC spectrum of products of **1** at 298 K.

Characterization of the 2nd reaction product with Fe^0 and LC-MS (7.35 ppm in ^{1}H-NMR)

In order to identify the second product of NMN reduction with H_2 and Fe^0, sample from such reaction (NMN 18 µmol, nanopowder Fe^0 18 µmol, 4 h. 40 °C. 5 bar H_2) was subjected to 2D-NMR and LC-MS qualitative analysis.

LC-MS analysis is in agreement that the main product is 1,4-NMNH. Three different molecules seem to be detected through chromatography, and one matches the retention time of the 1,4-NMN standard. It is possible that we have different conformations of this product in our samples. Its absence in the control confirms it to be a product obtained during the described reaction.

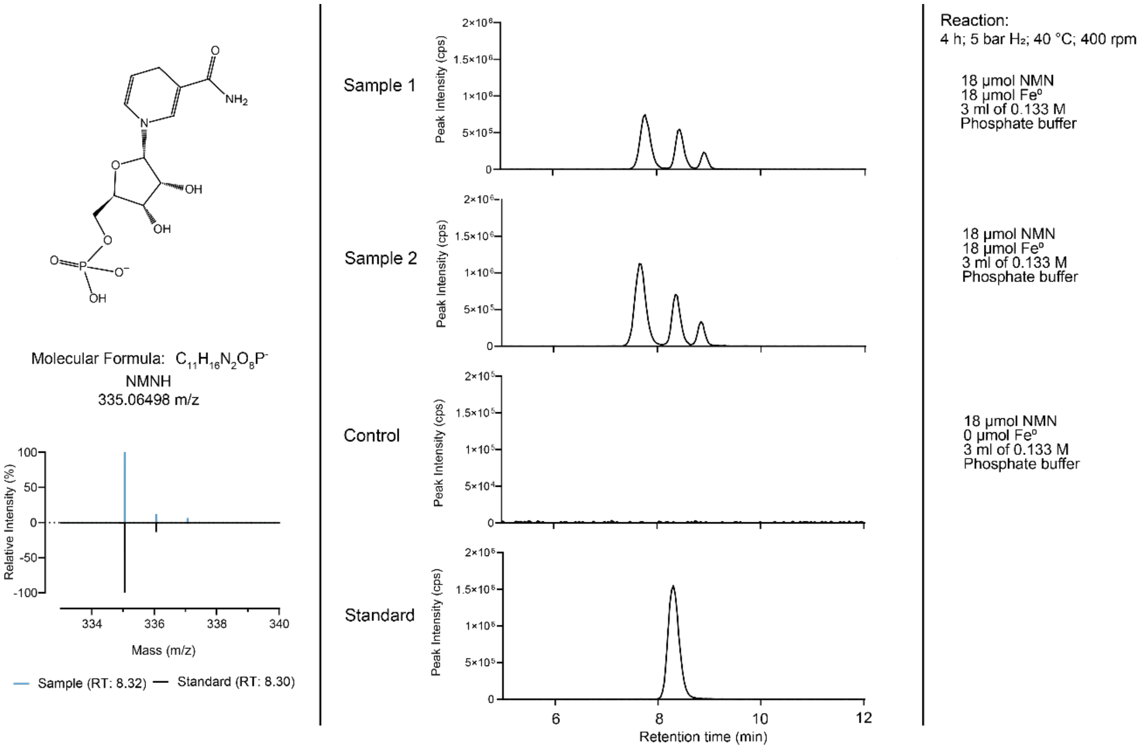

Fig. S30 The Extracted Ion Chromatogram (EIC) of mass 335.06368 (mass accuracy of 5 ppm) against the Retention Time (RT) reveals molecules of the mass 335.0649 m/z in the samples from the reaction described on the right. Samples were diluted 1:20 with waster after the reaction for measurement. The natural isotope distribution of the 1,4-NMNH standard (335.06498 m/z) matches that of the samples.

All Diels Alder products suggested in **Scheme S8** share the same mass and charge, which is also the same mass-to-charge of NMNH. Even though molecules of mass 335.06498, were revealed in the chromatogram, analysis of the isotope distribution revealed no doubled charged molecules, thus excluding Diels Alder reactions as products of the reaction with Fe^0.

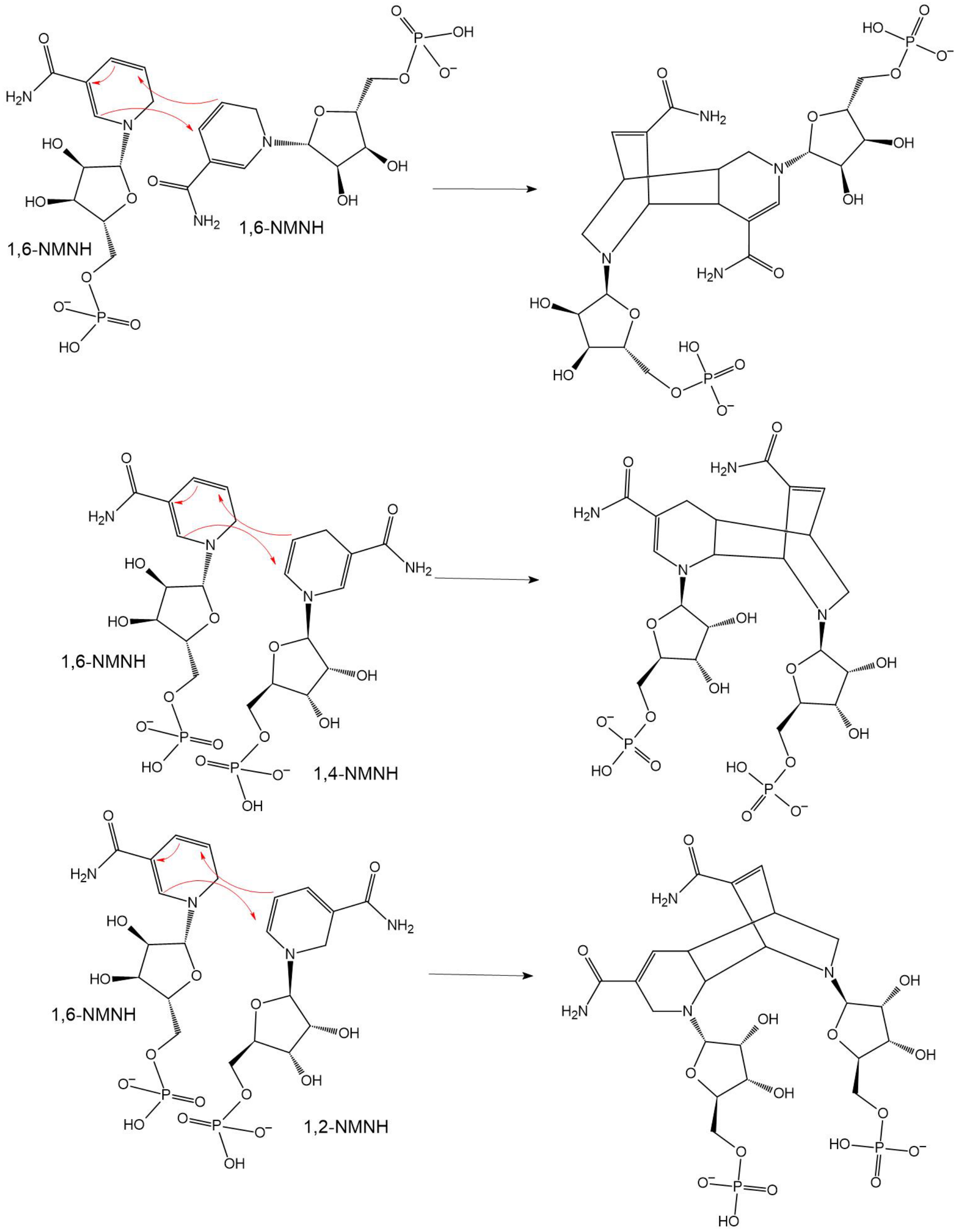

Scheme S8 Possible Diels-Alder reactions to consume 1,4-NMNH

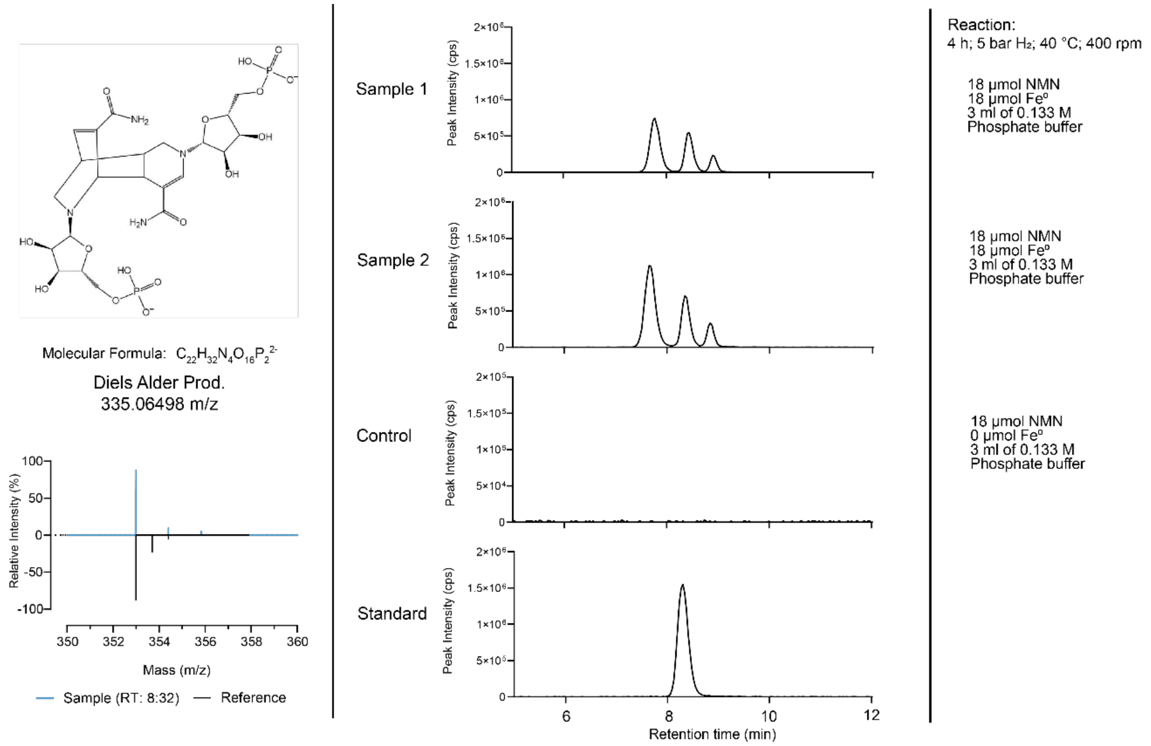

Fig. S31 The Extracted Ion Chromatogram (EIC) of mass 335.06368 (mass accuracy of 5 ppm) against the Retention Time (RT) reveals molecules of the mass 335.0649 m/z in the samples from the reaction described on the right. Samples were diluted 1:20 with waster after the reaction for measurement. The standard was prepared in water at similar final concentrations. The theoretical natural isotope distribution of the proposed Diels Alder products (335.06498 m/z), extracted from the website ChemCalc, is compared to that of the samples.

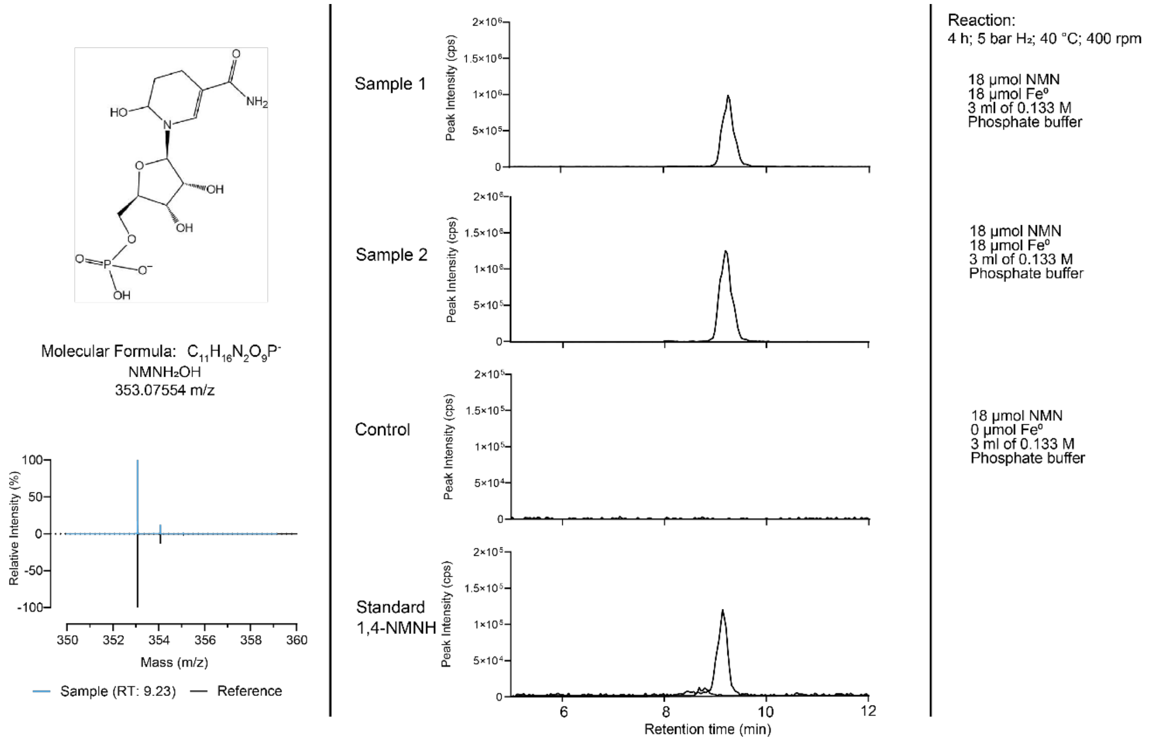

Fig. S32 The Extracted Ion Chromatogram (EIC) of mass 353.07323 (mass accuracy of 5 ppm) against the Retention Time (RT) reveals molecules of the mass 353.07323 m/z in the samples from the reaction described on the right. Samples were diluted 1:20 with waster after the reaction for measurement. The standard was prepared in water at similar final concentrations. The theoretical natural isotope distribution of $NMNH_2OH$ (353.07554 m/z), extracted from the website ChemCalc, is compared to that of the samples.

Further analysis of the samples revealed high amounts of a molecule of mass 353.07554 in the samples, which were not in the control. The mass and the isotope distribution of the sample matches that of $NMNH_2OH$. The same signal was detected in the 1,4-NMNH standard, however, the relative abundance to the standard itself, shows it is likely a small contamination from the synthesis of the standard or a consequence of sample preparation and injection into the LC-MS column. The relative abundance of this molecule to 1,4-NMNH, in the samples, is much more significant, being likely a product of the Fe^0 reaction. Its abundance suggests it could be the molecule detected at 7.35 ppm (^{1}H-NMR). 2D-NMR analysis confirmed the proposed structure to be of $NMNH_2OH$ (**Supplementary Data Fig. S9** and **S10**)

Reduction of NMN with μNi^0 and μFe^0 (1:200)

Table S14 After 4 h under 5 bar of H_2, as shown in **Scheme S14**, samples with μNi^0 or μFe^0 yielded different amounts of 1,4-NMNH, 1,4,6-products, 1,2,4,6-product, NMNH$_2$OH, and nicotinamide (Nam), from the starting material NMN. The starting metal and cofactor were 3.2 mmol and 18 μmol, respectively, mixed in 3 mL of 0.5 M PBS (pH 8.5). The amount of metal atoms was two hundred times of the cofactor. The yields were calculated relative to the metal-free sample (100% NMN). To determine the TOF of each reaction, the sum of 1,4-NMNH, 1,4,6-products, and 1,2,4,6-product was considered as the amount of product. Fe-containing experiments were performed in duplicate and Ni-containing in triplicate.

	H_2	NMN	SD	1,4-NMNH	SD	1,4,6-products	SD	NMNH$_2$OH	SD	1,2,4,6-product	SD	Nam	SD	TOF [s^{-1}]
4h	μNi^0	0.70%	0.5%	1.04%	1.2%	37.05%	3.0%	4.02%	3.1%	11.49%	3.1%	0.21%	0.3%	1.50E-07
	μFe^0	20.07%	17.8%	44.73%	20.3%	0.23%	0.0%	9.53%	4.2%	0.81%	0.3%	25.43%	5.5%	1.39E-07

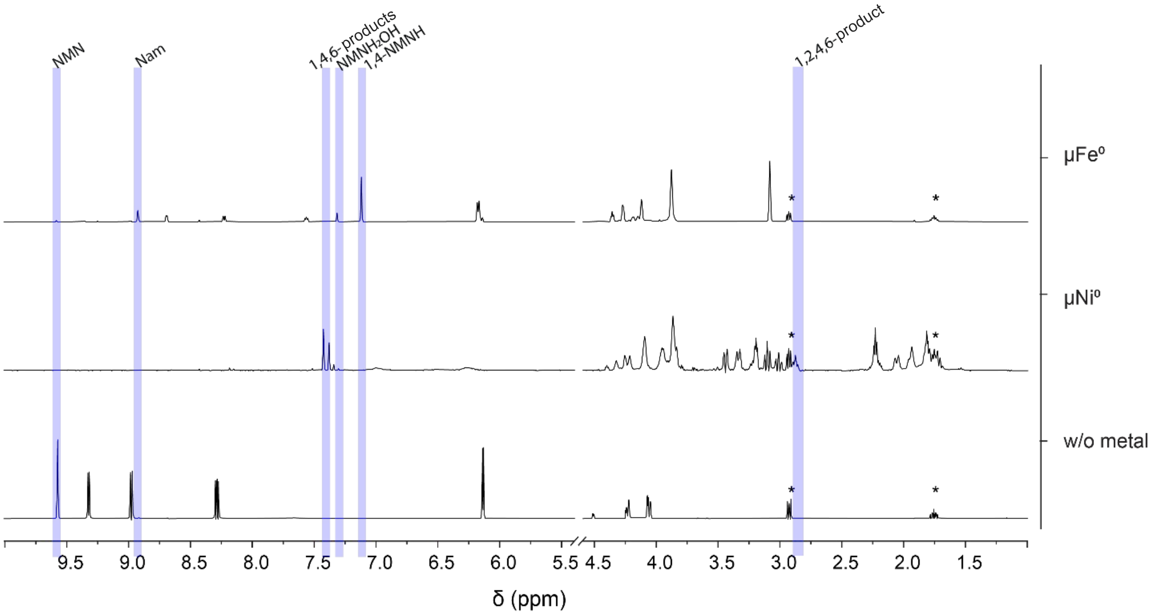

Fig. S33 The NMR spectra of replica samples of 18 μmol NMN in PBS (0.5 M, pH 8.5) with 5 bar of H_2 and 3.2 mmol μFe^0, μNi^0 (200:1 cofactor ratio), or no metal, as shown in **Scheme S14**, are stacked together in this figure. After the 4h reaction, the supernatant was collected and DSS added as an internal standard. The spectra were edited to only include relevant peaks, having been removed a DSS peak at 0 ppm and water peak at 4.8 ppm. No other peaks were found in the areas removed. Some DSS peaks are still visible (*). The peaks used for qualitative analysis and subsequent qNMR are highlighted in blue, according to **Table S8.**

NAD⁺ configurations in solution and on Ni surface

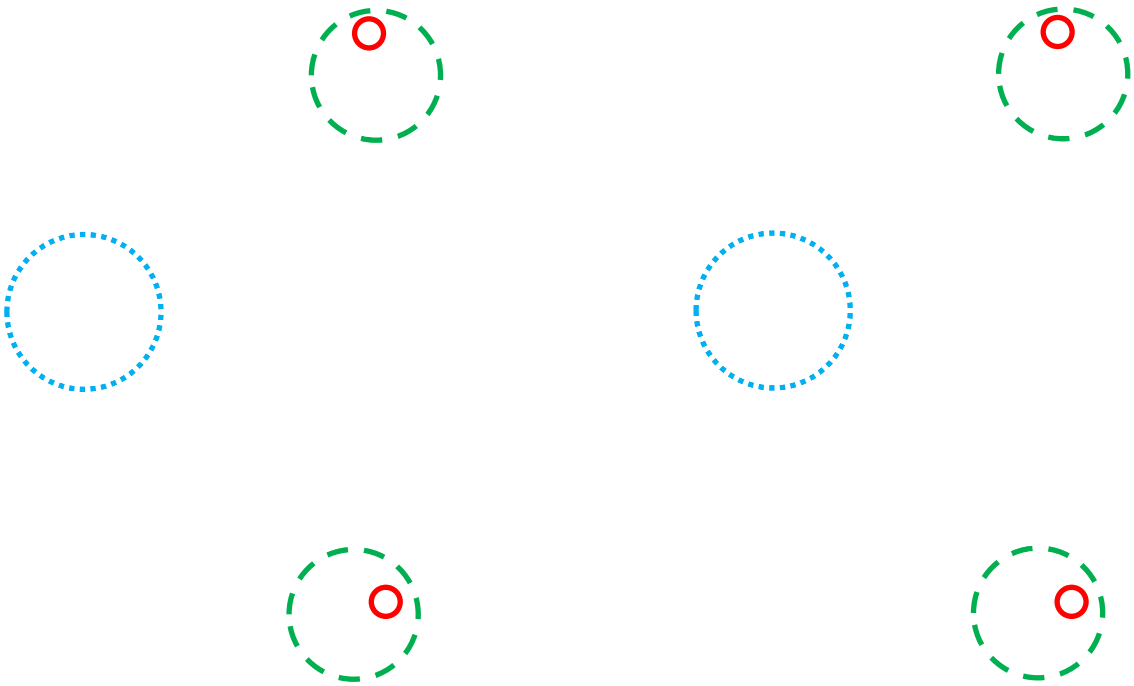

Fig. S34 a) NAD+, b) NADH, c) NMN and d) NMNH. The C-4 position is circled in red, the nicotinamide ring is circled with a green dashed line and the adenosine rings are circled in blue dotted line.

Table S15 Molecular systems studies in this work.

System name	Ni surface	Organics	Starting config.	Solvent composition, no. molecules	Repeats
Ni + NAD^+	Y	NAD^+	random	~9370 water, 20 Na^+	3
Ni + NAD^+ (folded)	Y	NAD^+	folded	~9410 water, 20 Na^+	3
Ni + NMN	Y	NMN	--	~9510 water, 20 Na^+	3
Ni + NADH	Y	NADH	random	~9375 water, 30 Na^+	3
Ni + NMNH	Y	NMNH	---	~9500 water, 30 Na^+	3
NAD	N	NAD^+	random	3773 water, 10 Na^+	1

NAD$^+$ and NADH are known to exist in open or closed configurations, which were analyzed with radius of gyration and minimal distances between nicotinamide and adenosine rings. The average distributions are given in **Fig. S35** and **Fig. S41**.

In solution, NAD$^+$ rapidly changes its configuration from open to close (**Fig. S41a**), spending nearly half of the time (40-50%) in a folded state (with at least one contact). These transitions occur at a rate of one event per 1 ns per molecule.
When adsorbed on Ni surface, NAD$^+$ also is found in both open and closed configurations (**Fig. S41b**, **Fig. S36**), with 30-40% being folded. Unlike in solution these configurations are not as dynamic, being stabilized by the surface. Only rare opening/closing events are observed at approximately one event per 17 ns per molecule.

a) *b)*

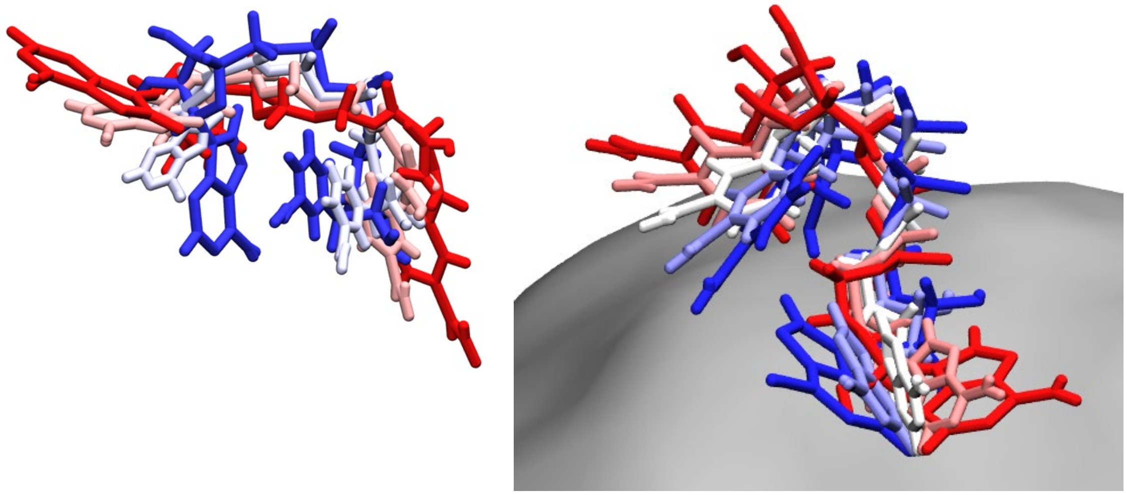

Fig. S35 Rendering of NAD$^+$ converting from open/unfolded (red) to closed/folded (blue) a) in water and b) adsorbed to Ni surface.

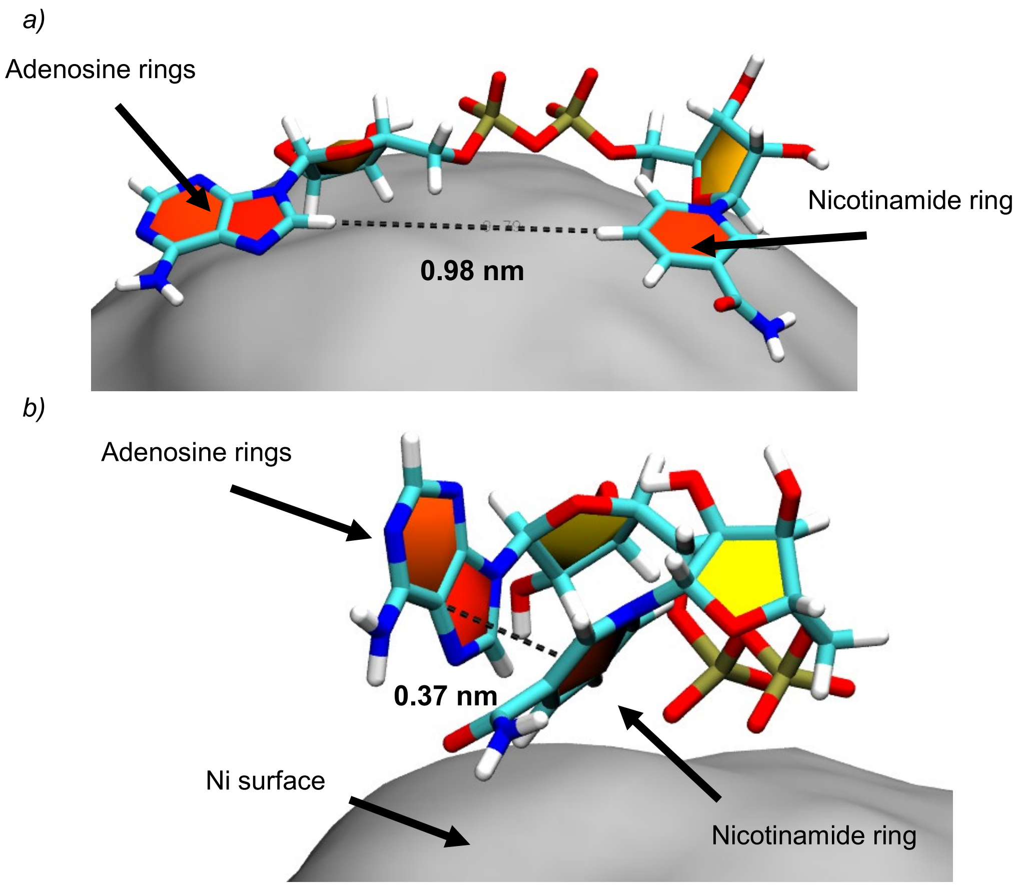

Fig. S36 Rendering of a) folded and b) open NAD$^+$ adsorbed to Ni surface.

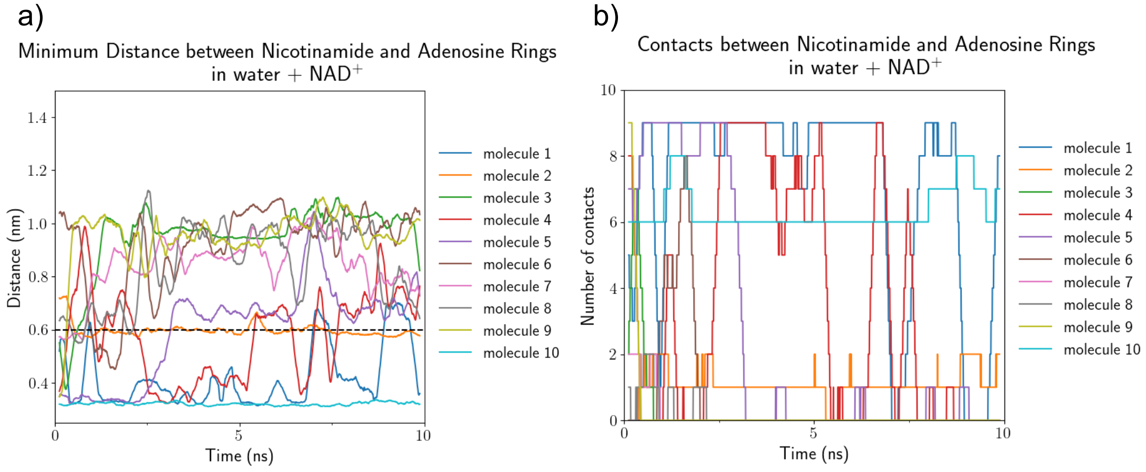

Fig. S37 Folding dynamics of NAD$^+$ in water showing a) the minimum distance between nicotinamide and adenosine rings and b) the corresponding number of contacts.

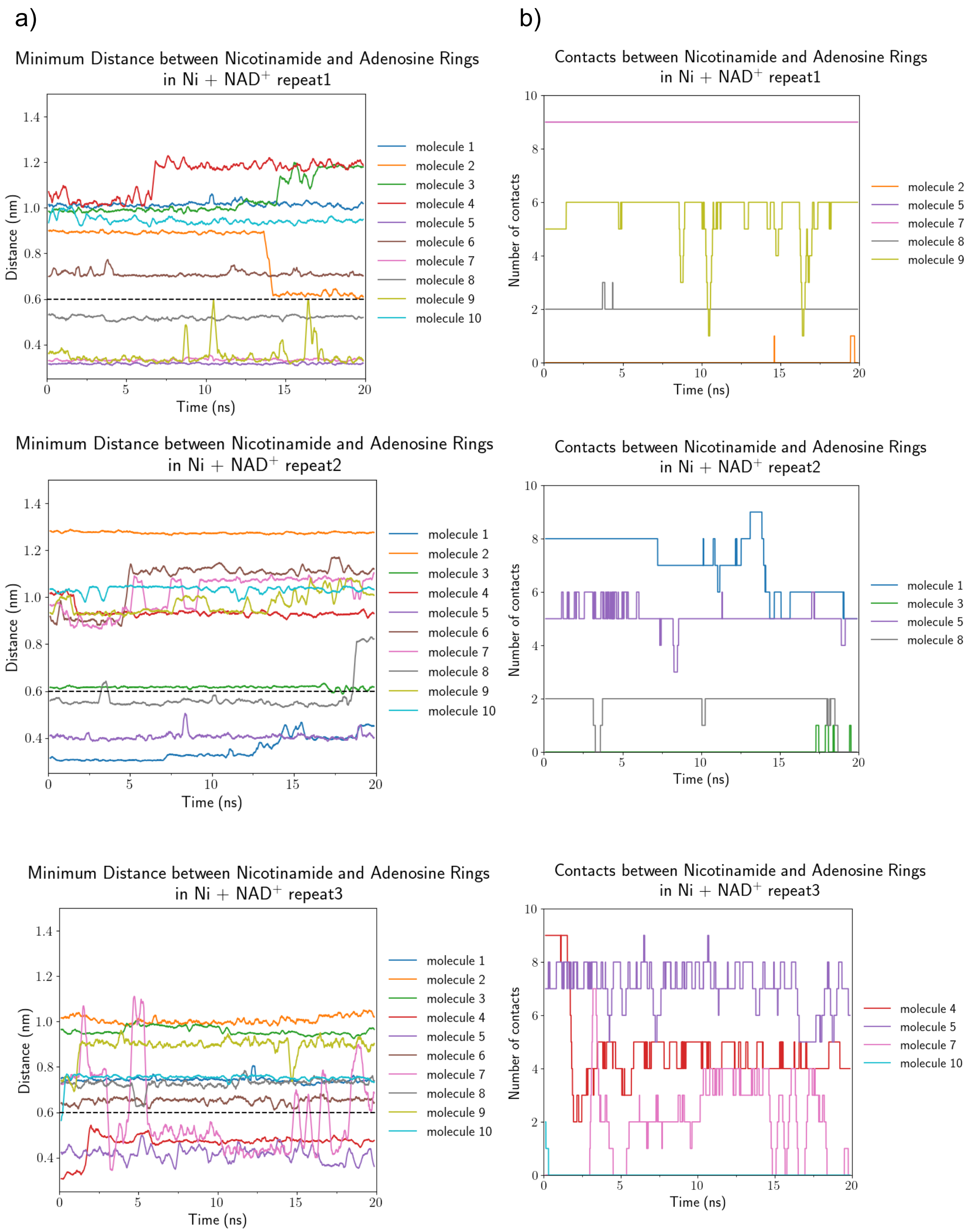

Fig. S38 Folding dynamics of NAD^+ with Ni sphere showing a) the minimum distance between nicotinamide and adenosine rings and b) the corresponding number of contacts.

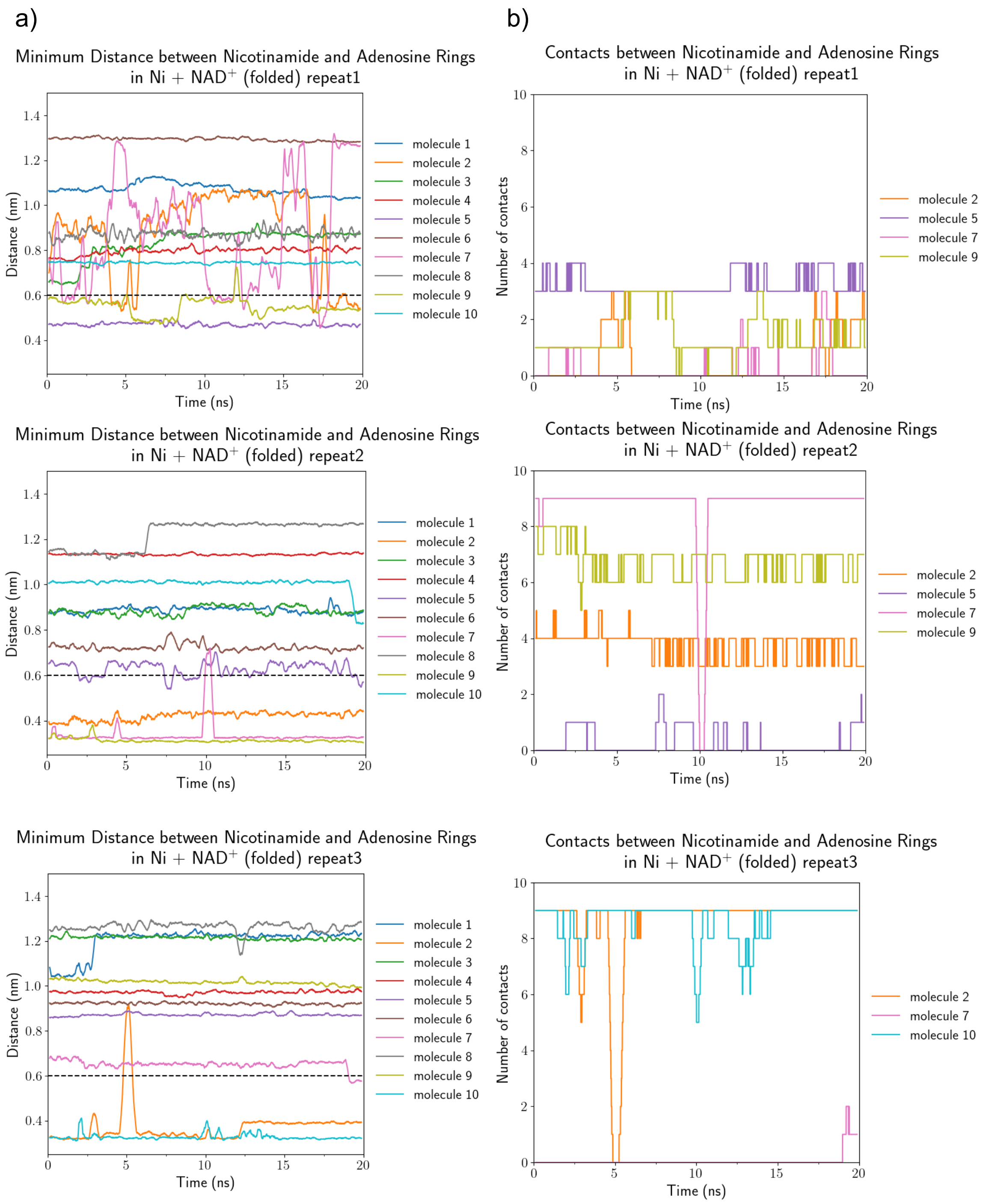

Fig. S39 Folding dynamics of NAD^+ (folded) with Ni sphere showing a) the minimum distance between nicotinamide and adenosine rings and b) the corresponding number of contacts.

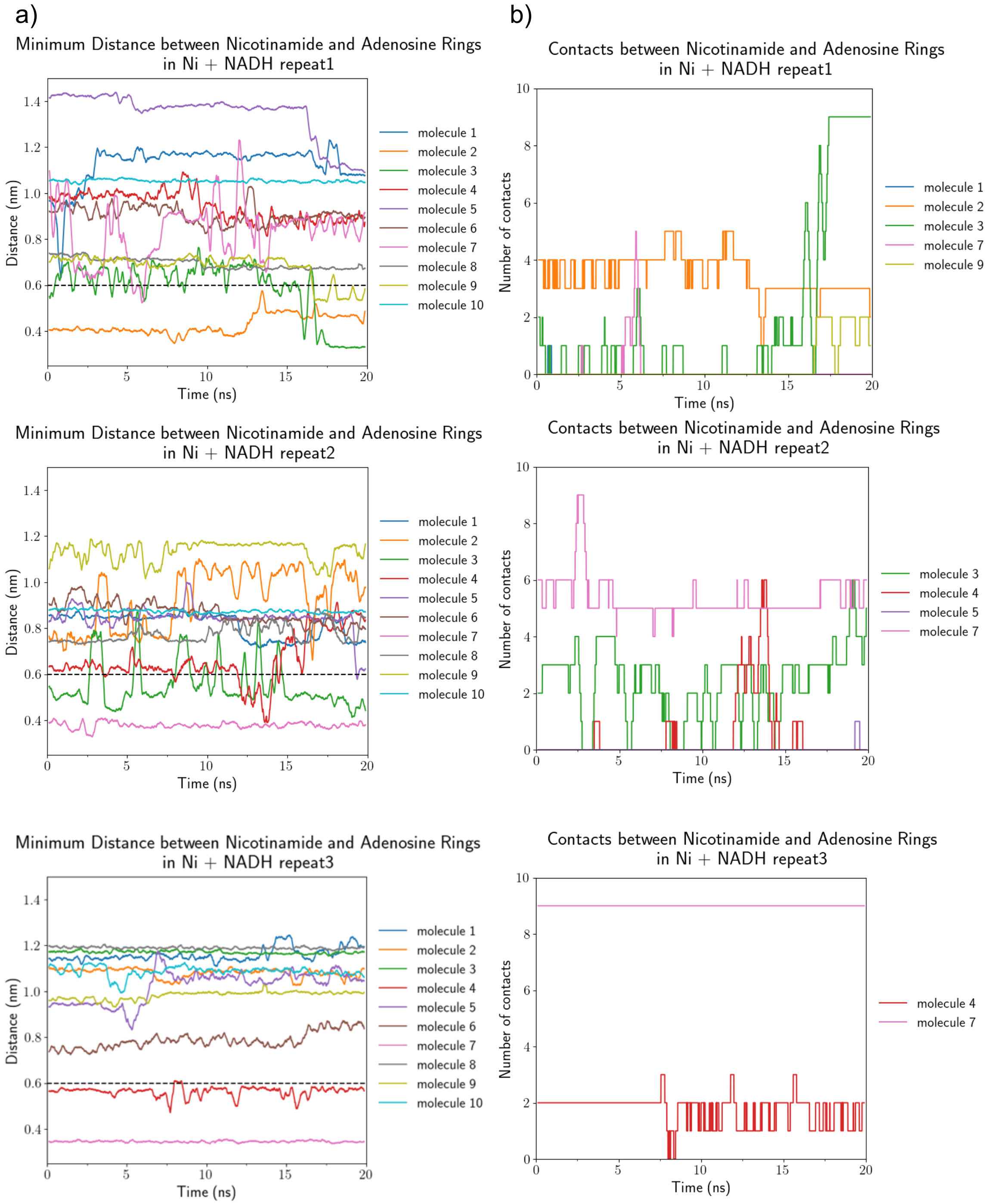

Fig. S40 Folding dynamics of NADH with Ni sphere showing a) the minimum distance between nicotinamide and adenosine rings and b) the corresponding number of contacts.

The simulation of NAD⁺ that was initiated with closed configurations did not show any different results from the random system. This is likely due to fast open/close dynamics in the solution, allowing for the configurations to change prior to adsorption. NADH also follows similar dynamics to NAD⁺, yet short distances between the rings are not as pronounced (**Fig. S40c**) with 20-30% being folded, likely due to aromatic stacking being no longer available.

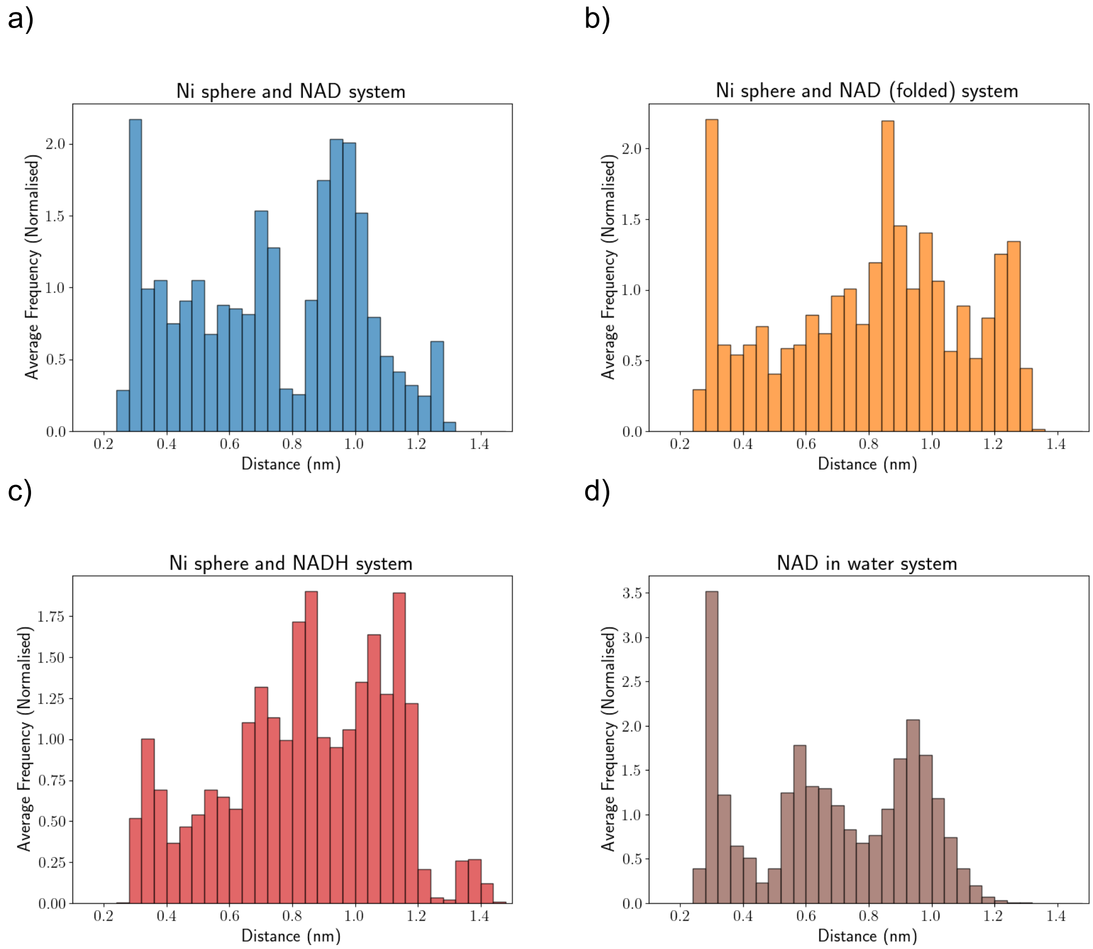

Fig. S41 The distance between the nicotinamide and adenosine rings in a) Ni and NAD⁺, b) Ni and NAD⁺ (initiated as folded), c) Ni and NADH, and d) NAD⁺ in water systems.

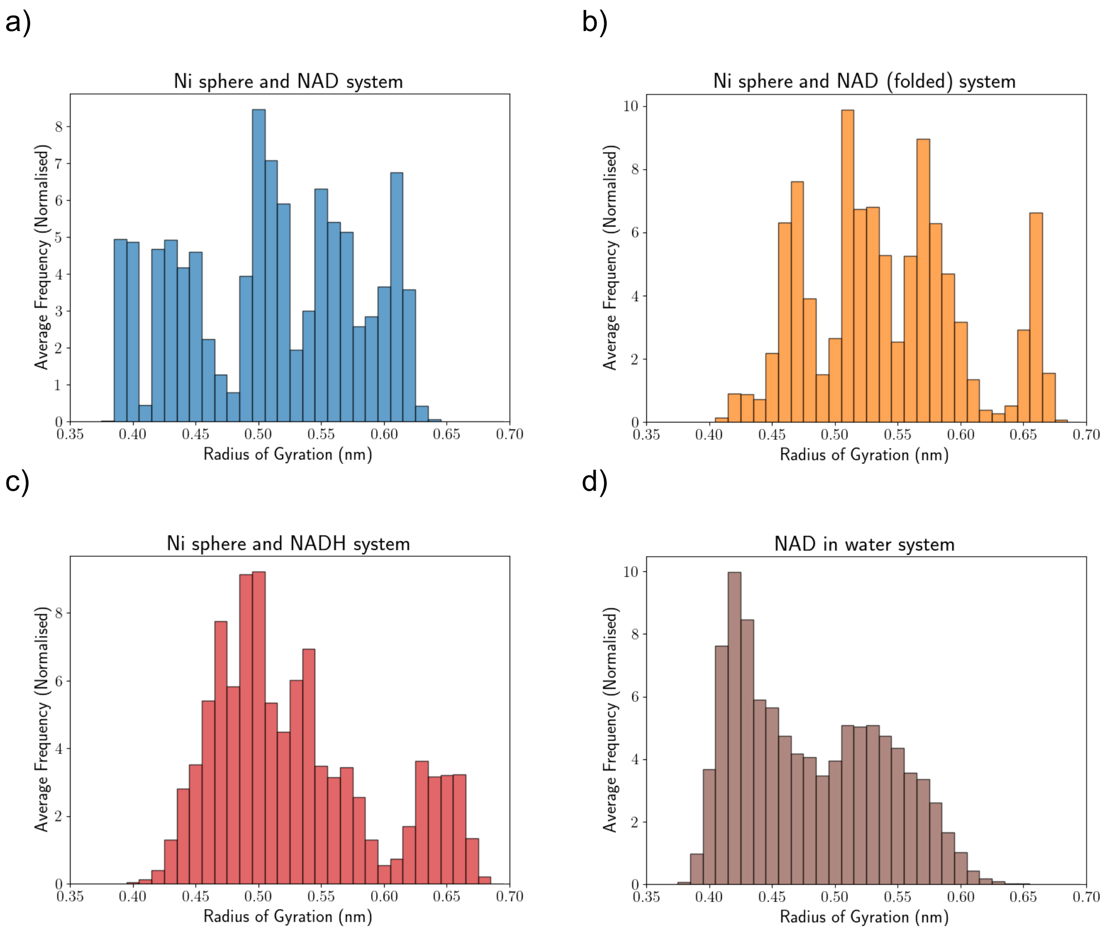

Fig. S42 The radius of gyration of NAD^+/NADH in a) Ni and NAD^+ , b) Ni and NAD^+ (initiated as folded), c) Ni and NADH, and d) NAD^+ in water systems.

Interaction of NAD^+, NMN, NADH and NMNH with Ni surface

Adsorption of the organic molecule to Ni surface was quantified with RDF (**Fig. S43** and **Table S16**). It can be seen that NAD^+ is adsorbed with C-4 closer to the surface (0.286 nm) than NMN (0.296 nm) with a higher g(r) peak, indicating more ordering of adsorbed species of NAD^+ than NMN. On the other hand, the number of adsorbed species of NMN at the first peak is slightly higher (9.2 molecules) than it is for NAD^+ (8.6 – 8.7 molecules).

When the molecules are reduced to NADH and NMNH on the C-4, the distance to Ni is slightly increased, compared to their non-reduced form, 0.308 nm for NADH and 0.320 nm for NMNH. The g(r) peak, on other hand, is lowered, indicating reduction of ordering. Interestingly, the coordination numbers for NADH are lower with respect to NAD^+ (7.9 vs 8.7 molecules, respectively), while the opposite is seen for the NMNH (10 molecules vs 9.2 molecules for NMNH and NMN, respectively).

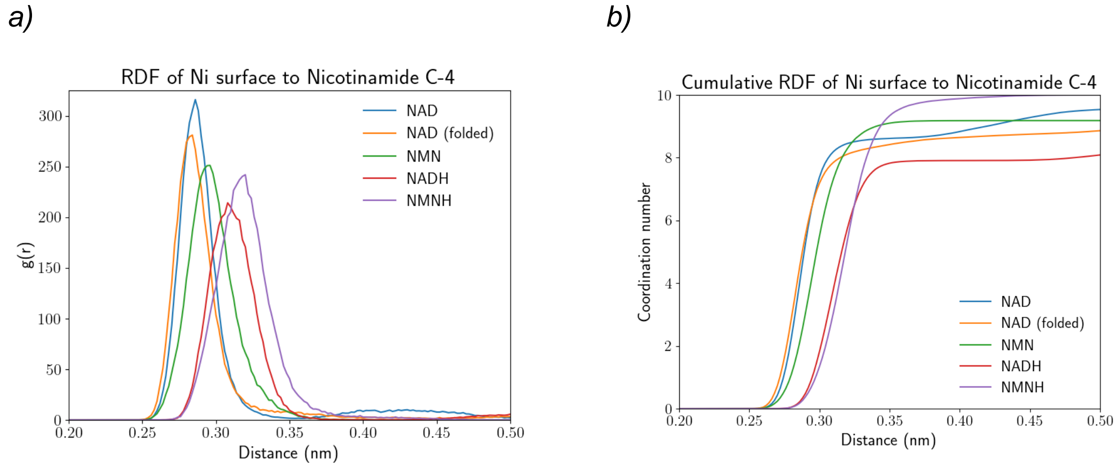

Fig. S43 The a) radial distribution function and b) cumulative radial distribution function of the Ni surface to the organic's nicotinamide C-4.

Table S16 Radial distribution analysis summary for distances from Ni surface atoms to nicotinamide C-4. The table is presenting first peak height, position and associated coordination number (CN). Maximum possible CN is equivalent to the total number of molecules, i.e., 10 molecules.

System	First peak, nm	Peak height, g(r)	CN at first peak
Ni + NAD^+	0.286	316	8.6
Ni + NAD^+ (folded)	0.284	281	8.7
Ni + NADH	0.308	214	7.9
Ni + NMN	0.296	251	9.2
Ni + NMNH	0.320	242	10.0

Adsorption Mechanism and Dynamics

Overall, NAD^+ adsorbs in two configurations – open and closed (**Fig. S34** and **Fig. S44a**). These configurations can change while molecule is adsorbed (**Fig. S34b**). Upon reduction to NADH, the molecule remains adsorbed in a similar manner through nicotinamide ring (**Fig. S44b**), yet the adsorption is less strong.

NMN also interacts with the Ni surface is a similar way as NAD^+, yet a slightly longer distance is found between surface-forming Ni atoms and C-4 of NMN (**Fig. S43b**). Upon reduction, similarly to NADH, the distance to the Ni surface for NMNH is increased to accommodate for the new configuration on nicotinamide C-4 atom.

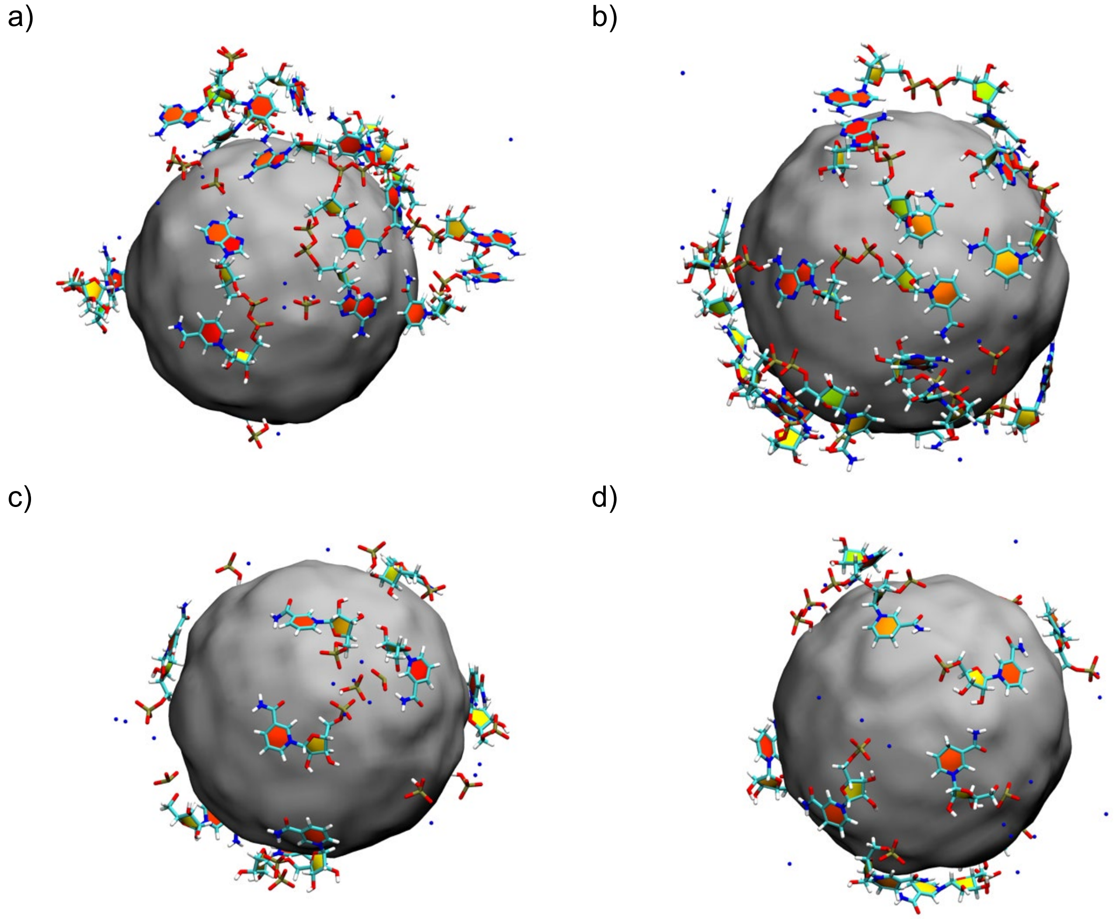

Fig. S44 Renderings of the final frame of the simulation of a) NAD^+ , b) NADH, c) NMN and d) NMNH interacting with Ni sphere.

NMNH stability and reactivity with and without metal

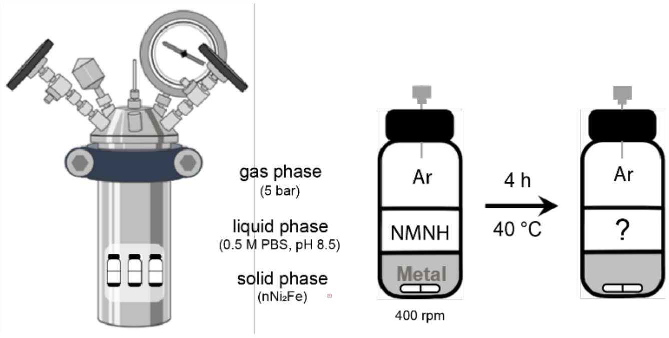

Scheme S9 The stability of NMNH with (nNi_2Fe) and without metal was tested with the protocol described in detail in **Methods**, and according to the scheme above. The amount of 1.4-NMNH was 36 µmol and metal was the same (1:1), which reacted together for 4 h, at 40 °C, under alkaline conditions and 5 bars of Ar.

Table S17 After 4 h under 5 bar of Ar. as shown in **Scheme S9Scheme S14**. samples with (Ni_2Fe) and without metal resulted in similar amounts of NMN, $NMNH_2OH$, and nicotinamide (Nam), from the starting material 1,4-NMNH. The starting metal and cofactor were 36 µmol each, mixed in 3 mL of 0,133 M PBS (pH 8.5). The yields were calculated relative to the initial amount of 1,4-NMNH.

	Ar	NMN	SD	1,4-NMNH	SD	1,4,6-products	SD	$NMNH_2OH$	SD	1,2,4,6-product	SD	Nam	SD
4h	Ni_2Fe	1.28%	0.05%	59.52%	2.02%	0.00%	0.0%	2.86%	0.12%	0.00%	0.0%	2.76%	0.09%
	w/o metal	1.14%	0.05%	55.81%	0.51%	0.00%	0.0%	4.61%	0.23%	0.00%	0.0%	2.77%	0.08%

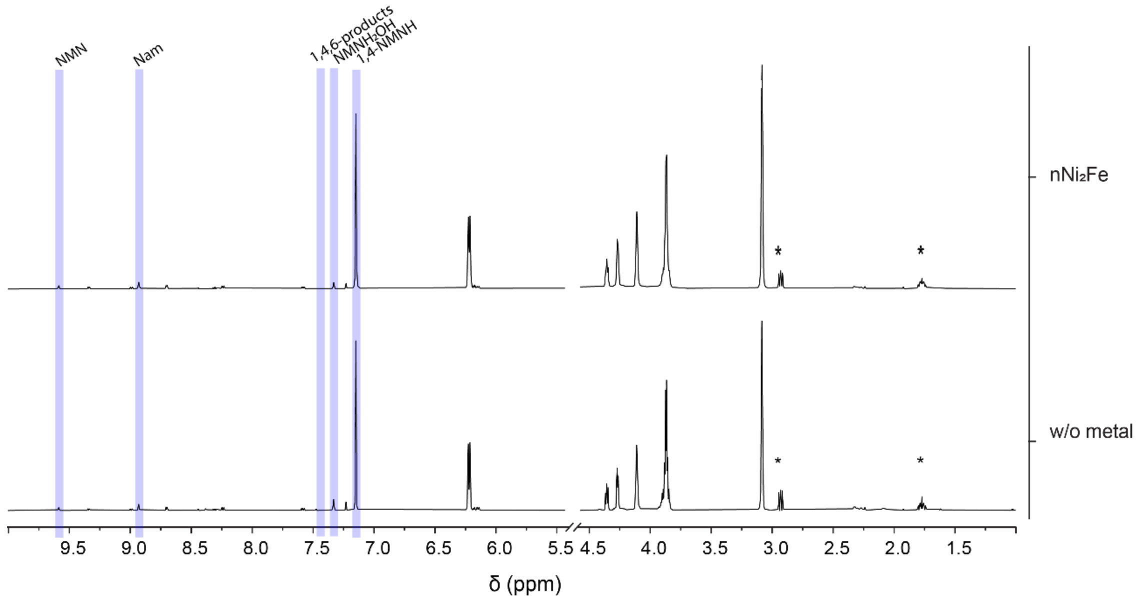

Fig. S45 The NMR spectra of replica samples of 36 μmol 1,4-NMNH in PBS (0.133 M, pH 8.5) with 5 bar of Ar and 36 μmol of Ni_2Fe, or no metal, as shown in **Scheme S9**, are stacked in this figure. After the 4h reaction, the supernatant was collected and DSS added as an internal standard. The spectra were edited to only include relevant peaks, having been removed a DSS peak at 0 ppm and water peak at 4.8 ppm. No other peaks were found in the areas removed. Some DSS peaks are still visible (*). The peaks used for qualitative analysis and subsequent qNMR (**Table S17**) are highlighted in blue, according to **Table S8.**

Proposed surface interaction between NAD/NMN and Ni/Fe minerals

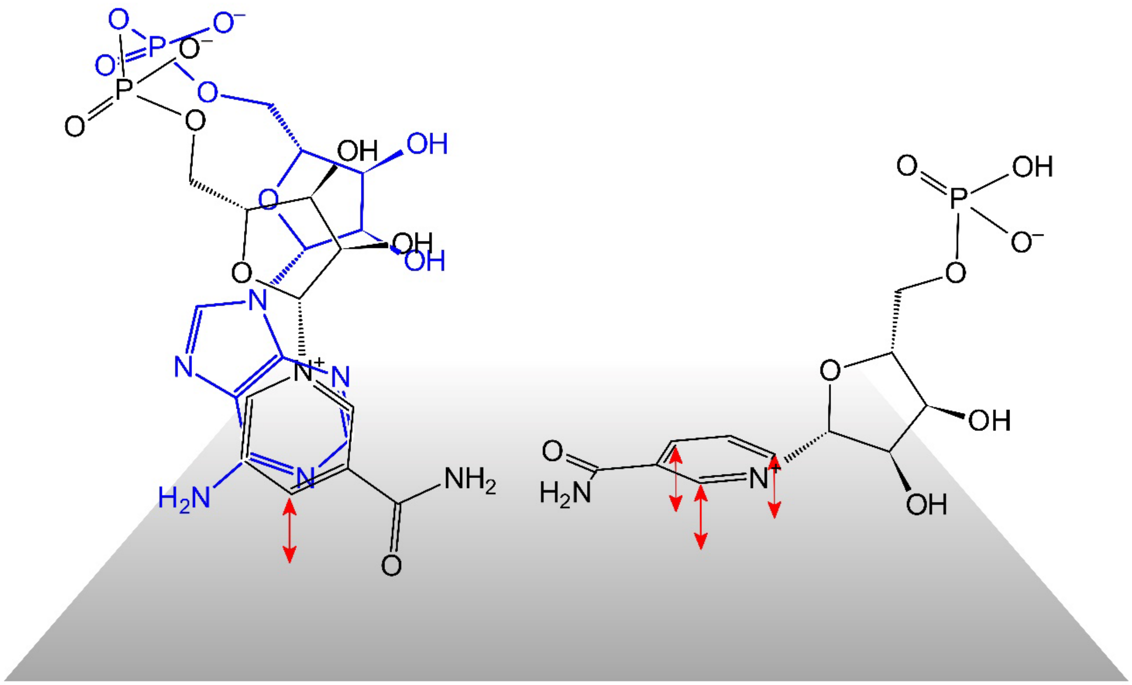

Scheme S10 Proposed surface interaction with NAD^+ and NMN. Due to its dinucleotide structure. NAD alternates between the folded and open conformation. This could prevent surface hydrides from reaching all carbons of the nicotinamide ring. NMN does not have such conformation and would be able to interact more directly with the surface.

Cyclic Voltammetry of standards and reaction products

Standards were prepared at 1 mM of each compound in PBS (0.133 M, pH 8.5) at 25ºC, a three-electrode electrochemical cell was prepared for cyclic voltammetry with glassy carbon as working electrode, platinum wire as auxiliary electrode and $Ag/AgNO_3$ (0.01 M) as reference electrode.

After a 4 h long reaction as described in **Scheme S5** and **Scheme S6** the supernatant was diluted with PBS (0.133 M at pH 8.5) to obtain 15 mL of sample and a similar concentration to the standards (1 mM). Previous literature on NAD cyclic voltammetry was consulted for the CV interpretation[17].

Maintaining the same experimental setup (three-electrode electrochemical cell), the diluted samples were measured starting from a anodic current.

Table S18 Table of reduction potential

Reagent	E_a (V vs. SCE)[a]
1,4-NADH	0.531
1,4-NMNH	0.609
Reduced NAD^+	0.573
Reduced NMN	0.616; 1.000

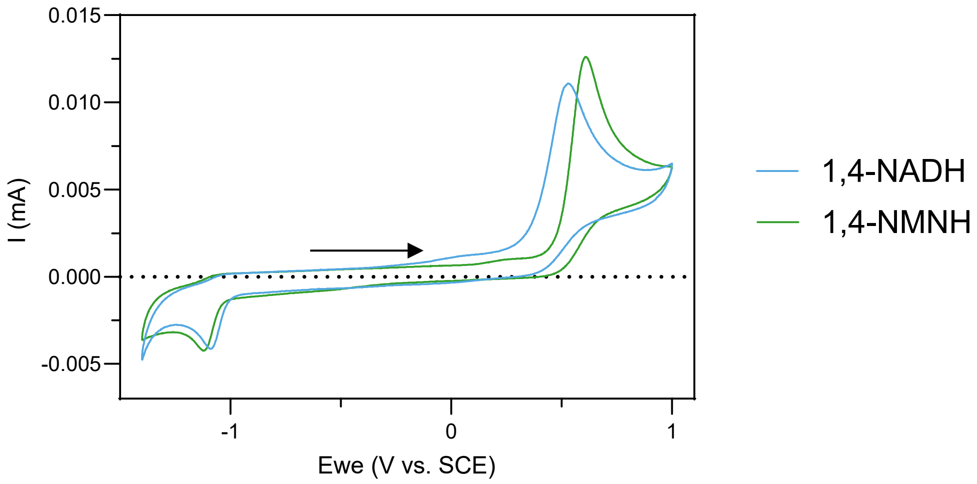

Fig. S46 Cyclic voltammogram of 1,4-NADH (1 mM; blue) and 1,4-NMNH (1 mM; green) in PBS (pH 8.5) at 25 ºC.

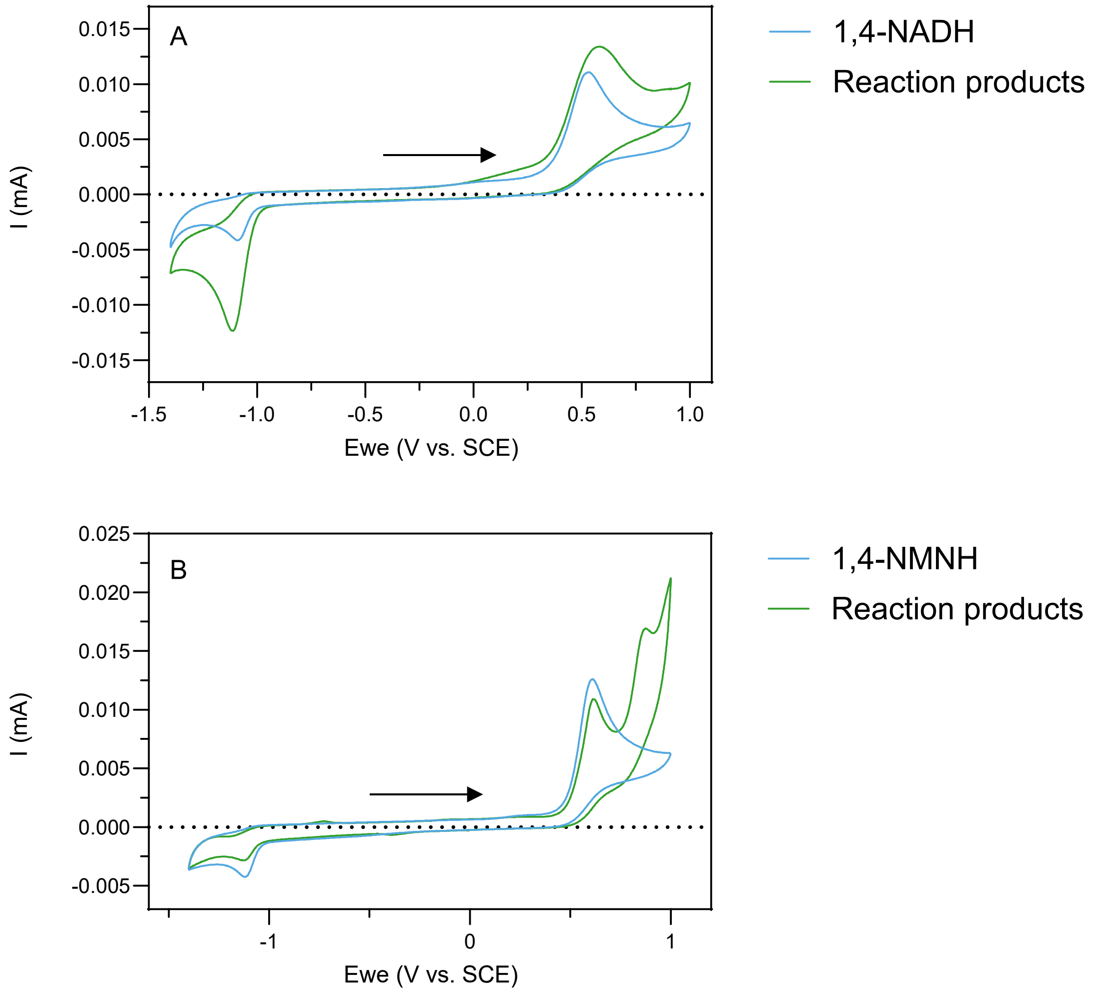

Fig. S47 A) Cyclic voltammogram of 1,4-NADH (1 mM in 0.133 M PBS pH 8.5; blue) and 12 mM of reduced NAD^+ with equimolar amounts of nNiFe (0.133 M PBS. 40 °C. 5 bar H_2.diluted 1:5 after reaction; green), measured at 25 ºC. B) Cyclic voltammogram of 1,4-NMNH (1 mM in 0.133 M PBS pH 8.5; blue) and 12 mM of reduced NMN with equimolar amounts of nNiFe (0.133 M PBS. 40 °C. 5 bar H_2.diluted with PBS 1:5 after reaction; green). measured at 25 ºC.

Carbonate buffer vs Phosphate buffer

Phosphate and iron-phosphate can have catalytic properties and influence reaction mechanisms. To test weather this was or not the case under our experimental setting, some of the μFe experiments were repeated with a different inorganic buffer: carbonate buffer. To have the necessary pH stability, the carbonate buffer concentration was 0.75 M, instead of the 0.5 used for PBS.

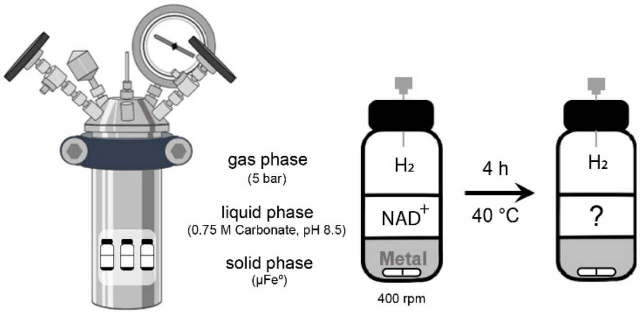

Scheme S11 The reduction of NAD^+ with μFe^0 (particle size: <150 μm) was tested with the protocol described in detail in **Methods**, and according to the scheme above. The amount of NAD^+ was 36 μmol and metal was 1.8 mmol, which reacted together for 4 h, at 40 °C, under alkaline conditions and 5 bars of H_2.

Table S19 After 4 h under 5 bar of H_2, as shown in **Scheme S13** and **Scheme S11**, samples with μFe^0 yielded similar amounts of 1,4-NADH, 1,6-NADH, from the starting material NAD^+, regardless of the buffer used. The starting metal and cofactor were 36 μmol mixed in 3 mL of 0.75 M Carbonate buffer or 0.5 PBS (pH 8.5). The amount of metal atoms was 50 times the moles of cofactor. The yields were calculated relative to the metal-free sample (100% NAD^+). To determine the TOF of each reaction, the sum of 1,4-NADH, and 1,6-NADH was considered as the amount of product. PBS samples had duplicates while Carbonate had quadruplets.

	H_2	**NAD^+**	**SD**	**1,4-NADH**	**SD**	**1,6-NADH**	**SD**	**Nam**	**SD**	**TOF [s^{-1}]**
4h	Carbonate	42,16%	7,1%	7,46%	0,1%	1,90%	0,1%	32,94%	2,8%	1,37E-07
	PBS	57,52%	2,9%	8,12%	1,2%	1,60%	0,2%	27,57%	2,2%	1,43E-07

The reduction of NAD^+ with Fe^0 in carbonate buffer led to a wider variety of uncharacterizable side products (Fig. S51) than during reactions in phosphate buffer (Fig. S48). The peaks of the side products are less defined than the peak of 1,6-NADH which appears in the same region of the spectra. This effect was also visible in controls without Fe^0, although in even lesser amounts. The amount of each of the previously characterized main products (1,4-NADH, 1,4-NMNH and MNHOH), however, is affected.

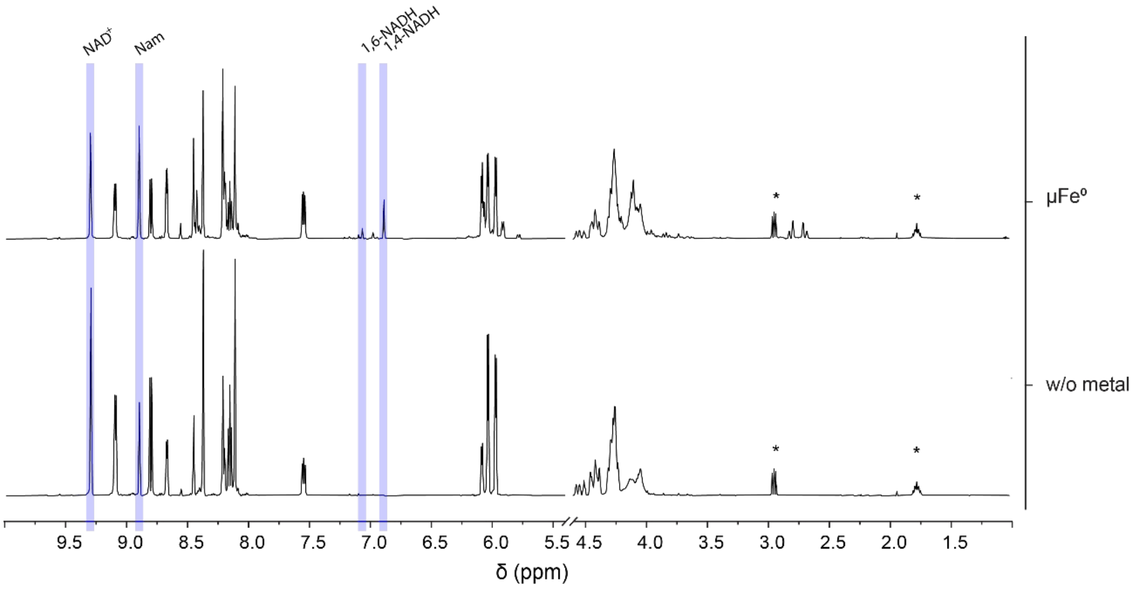

Fig. S48 The NMR spectra of replica samples of 36 µmol NAD^+ in Carbonate buffer (0.75 M, pH 8.5) with 5 bar of H_2 and 1.8 mmol μFe^0 (50:1 cofactor ratio), or no metal, as shown in **Scheme S11**, are stacked together in this figure. After the 4h reaction, the supernatant was collected and DSS added as an internal standard. The spectra were edited to only include relevant peaks, having been removed a DSS peak at 0 ppm and water peak at 4.8 ppm. No other peaks were found in the areas removed. Some DSS peaks are still visible (*). The peaks used for qualitative analysis and subsequent qNMR are highlighted in blue, according to **Table S1**.

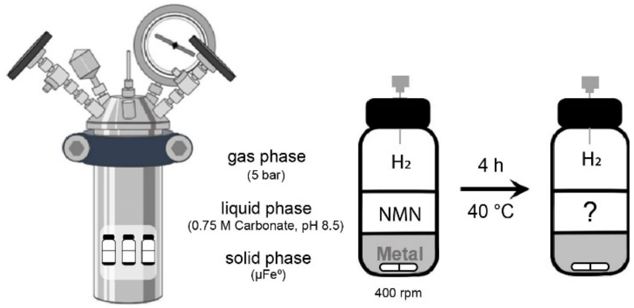

Scheme S12 The reduction of NMN with μFe^0 (particle size: <150 µm) was tested with the protocol described in detail in **Methods**, and according to the scheme above. The amount of NAD^+ was 36 µmol and metal was 1.8 mmol, which reacted together for 4 h, at 40 °C, under alkaline conditions and 5 bars of H_2.

Table S20 After 4 h under 5 bar of Ar, as shown in **Scheme S14** and **Scheme S12**, samples with μFe^0 yielded similar amounts of 1,4-NMNH, $NMNH_2OH$, and nicotinamide (Nam), from the starting material NMN. The starting metal and cofactor were 1.8 mmol and 36 μmol, respectively, mixed in 3 mL of 0.75 M Carbonate buffer or 0.5 M PBS (pH 8.5). The amount of metal atoms was fifty times of the cofactor. The yields were calculated relative to the metal-free sample (100% NMN). To determine the TOF of each reaction, the sum of 1,4-NMNH, 1,4,6-products, and 1,2,4,6-product was considered as the amount of product. PBS samples were duplicates while carbonate were quadruples.

	H_2	NMN	SD	1,4-NMNH	SD	1,4,6-products	SD	$NMNH_2OH$	SD	1,2,4,6-product	SD	Nam	SD	TOF [s^{-1}]
4h	Carb.	4,52%	3,2%	45,57%	4,5%	0,00%	0,2%	11,58%	1,3%	0,00%	0,0%	10,50%	0,7%	3,44E-07
	PBS	23,48%	15,8%	47,95%	11,9%	0,00%	0,0%	11,43%	2,0%	0,00%	0,0%	14,88%	12,8%	1,72E-07

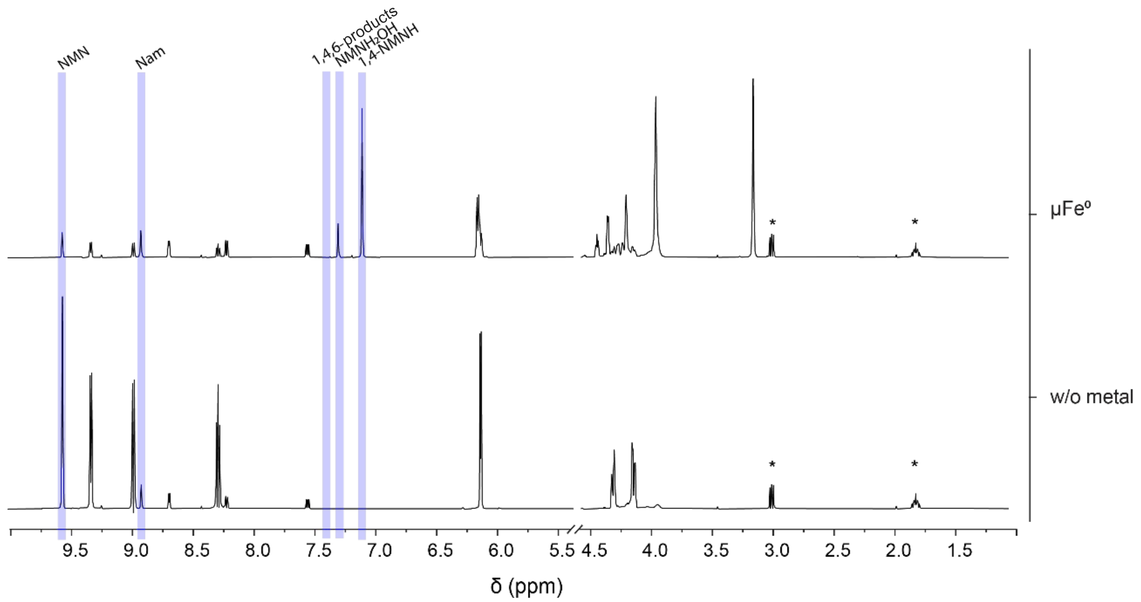

Fig. S49 The NMR spectra of replica samples of 36 μmol NMN in Carbonate buffer (0.75 M, pH 8.5) with 5 bar of H_2 and 1.8 mmol μFe^0 (50:1 cofactor ratio), or no metal, as shown in **Scheme S12**, are stacked together in this figure. After the 4h reaction, the supernatant was collected and DSS added as an internal standard. The spectra were edited to only include relevant peaks, having been removed a DSS peak at 0 ppm and water peak at 4.8 ppm. No other peaks were found in the areas removed. Some DSS peaks are still visible (*). The peaks used for qualitative analysis and subsequent qNMR are highlighted in blue, according to **Table** S8.

Extra peaks were also found in the NMN carbonate samples, as they were in the samples with NAD^+, leading to a significant loss of the starting material (Table S20), but without affecting the main products yield. In conclusion, phosphate is seemingly less reactive and thus a more reliable buffer.

Reduction of NAD/NMN with μNi^0 and μFe^0

In an attempt to further understand the differences observed in reactions with NMN and Ni/Fe alloys as catalysts, compared to NAD, experiments with the individual metals in micropowder form were performed.

For quantification of each product, the ppm values of **Table S1**, and **Table S8** should be considered.

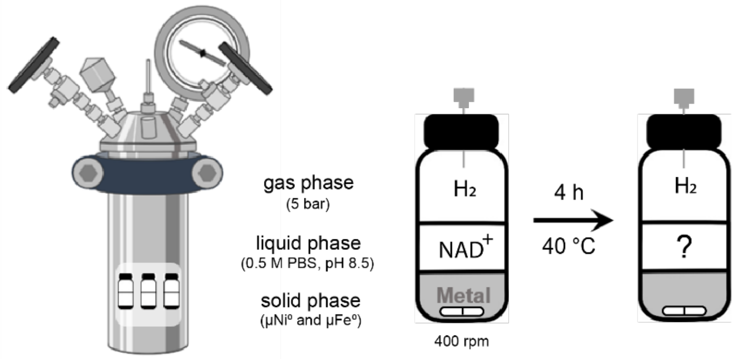

Scheme S13 The reduction of NAD^+ with μFe^0 (particle size: <150 μm) and μNi^0 (particle size: 3–7 μm) was tested with the protocol described in detail in Methods, and according to the scheme above. The amount of NAD^+ was 36 μmol and metal was 1.8 mmol, which reacted together for 4 h, at 40 °C, under alkaline conditions and 5 bars of H_2.

Table S21 After 4 h under 5 bar of H_2, as shown in **Scheme S13**, samples with μNi^0 or μFe^0 yielded different amounts of 1,4-NADH, 1,6-NADH, and Nam, from the starting material NAD^+. The starting metal and cofactor were 36 μmol mixed in 3 mL of 0.5 M PBS (pH 8.5). The amount of metal atoms was 50 times the moles of cofactor. The yields were calculated relative to the metal-free sample (100% NAD^+). To determine the TOF of each reaction, the sum of 1,4-NADH, and 1,6-NADH was considered as the amount of product. All conditions had duplicates and a metal-free control.

	H_2	**NAD^+**	**SD**	**1,4-NADH**	**SD**	**1,6-NADH**	**SD**	**Nam**	**SD**	**TOF [s^{-1}]**
4h	μNi^0	53.12%	1.8%	19.89%	0.2%	5.26%	0.1%	11.21%	0.0%	3.69E-07
	μFe^0	57.52%	2.9%	8.12%	1.2%	1.60%	0.2%	27.57%	2.2%	1.43E-07

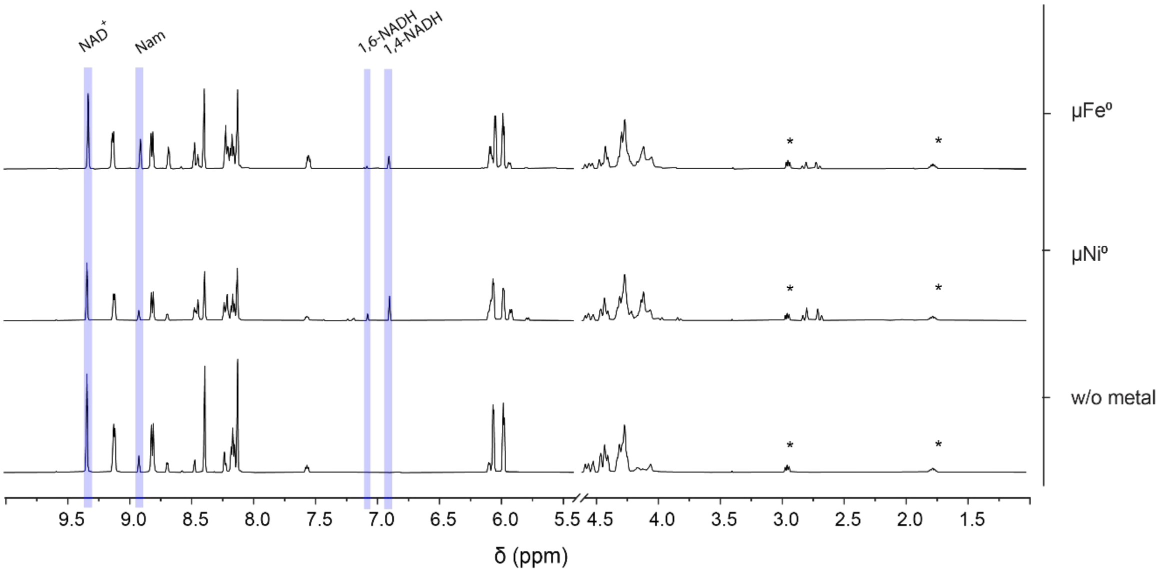

Fig. S50 The NMR spectra of replica samples of 36 μmol NAD^+ in PBS (0.5 M, pH 8.5) with 5 bar of H_2 and 1.8 mmol μFe^0, μNi^0 (50:1 cofactor ratio), or no metal, as shown in **Scheme S13**, are stacked together in this figure. After the 4h reaction, the supernatant was collected and DSS added as an internal standard. The spectra were edited to only include relevant peaks, having been removed a DSS peak at 0 ppm and water peak at 4.8 ppm. No other peaks were found in the areas removed. Some DSS peaks are still visible (*). The peaks used for qualitative analysis and subsequent qNMR are highlighted in blue, according to **Table S1**.

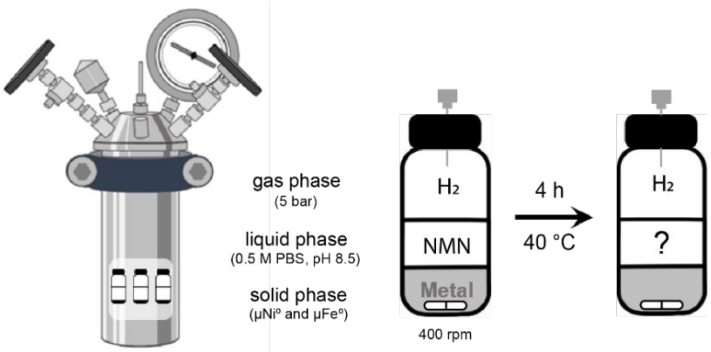

Scheme S14 The reduction of NMN with μFe^0 and μNi was tested with the protocol described in detail in **Methods**, and according to the scheme above. The amount of NMN was 36 μmol and metal was 1.8 mmol (50:1) or 18 μmol and 3.6 mmol respectfully (200:1), which reacted together for 4 h, at 40 °C, under alkaline conditions and 5 bars of H_2. The same reaction was made under Ar as a control.

Table S22 After 4 h under 5 bar of H_2, as shown in **Scheme S14**, samples with μNi^0 or μFe^0 yielded different amounts of 1,4-NMNH, 1,4,6-products, $NMNH_2OH$, and nicotinamide (Nam), from the starting material NMN. The starting metal and cofactor were 1.8 mmol and 36 μmol, respectively, mixed in 3 mL of 0.5 M PBS (pH 8.5). The amount of metal atoms was fifty times of the cofactor. The yields were calculated relative to the metal-free sample (100% NMN). To determine the TOF of each reaction, the sum of 1,4-NMNH, 1,2,4,6-product, and 1,4,6-products was considered as the amount of product. All conditions were tested in triplicate.

	H_2	**NMN**	**SD**	**1,4-NMNH**	**SD**	**1,4,6-products**	**SD**	**$NMNH_2OH$**	**SD**	**1,2,4,6-product**	**SD**	**Nam**	**SD**	**TOF [s^{-1}]**
4h	μNi^0	0.92%	0.4%	4.85%	3.47%	44.13%	7.07%	13.79%	4.5%	9.83%	3.1%	0.50%	0.3%	3.55E-07
	μFe^0	23.48%	15.8%	47.95%	11.9%	0.00%	0.0%	11.43%	2.0%	0.00%	0.0%	14.88%	12.8%	1.72E-07

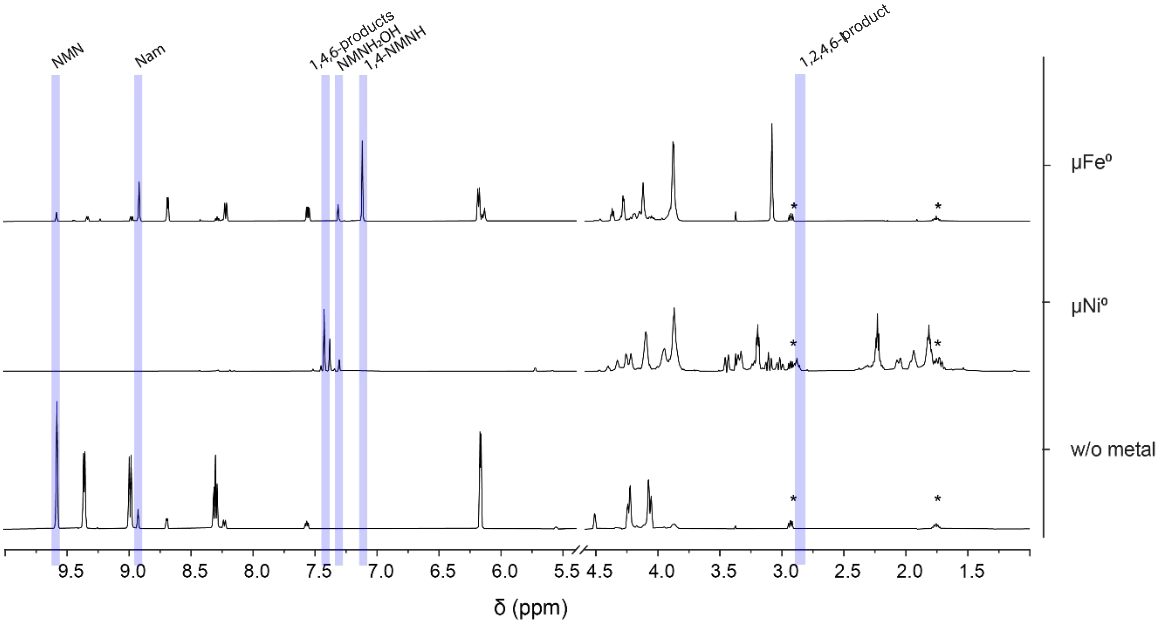

Fig. S51 The NMR spectra of replica samples of 36 μmol NMN in PBS (0.5 M, pH 8.5) with 5 bar of H_2 and 1.8 mmol μFe^0, μNi^0 (50:1 cofactor ratio), or no metal, as shown in **Scheme S14**, are stacked together in this figure. After the 4h reaction, the supernatant was collected and DSS added as an internal standard. The spectra were edited to only include relevant peaks, having been removed a DSS peak at 0 ppm and water peak at 4.8 ppm. No other peaks were found in the areas removed. Some DSS peaks are still visible (*). The peaks used for qualitative analysis and subsequent qNMR are highlighted in blue, according to **Table S8**.

Table S23 After 4 h under 5 bar of Ar, as shown in **Scheme S14**, samples with μNi^0 or μFe^0 yielded different amounts of 1,4-NMNH, $NMNH_2OH$, and nicotinamide (Nam), from the starting material NMN. The starting metal and cofactor were 1.8 mmol and 36 μmol, respectively, mixed in 3 mL of 0.5 M PBS (pH 8.5). The amount of metal atoms was fifty times of the cofactor. The yields were calculated relative to the metal-free sample (100% NMN). To determine the TOF of each reaction, the sum of 1,4-NMNH, 1,4,6-products, and 1,2,4,6-product was considered as the amount of product. All experiments were performed in duplicate.

	Ar	NMN	SD	1,4-NMNH	SD	1,4,6-products	SD	$NMNH_2OH$	SD	1,2,4,6-product	SD	Nam	SD	TOF [s^{-1}]
4h	μNi^0	86.34%	3.1%	0.00%	0.0%	0.00%	0.0%	0.00%	0.0%	0.00%	0.0%	9.48%	1.8%	0.00E+00
	μFe^0	45.66%	2.4%	22.21%	1.2%	0.00%	0.0%	5.44%	0.2%	0.00%	0.0%	22.25%	0.2%	3.34E-07

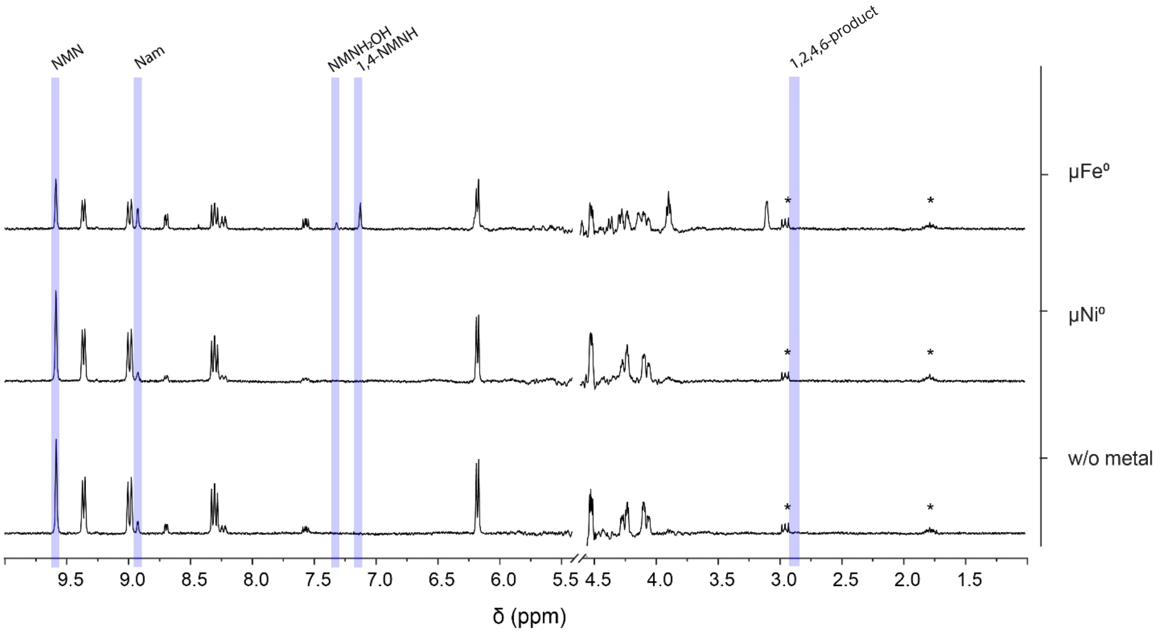

Fig. S52 The NMR spectra of replica samples of 36 μmol NMN in PBS (0.5 M, pH 8.5) with 5 bar of Ar and 1.8 mmol μFe^0, μNi^0 (50:1 cofactor ratio), or no metal, as shown in **Scheme S14**, are stacked together in this figure. After the 4h reaction, the supernatant was collected and DSS added as an internal standard. The spectra were edited to only include relevant peaks, having been removed a DSS peak at 0 ppm and water peak at 4.8 ppm. No other peaks were found in the areas removed. Some DSS peaks are still visible (*). The peaks used for qualitative analysis and subsequent qNMR are highlighted in blue, according to **Table S8**.

“Competition” experiments

To further test the reactivity of NMN and NAD under prebiotic conditions, experiments with both cofactors in the same reaction mixture were designed.

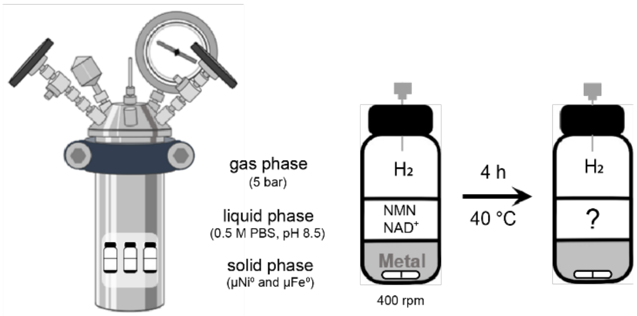

Scheme S15 The reduction of a mixture of NMN and NAD^+ with μFe^0 or μNi^0 was tested with the general protocol described in detail in **Methods**, and according to the scheme above. The amount of cofactor was 72 μmol (36 each, respectively) in 3 mL of pH 8.5 0.5 M PBS buffer, the metal amount was constantly 1.8 mmol (leading to a metal-cofactor ratio of 25:1). The reaction took place for 4 h at 40 °C under slightly alkaline conditions and 5 bars of H_2.

Table S24 After 4 h under 5 bar of H_2, as shown in **Scheme S15**, samples with μFe^0 or μNi^0 yielded different amounts of 1,4-NADH, 1,4-NMNH, $NMNH_2OH$, and nicotinamide (Nam), from the starting material NMN and NAD^+. The starting cofactor mixture was 72 μmol (36 μmol each nucleotide) mixed in 3 mL of 0.5 M PBS (pH 8.5), with a metal-cofactor ratio of 25:1. The yields were calculated separately and relative to the metal-free sample (100% NMN and 100% NAD^+; 12 mM each). To determine the TOF of each reaction, the sum of 1,4-NMNH, 1,4,6-products, 1,2,4,6-product, 1,4-NADH, and 1,6-NADH was considered as part of the reduction products. The NMN and NAD products are presented separately in order for the table to fit the page, but belong to the same set of triplicates.

	H_2	**1,4-NMNH**	**SD**	**1,4,6-products**	**SD**	**$NMNH_2OH$**	**SD**	**1,2,4,6-product**	**SD**	**NMN**	**SD**
4h	μNi^0	5,70%	0,92%	3,74%	0,9%	7,33%	0,3%	0,00%	0,0%	79,55%	1,8%
		**1,4-NADH**	**SD**	**1,6-NADH**	**SD**	**NAD^+**	**SD**	**TOF [s^{-1}]**			
		13,63%	0,8%	3,51%	0,4%	63,8%	17,1%	3,90E-07			
	H_2	**1,4-NMNH**	**SD**	**1,4,6-products**	**SD**	**$NMNH_2OH$**	**SD**	**1,2,4,6-product**	**SD**	**NMN**	**SD**
	μFe^0	7,06%	0,9%	0,00%	0,0%	2,26%	0,7%	0,00%	0,0%	75,15%	11,3%
		**1,4-NADH**	**SD**	**1,6-NADH**	**SD**	**NAD^+**	**SD**	**TOF [s^{-1}]**			
		11,33%	1,2%	2,17%	0,3%	45,1%	10,6%	3,02E-07			

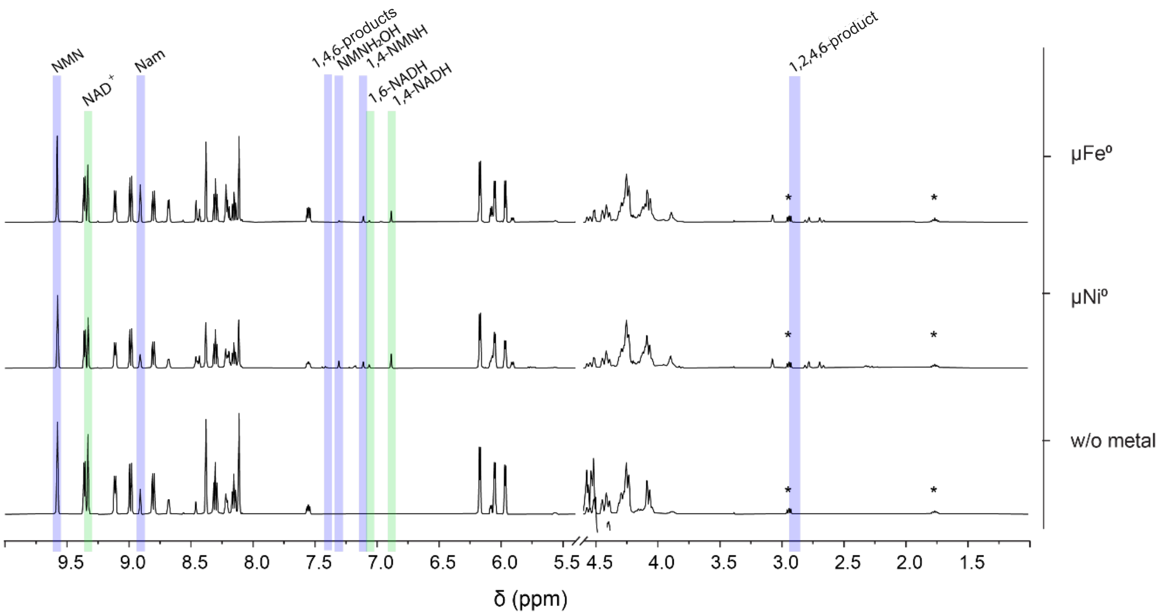

Fig. S53 The NMR spectra of replica samples with a mixture of 36 µmol NMN and 36 µmol NAD^+ in PBS (0.5 M, pH 8.5) with 5 bar of H_2 and 1.8 mmol μFe^0, μNi^0 (25:1 metal-cofactor ratio), or no metal, as shown in **Scheme S15**, are stacked together in this figure. After the 4h reaction, the supernatant was collected and DSS added as an internal standard. The spectra were edited to only include relevant peaks, a DSS peak at 0 ppm and the water peak at 4.8 ppm have been removed. No other peaks were found in the areas removed. Other DSS peaks are still visible (*). The peaks used for qualitative analysis and subsequent qNMR are highlighted in blue (NMN products) and green (NAD products), according to **Table S1** and **Table S8**.

Abiotic oxidation of organic cofactors and reduction of pyruvate

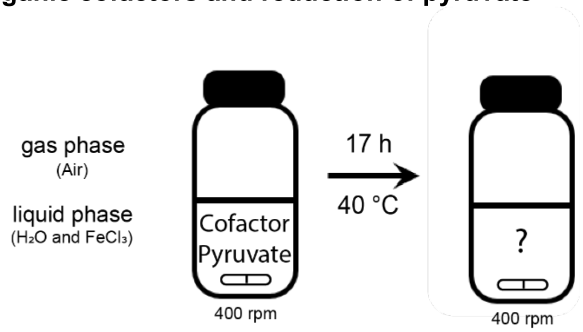

Scheme S16 An aqueous mixture of 0.1 mL with 100 mM pyruvate, twice as much 1,4-NADH or 1,4-NMNH, and 60 mM $FeCl_3$ reacted overnight at 40 °C and 400 rpm to produce lactate, according to the scheme. The pH before and after the reaction is <5.

Table S25 After 17 h with $FeCl_3$, as shown in **Scheme S16**, samples with NMNH and NADH yielded similar amounts of lactate from pyruvate. The starting cofactor was 20 µmol mixed in 0.1 mL of water, with 6 µmol of $FeCl_3$ reacting overnight at 40 °C and 400 rpm (pH<5). The conditions were done in duplicates and yields were calculated relative to the initial amount of pyruvate.

	Air	lactate	SD
NMNH	$FeCl_3$	10,84%	1,5%
NADH		13,03%	1,6%

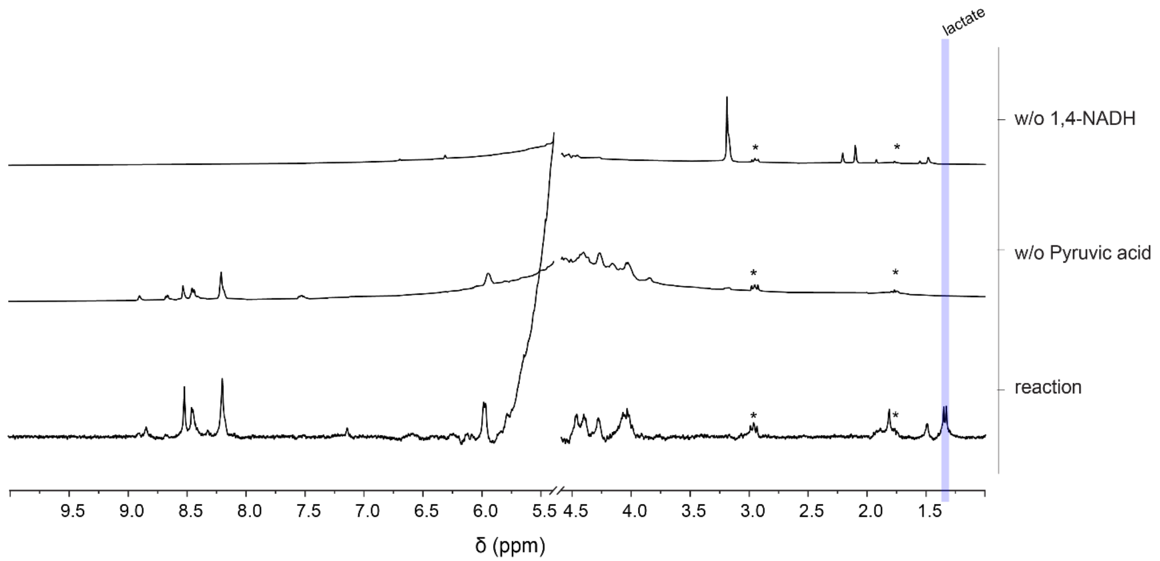

Fig. S54 A NMR spectrum of a replica sample of aqueous 1,4-NADH (20 mmol) were mixed in an Eppendorf tube with 10 mmol of pyruvic acid, and 60 mmol of $FeCl_3$, as indicated by **Scheme S16**, is stacked together in this figure with the respective controls. The latter were without NADH and without pyruvic acid as described on the right side of each spectra. The metals were precipitated with 0.2 mL of a thiolate/phosphate solution. The supernatant was collected and DSS added as an internal standard. The spectra were edited to only include relevant peaks, having been removed a DSS peak at 0 ppm and water peak at 4.8 ppm. No other peaks were found in the areas removed. Some DSS peaks are still visible (*). The peaks used for qualitative analysis and subsequent qNMR are highlighted in blue (1.33 ppm).

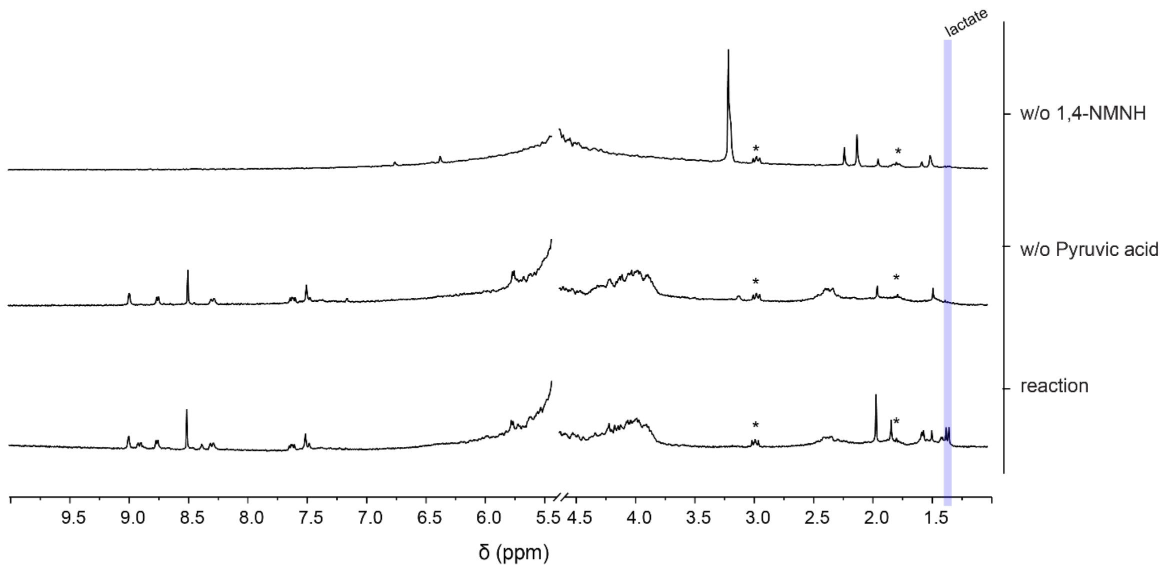

Fig. S55 A NMR spectrum of a replica sample of aqueous NMNH (20 mmol) were mixed in an Eppendorf tube with 10 mmol of pyruvic acid, and 6 mmol of $FeCl_3$, as indicated by **Scheme S16**, is stacked together in this figure with the respective controls. The latter were without NADH and without pyruvic acid as described on the right side of each spectra. The metals were precipitated with 0.2 mL of a thiolate/phosphate solution. The supernatant was collected and DSS added as an internal standard. The spectra were edited to only include relevant peaks, having been removed a DSS peak at 0 ppm and water peak at 4.8 ppm. No other peaks were found in the areas removed. Some DSS peaks are still visible (*). The peaks used for qNMR are highlighted in blue (1.33 ppm).

Reduction of NAD/NMN at pH 5.5

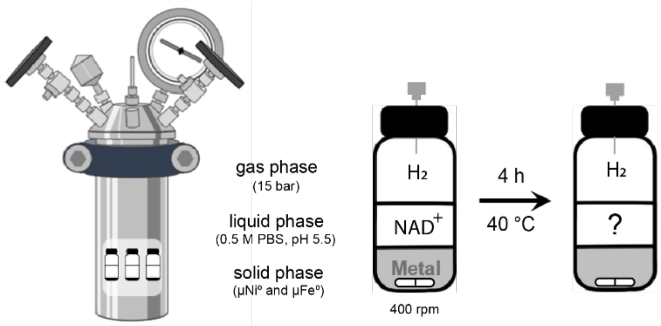

Scheme S17 The reduction of NAD⁺ with μNi and μFe was tested with the protocol described in detail in **Methods**, and according to the scheme above. The amount of NAD⁺ was 18 μmol, which reacted together for 4 h, at 40 °C, under alkaline conditions and 15 bars of H_2. The metal in excess was 3.6 mmol. The same reaction was made with NADH as control to the stability of the reduced cofactor under acidic conditions (pH 5.5).

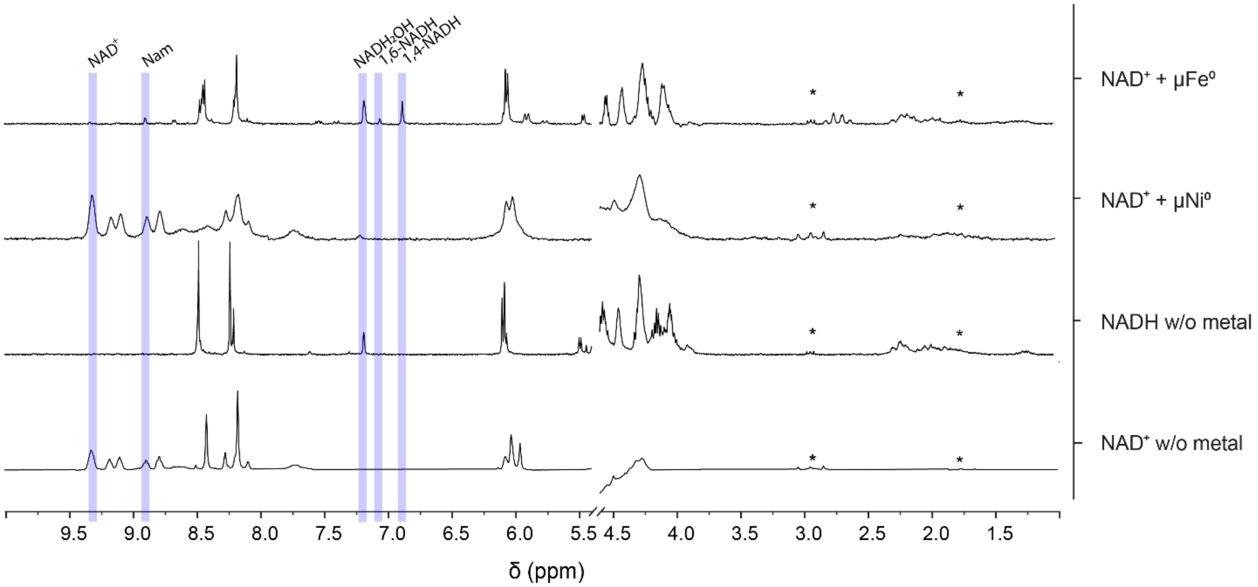

Fig. S56 The NMR spectra of replica samples with 18 μmol NAD⁺ in PBS (0.5 M, pH 5.5) with 15 bar of H_2 and 3.6 mmol μFe^0, μNi^0 (200:1 metal-cofactor ratio), or no metal, as shown in **Scheme S17**, are stacked together in this figure, together with an NADH control under the same conditions. After the 4h reaction, the supernatant was collected and DSS added as an internal standard. The spectra were edited to only include relevant peaks, a DSS peak at 0 ppm and the water peak at 4.8 ppm have been removed. Other DSS peaks are still visible (*). The peaks used for qualitative analysis are highlighted in blue (NAD products), according to **Table S1**.

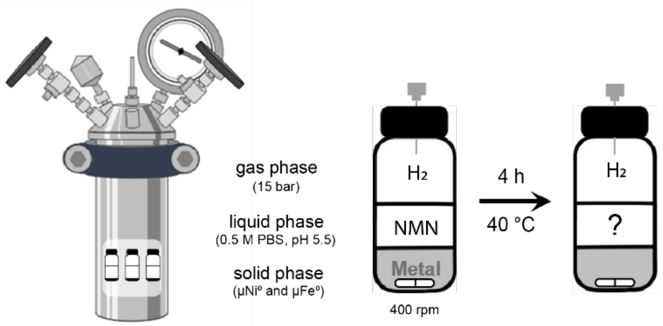

Scheme S18 The reduction of NMN with μNi and μFe was tested with the protocol described in detail in **Methods**, and according to the scheme above. The amount of NMN was 18 μmol, which reacted together for 4 h, at 40 °C, under alkaline conditions and 15 bars of H_2. The metal in excess was 3.6 mmol. The same reaction was made with NMNH as control to the stability of the reduced cofactor under acidic conditions (pH 5.5).

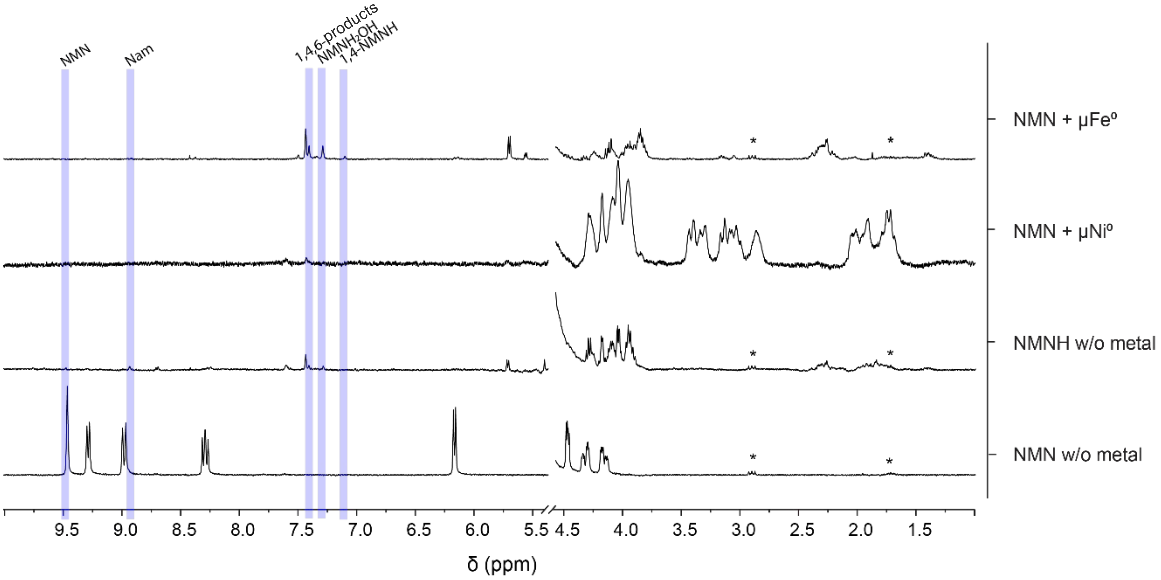

Fig. S57 The NMR spectra of replica samples with 18 μmol NMN in PBS (0.5 M, pH 5.5) with 15 bar of H_2 and 3.6 mmol μFe^0, μNi^0 (200:1 metal-cofactor ratio), or no metal, as shown in **Scheme S18**, are stacked together in this figure, together with an NMNH control under the same conditions. After the 4h reaction, the supernatant was collected and DSS added as an internal standard. The spectra were edited to only include relevant peaks, a DSS peak at 0 ppm and the water peak at 4.8 ppm have been removed. Other DSS peaks are still visible (*). The peaks used for qualitative analysis are highlighted in blue (NMN products), according to **Table S8.**

Standards

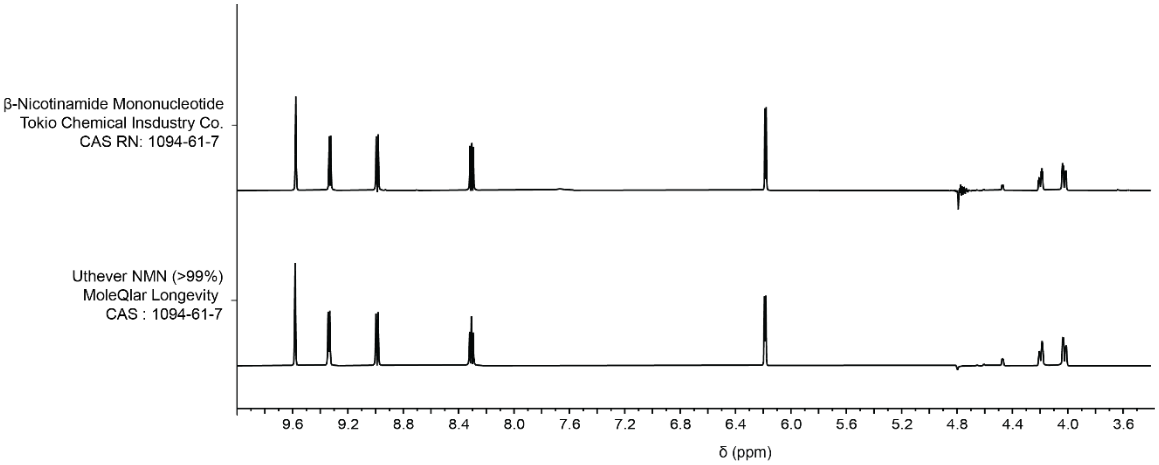

Fig. S58 ^{1}H-NMR comparison of the two different NMN supplies used in all experiments (12 mM of NMN in 0.133 M PBS pH 8.5). Empty spectra was cut out on both extremities, and water suppression hides the peak around 4.8.

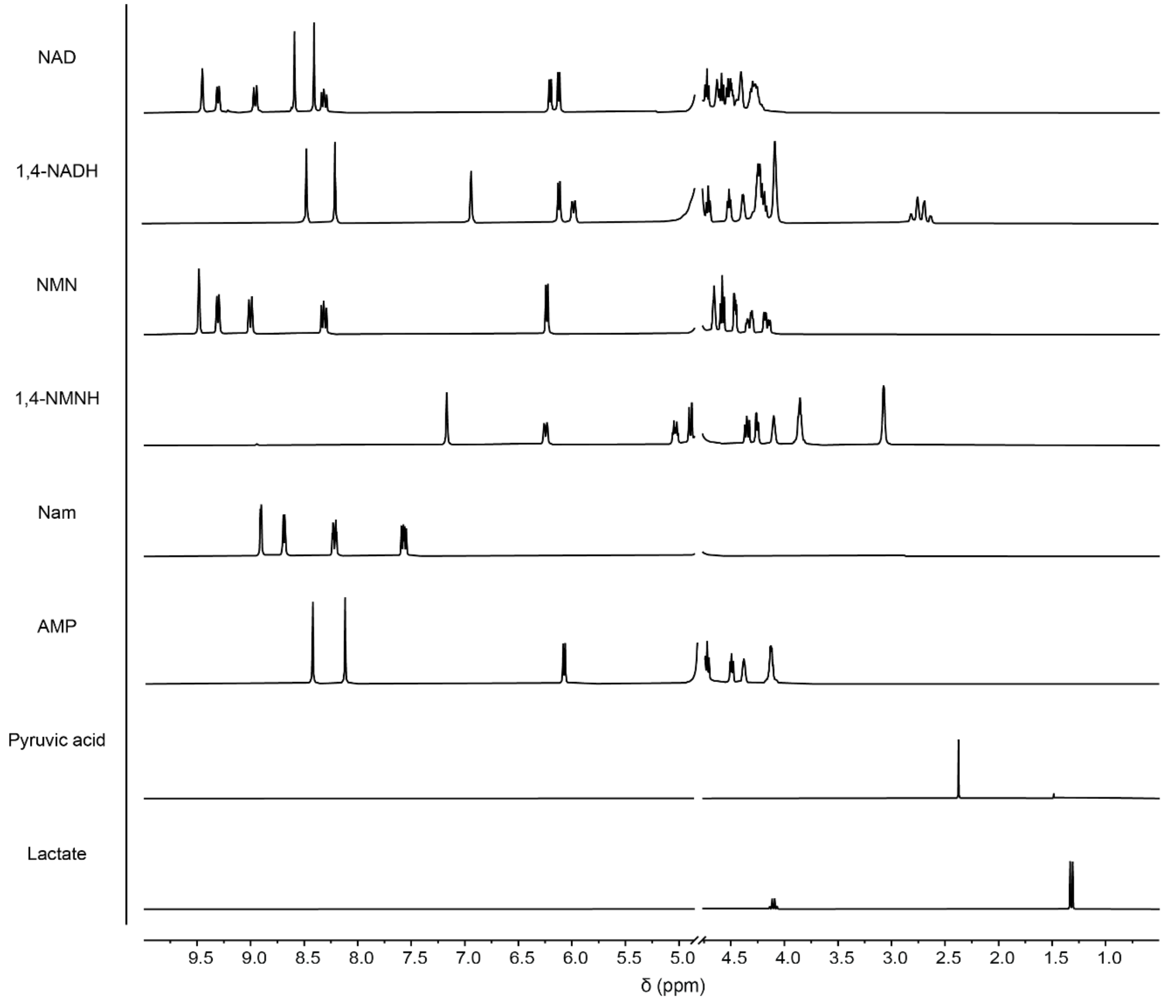

Fig. S59 ^{1}H-NMR standards of several molecules in D_2O. Empty spectra was cut out on both sides, as well as the water peak between 4.5 and 5 ppm.

Characterization of NMN

A full assignment of the ^1H and ^{13}C resonance signals of NMN was fulfilled and the chemical shifts are listed in **Table S26**.

Table S26 NMR data of NMN in D_2O at 298 K

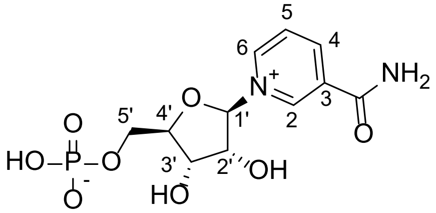

Position	δ_H (multi., *J*)	δ_C
2	9.47 (s)	142.6
3		136.7
C=O		168.6
4	8.99 (dt, 8.2, 1.1)	148.7
5	8.30 (dd, 8.0, 6.4)	131.3
6	9.29 (d, 6.2)	145.2
1'	6.22 (d, 5.6)	102.7
2'	4.34 (dd, 7.6, 5.3)	80.5
3'	4.45 (dd, 5.1, 2.5)	73.8
4'	4,65 (q, 2.5)	90.2 (d, 8.8)
5'	4.31 (ddd, 12.0, 4.3, 2.5)	66.9 (d, 4.9)
	4.15 (ddd, 12.0, 5.0, 2.1)	

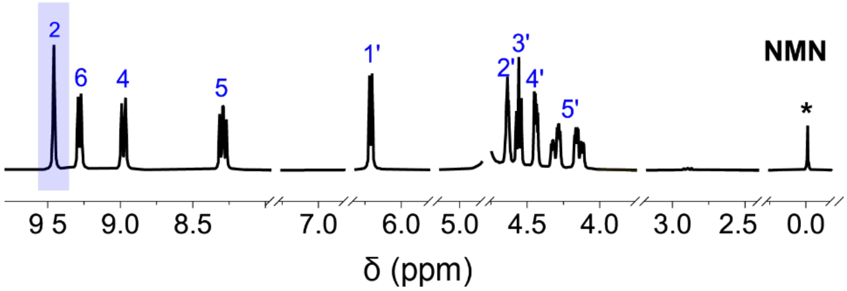

Fig. S60 ^1H spectrum of NMN standard in D_2O with internal reference DSS at 298 K. Empty spectra was removed, highlighting the substrate peaks, labelled according to **Table S26**. Highlighted in blue is the peak used for qNMR. The standard DSS peak at 0 ppm is included and marked (*).

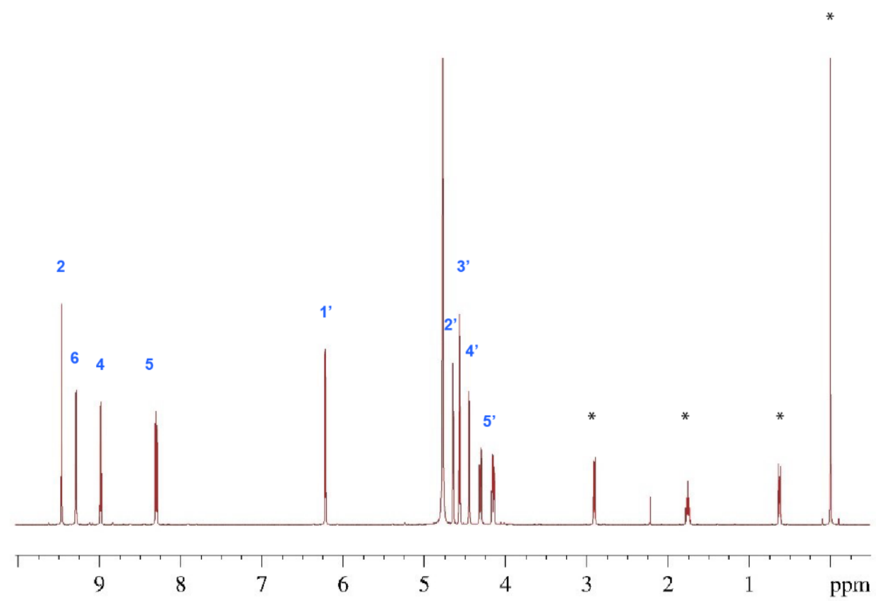

Fig. S61 Complete view of the ^{1}H spectrum of NMN standard in D_2O with internal reference DSS (*) at 298 K, labelled according to Table S1. Some H_2O is visible at about 4.8 ppm.

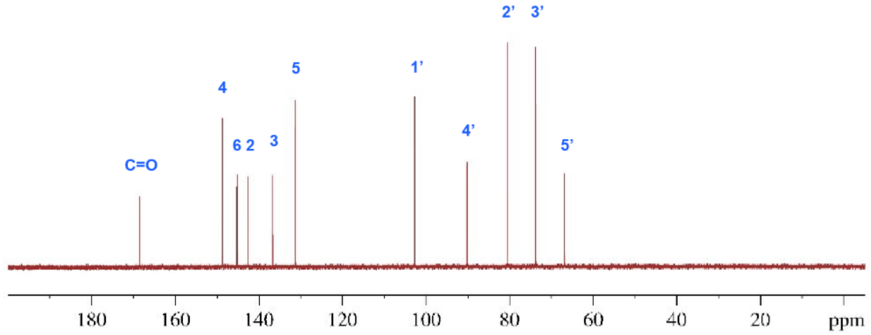

Fig. S62 ^{13}C spectrum of NMN standard in D_2O at 298 K, labelled according to **Table S26**.

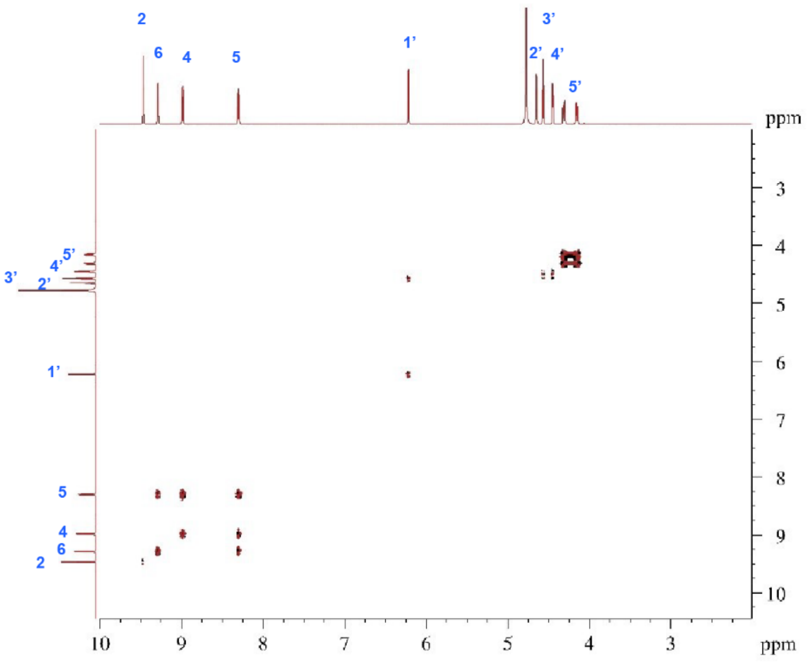

Fig. S63 ^{1}H-^{1}H DQF-COSY spectrum of NMN standard in D_2O at 298 K, labelled according to **Table S26**.

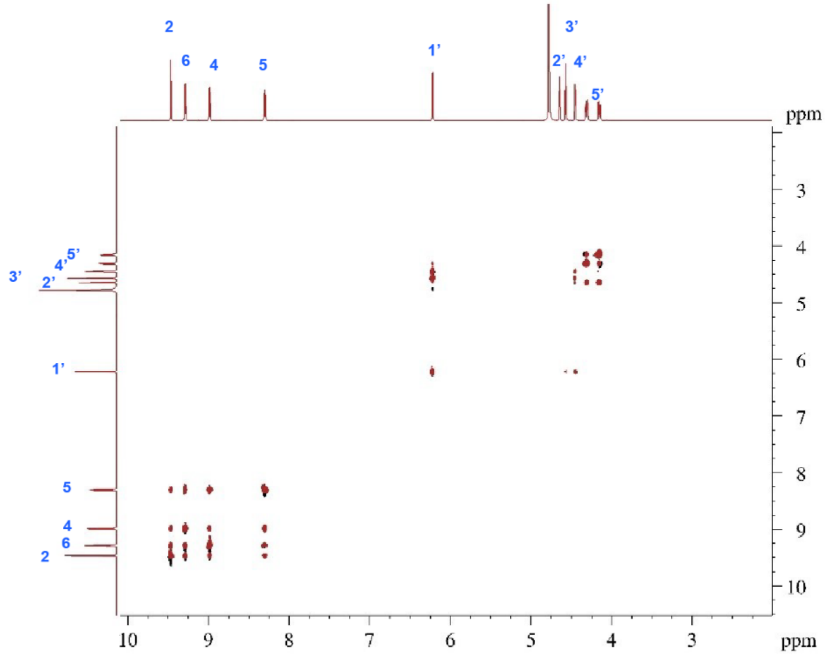

Fig. S64 ^{1}H-^{1}H TOCSY spectrum of NMN standard in D_2O at 298 K, labelled according to **Table S26**.

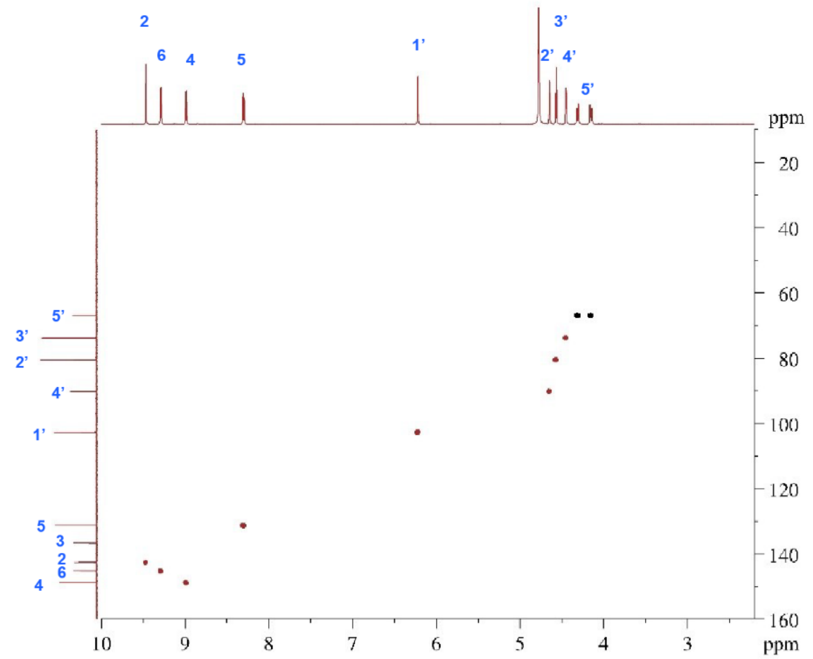

Fig. S65 Edited ^{1}H-^{13}C HSQC spectrum of standard NMN standard in D_2O at 298 K, labelled according to **Table S26**.

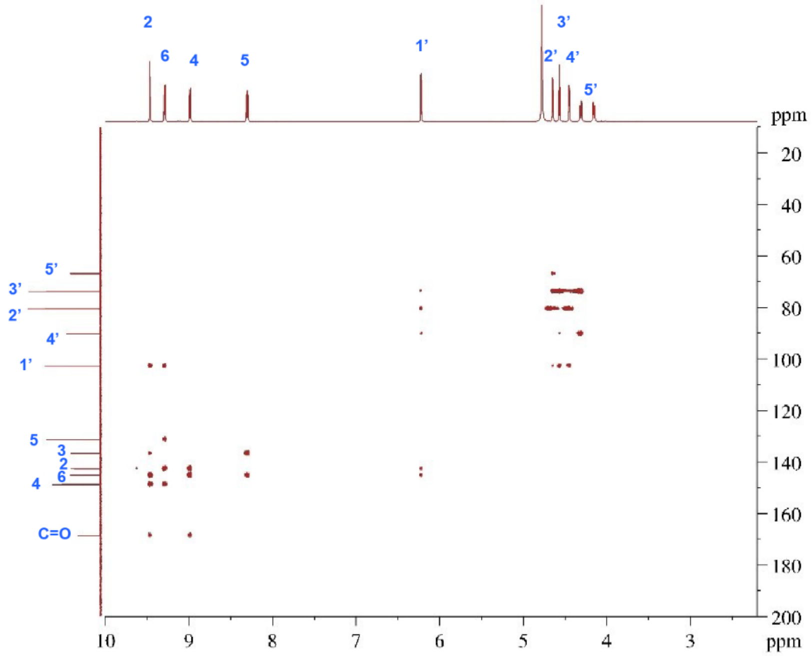

Fig. S66 The ^{1}H-^{13}C HMBC spectrum of NMN standard in D_2O at 298 K, labelled according to **Table S26**.

Characterization of 1,4-NMNH

A full assignment of the ^1H and ^{13}C resonance signals of 1,4-NMNH was fulfilled and the chemical shifts are listed in.

Table S27 NMR data 1,4-NMNH in D_2O at 298 K

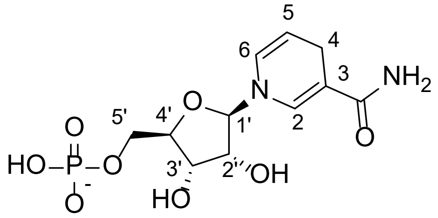

Position	δ_H (multi., J)	δ_C
2	7.15 (d, 1,6)	141.1
3		103.4
C=O		175.9
4	3.056 (d, 1.7); 3.062 (d, 1.7)	24.7
5	5.02 (m)	108.2
6	6.24 (ddt, 8.3, 1,4, 1.5)	127.7
1'	4.88 (m)	97.7
2'	4.34 (dd, 7.6, 5.3)	73.3
3'	4.25 (dd, 5.3, 2.0)	73.6
4'	4.09 (m)	86.0 (d, 8.8)
5'	3.87 – 3.81 (m)	66.6 (d, 4.1)

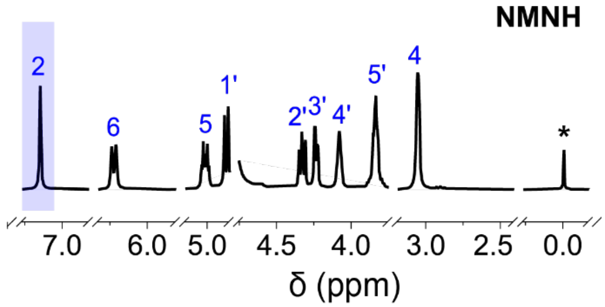

Fig. S67 ^1H spectrum of NMNH standard in D_2O with internal reference DSS at 298 K. Empty spectra was removed, highlighting the substrate peaks, labelled according to **Table S27** highlighted in blue is the peak used for qNMR.

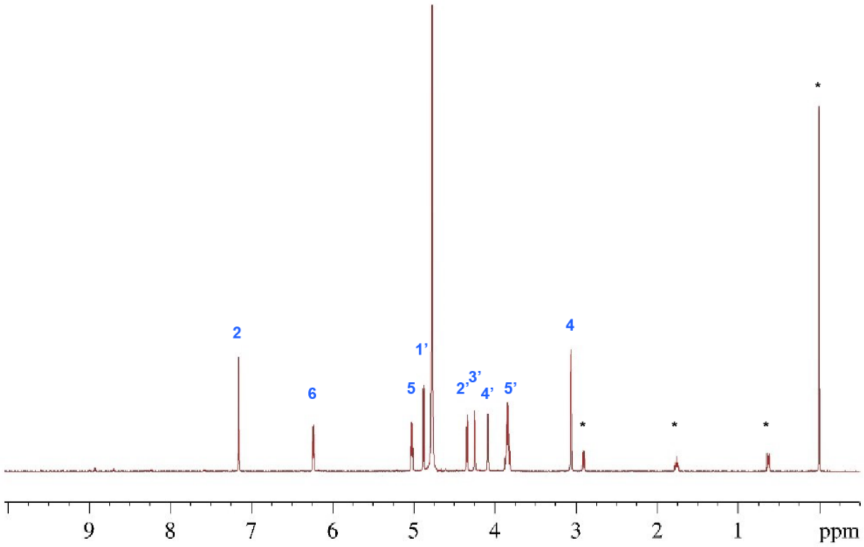

Fig. S68 Complete view of the ^{1}H spectrum of NMN standard in D_2O with internal reference DSS (*) at 298 K, labelled according to **Table S27**. Some H_2O is visible at about 4.8 ppm.

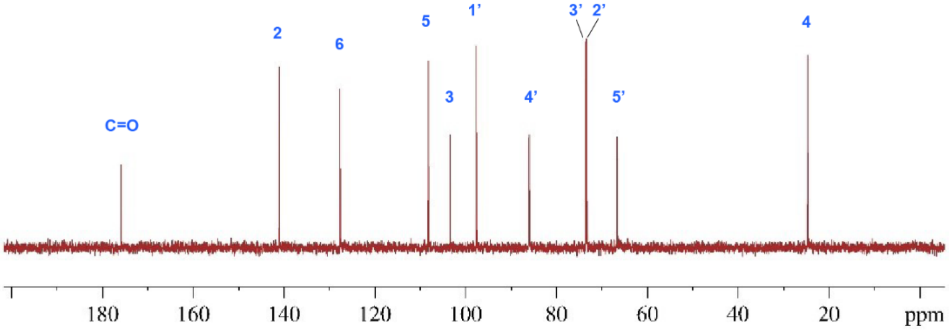

Fig. S69 ^{13}C spectrum of 1,4-NMNH in D_2O at 298 K, labelled according to **Table S27**.

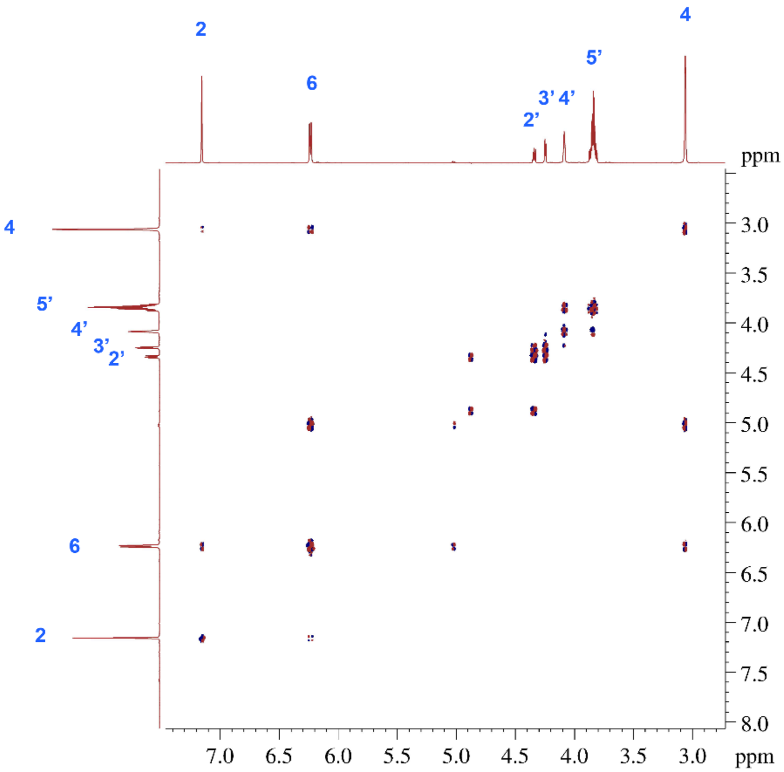

Fig. S70 ¹H-¹H DQF-COSY spectrum of 1,4-NMNH in D_2O at 298 K, labelled according to **Table S27**.

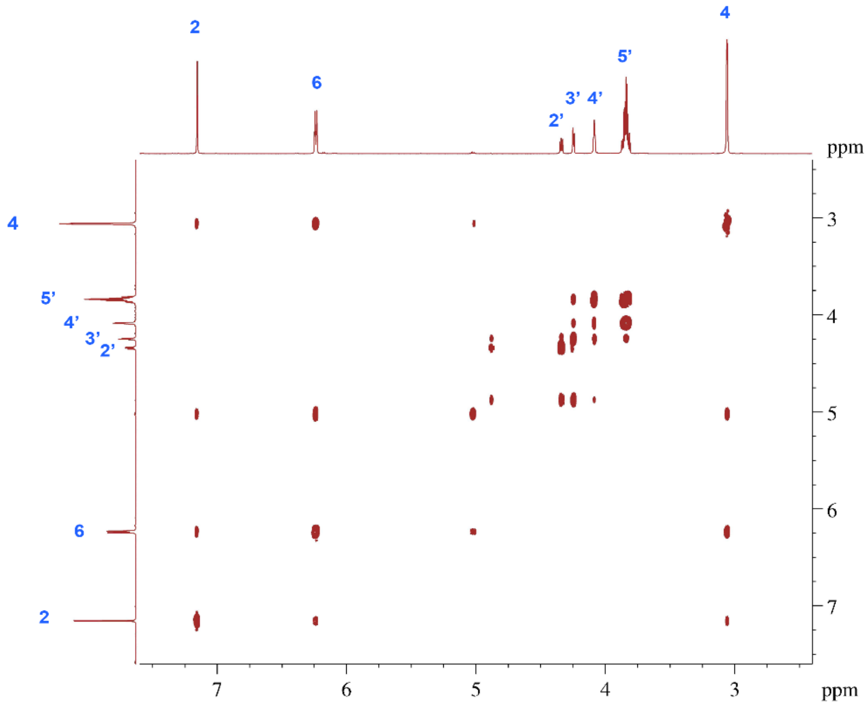

Fig. S71 ¹H-¹H TOCSY spectrum of 1,4-NMNH in D_2O at 298 K, labelled according to **Table S27**.

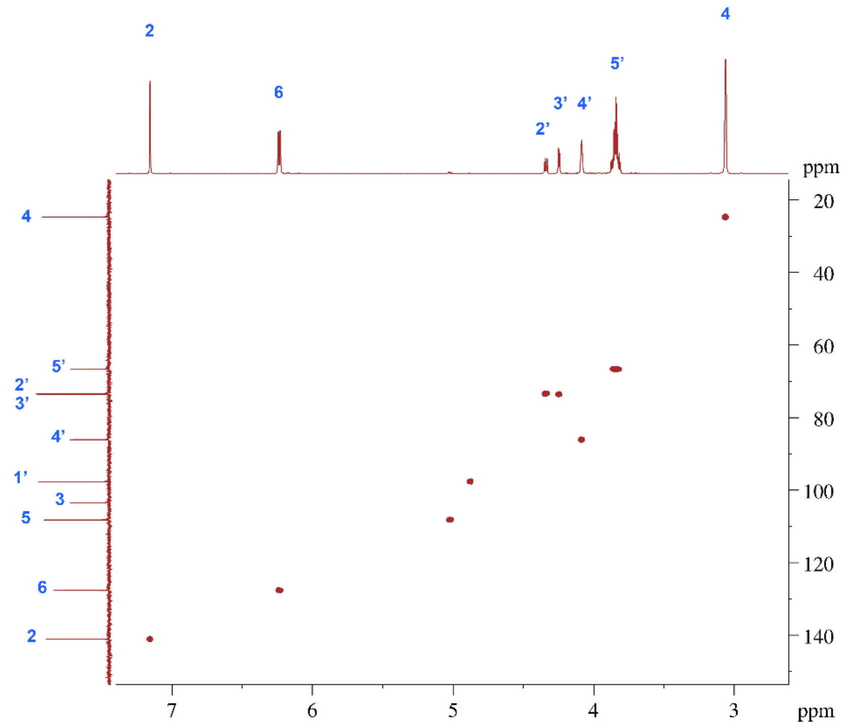

Fig. S72 Edited ^{1}H-^{13}C HSQC spectrum of 1,4-NMNH in D_2O at 298 K, labelled according to **Table S27**.

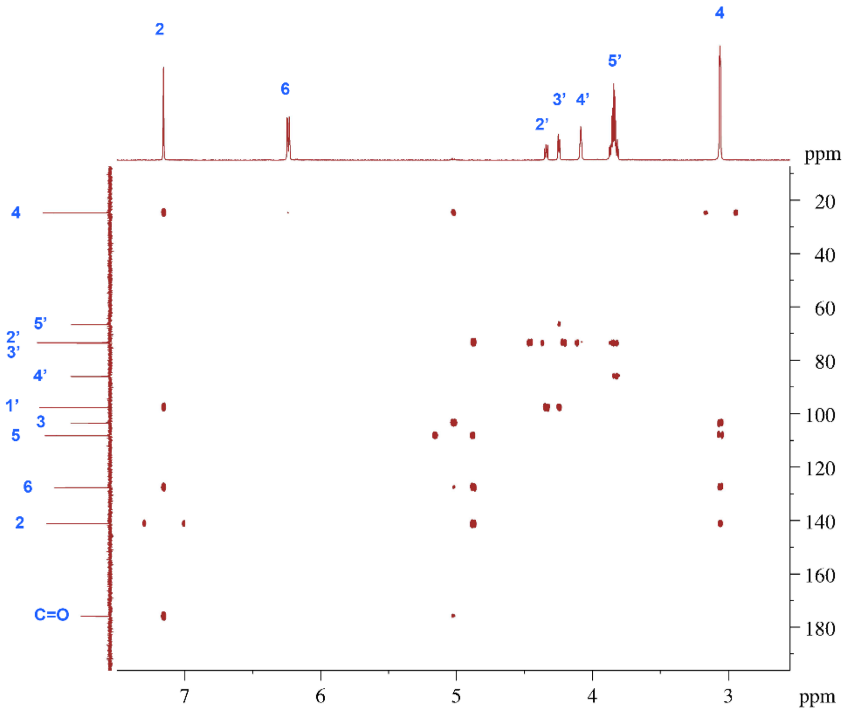

Fig. S73 The ^{1}H-^{13}C HMBC spectrum of 1,4-NMNH in D_2O at 298 K, labelled according to **Table S27**.

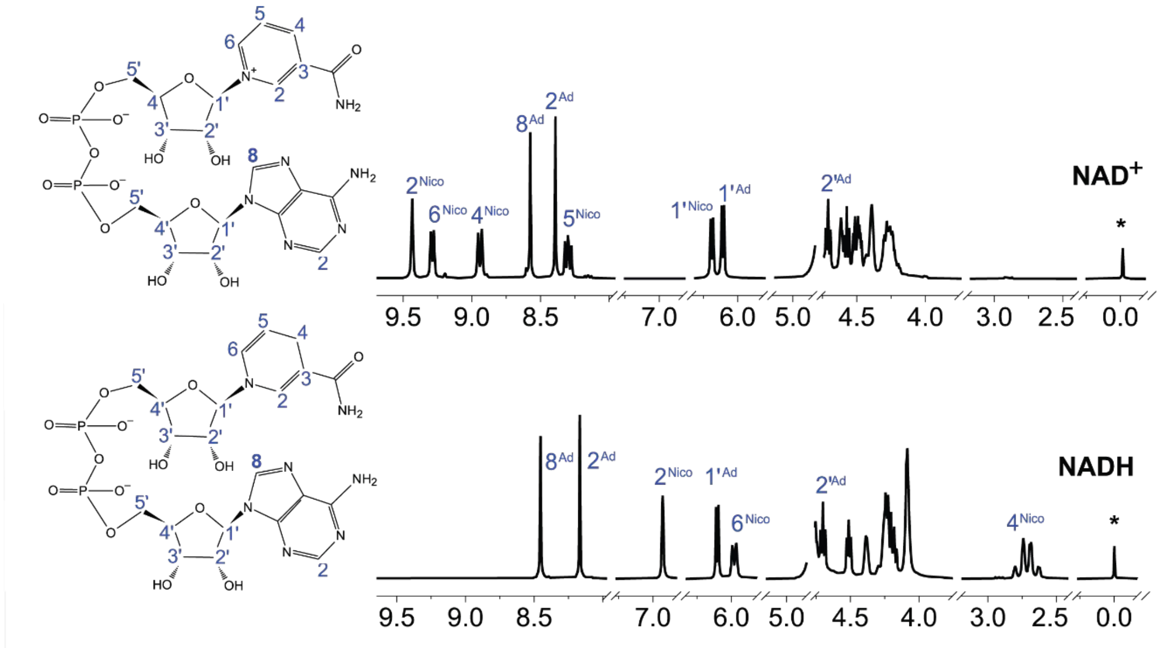

Fig. S74 According to 2D-NMR measurements and literature, the 1H-NMR peaks obtained from NAD^+ (above) and NADH (bellow) can be assigned to hydrogens bound to the carbons indicated in the figure. Only spectra without any significant peaks were removed from the figure at several ppm values. Highlighted in blue is the peak used for qNMR. The standard DSS peak at 0 ppm is included and marked (*).

Supplementary References

1. Choi, Y. K. *et al.* CHARMM-GUI nanomaterial modeler for modeling and simulation of nanomaterial systems. *J. Chem. Theory Comput.* **18**, 479–493 (2022).
2. Heinz, H., Vaia, R. A., Farmer, B. L. & Naik, R. R. Accurate simulation of surfaces and interfaces of face-centered cubic metals using 12−6 and 9−6 Lennard-Jones potentials. *J. Phys. Chem. C* **112**, 17281–17290 (2008).
3. Kim, S. *et al.* CHARMM-GUI ligand reader and modeler for CHARMM force field generation of small molecules. *J. Comput. Chem.* **38**, 1879–1886 (2017).
4. Best, R. B. *et al.* Optimization of the additive CHARMM all-atom protein force field targeting improved sampling of the backbone ϕ, ψ and side-chain χ_1 and χ_2 dihedral angles. *J. Chem. Theory Comput.* **8**, 3257–3273 (2012).
5. Li, P. Advances in metal ion modeling. (Michigan State University, 2016).
6. Berendsen, H. J. C., Van Der Spoel, D. & Van Drunen, R. GROMACS: A message-passing parallel molecular dynamics implementation. *Computer Physics Communications* **91**, 43–56 (1995).
7. Hunter, J. D. Matplotlib: A 2D graphics environment. *Comput. Sci. Eng.* **9**, 90–95 (2007).
8. Humphrey, W., Dalke, A. & Schulten, K. VMD: Visual molecular dynamics. *Journal of Molecular Graphics* **14**, 33–38 (1996).
9. Henriques Pereira, D. P. *et al.* Role of geochemical protoenzymes (geozymes) in primordial metabolism: Specific abiotic hydride transfer by metals to the biological redox cofactor NAD^+. *The FEBS Journal* 16329 (2022) doi:10.1111/febs.16329.
10. Ernst, R. R., Bodenhausen, G. & Wokaun, A. *Principles of Nuclear Magnetic Resonance in one and two dimensions*. (Clarendon Press, Oxford, 2004).
11. Wüthrich, K. *NMR of proteins and nucleic acids*. (Wiley, New York, 1986).
12. Evans, J. N. S. *Biomolecular NMR spectroscopy*. (Oxford University Press, Oxford, 1996).
13. Kalinowski, H.-O., Berger, S. & Braun, S. *^{13}C-NMR-spektroskopie*. (Georg Thieme Verlag, Stuttgart, 1988).
14. Acheson, R. M. & Paglietti, G. Reduction of some I -substituted pyridinium salts. *Journal of the Chemical Society, Perkin Transactions 1* **1**, 45–48 (1976).
15. Barrett, S. M., Pitman, C. L., Walden, A. G. & Miller, A. J. M. Photoswitchable hydride transfer from iridium to 1-methylnicotinamide rationalized by thermochemical cycles. *J. Am. Chem. Soc.* **136**, 14718–14721 (2014).
16. Godtfredsen, S. E., Ottesen, M. & Andersen, N. R. On the mode of formation of 1,6-dihydro-NAD in NADH preparations. *Carlsberg Res. Commun.* **44**, 65–75 (1979).
17. Meyer, J. *et al.* Experimental insights into electrocatalytic $[Cp^*Rh(bpy)Cl]^+$ mediated NADH regeneration. *Sci Rep* **13**, 22394 (2023).
